# Halide Perovskite Photocatalysts for Clean Fuel Production and Organic Synthesis: Opportunities and Challenges

**DOI:** 10.1002/adma.202419603

**Published:** 2025-05-09

**Authors:** Siddharth Singh, Zeinab Hamid, Ramavath Babu, Sergio Gómez‐Graña, Xiaowen Hu, Iain McCulloch, Robert L. Z. Hoye, Vishal Govind Rao, Lakshminarayana Polavarapu

**Affiliations:** ^1^ Department of Chemistry Indian Institute of Technology Kanpur Kanpur Uttar Pradesh 208016 India; ^2^ Department of Chemistry Chemistry Research Laboratory University of Oxford Oxford OX1 3TA UK; ^3^ Andlinger Center for Energy and the Environment and Department of Electrical and Computer Engineering Princeton University Princeton NJ 08544 USA; ^4^ CINBIO Universidade de Vigo Campus Universitario As Lagoas‐Marcosende Vigo 36310 Spain; ^5^ SCNU‐TUE Joint Lab of Device Integrated Responsive Materials (DIRM) Guangdong Provincial Key Laboratory of Optical Information Materials and Technology National Center for International Research on Green Optoelectronics South China Academy of Advanced Optoelectronics South China Normal University Guangzhou 510006 China; ^6^ Inorganic Chemistry Laboratory Department of Chemistry University of Oxford South Parks Road Oxford OX1 3QR UK

**Keywords:** CO_2_ reduction, halide perovskites, photocatalysis, solar‐to‐fuel production, water splitting

## Abstract

The need to constrain the use of fossil fuels causing global warming is motivating the development of a variety of photocatalysts for solar‐to‐fuel generation and chemical synthesis. In particular, semiconductor‐based photocatalysts have been extensively exploited in solar‐driven organic synthesis, carbon dioxide (CO_2_) conversion into value‐added products, and hydrogen (H_2_) generation from water (H_2_O) splitting. Recently, metal halide perovskites (MHPs) have emerged as an important class of semiconductors for heterogeneous photocatalysis owing to their interesting properties. Despite key issues with long‐term stability and degradation in polar solvents due to their ionic character, there has been significant progress in halide perovskite‐based photocatalysts with improving their stability and performance in the gas and liquid phases. This review discusses the state‐of‐the‐art for using halide perovskite‐based photocatalysts and photoelectrocatalysis in hydrogen production from water and halogen acid solutions, CO_2_ reduction into value‐added chemicals, and various organic chemical transformations. The different types of halide perovskites used, design strategies to overcome the instability issues in polar solvents, and the efficiencies achieved are discussed. Furthermore, the outstanding challenges associated with the use of polar electrolytes and how the stability and performance can be improved are discussed.

## Graphical abstract

This review provides the state‐of‐the‐art overview of halide perovskite‐based photocatalysts for hydrogen production from water and halogen acid solutions, CO_2_ reduction into value‐added chemicals, and organic chemical transformations. Different types of halide perovskite photocatalysts and their relative efficiencies for hydrogen generation and CO_2_ reduction, design strategies to overcome the instability issues in polar solvents and outstanding challenges are discussed.

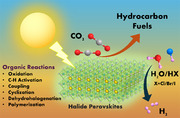

## 1 Introduction

Global warming caused by burning fossil fuels is one of the world's most significant concerns.^[^ ^1^, ^2^ ^]^ The annual release of CO_2_ equivalent (CO_2_eq) emissions (>37 tonnes per year in 2023) has been increasing each year and already exceeds the amount consumed by plants for photosynthesis.^[^ ^3^ ^]^ Therefore, clean energy generation from renewable energy sources, as well as effective approaches for CO_2_ capture and removal, are crucial to limit the effects of climate change.^[^ ^4^, ^5^ ^]^ Solar energy is the most abundant source of clean energy.^[^ ^6^, ^7^, ^8^ ^]^ Harvesting solar energy can be used not only to produce clean electricity, but also to decarbonize the chemicals industry, producing fuels sustainably. These fuels are needed for transport, the production of plastics, as well as fertilizer, among many other examples. Hence, alongside the need for electrification, it is equally important to shift towards zero‐ or low‐emission fuels, such as hydrogen, or net‐zero fuels, such as hydrocarbon fuels generated from captured CO_2_.^[^ ^9^ ^]^ Hydrogen as a fuel source can be burnt in a combustion engine or used in a fuel cell, with the latter constituting a “cleaner” option,^[^ ^10^ ^]^ since the only by‐product is water. Hydrogen is classified into various categories depending on the way it is sourced, two of which are *grey hydrogen* and *green hydrogen*. The former relies on the use of natural gas, and there is therefore a non‐zero CO_2_eq footprint associated with it. In contrast, green hydrogen refers to H_2_ produced by splitting water using clean energy sources, such as by harvesting solar energy. This involves the use of a photocatalyst that absorbs light to generate free charge carriers which then drive the required chemical reactions. Considering the whole lifecycle, hydrogen is not necessarily the most sustainable source of energy. Green hydrogen production requires the input of electrical energy. Reconverting H_2_ into electrical energy further downstream constitutes a significant overall energy loss. Whilst the direct use of electricity may be more efficient in certain applications (e.g., heating), H_2_ nevertheless holds importance as a feedstock in several industries on which modern‐day life depends, the most important of which are fertilizer synthesis, oil refining, and steel production. In 2022, green hydrogen constituted only 3% of all hydrogen produced worldwide, while grey hydrogen constituted nearly 96%.^[^ ^11^ ^]^

Another way of storing solar energy in green fuels is by making hydrocarbons via CO_2_ capture and reduction.^[^ ^12^ ^]^ While these fuels do not contribute to decarbonizing the energy sector, they are critical for achieving net‐zero CO_2_eq emissions goals. Although the specific energy density (MJ kg^−1^) of H_2_ is three times that of gasoline,^[^ ^13^, ^14^ ^]^ synthetic hydrocarbon fuels offer advantages in that their use requires no change to existing infrastructure, such as pipelines for gas transport. This is in contrast with the significant costs associated with building new specialized networks or adapting existing ones to mitigate the risks of structural damage due to hydrogen exposure (e.g., hydrogen embrittlement of steels).^[^ ^15^ ^]^ Furthermore, photocatalytic conversion of CO_2_ into chemical fuels like ethanol does not pose a threat to food security as in the case of bioethanol, which competes for agricultural land.^[^ ^16^ ^]^ Nonetheless, CO_2_ reduction can lead to a variety or mixture of products depending on catalyst choice and reaction conditions,^[^ ^17^ ^]^ making selectivity an added issue compared to photocatalytic hydrogen evolution. Furthermore, while CO_2_ reduction to certain alcohols and hydrocarbons is more thermodynamically favorable than proton reduction, given the more positive reduction potentials, these reactions suffer from a kinetic barrier associated with the requirement of multiple proton‐coupled electron transfer steps.^[^ ^18^ ^]^

In addition to using sunlight to drive the production of hydrogen and synthetic fuels, another application of interest is organic synthesis. Indeed, the realization of the importance of photochemistry dates back to the early 1900s, when scientists recognized the need to optimize synthetic processes by relying on light energy rather than conditions involving high temperatures and harsh chemicals.^[^ ^19^ ^]^ In addition to optimized reaction conditions, photochemistry also opens the door to previously inaccessible transformations, since the reactivity of molecules in the excited state differs from that in the ground state.^[^ ^20^ ^]^

Considering the above, solar‐driven fuel production and chemical synthesis are important. Efforts to drive photoelectrocatalytic and photocatalytic generation of hydrogen and synthetic hydrocarbons date back to the 1970s and 1980s,^[^ ^21^, ^22^, ^23^, ^24^ ^]^ following the pioneering work by Fujishima and Honda in 1972, when they used a photoelectrochemical cell to drive overall water splitting (OWS), with TiO_2_ as a photoanode and Pt as a cathode.^[^ ^24^ ^]^ Since then, research so far has focused primarily on inorganic, wide‐bandgap semiconductors. But the performance of these materials is limited by their inability to harness solar energy in the visible wavelength range, which has significantly higher solar irradiance than in the UV.^[^ ^25^ ^]^ While certain lower‐bandgap inorganic semiconductors can utilize visible light, another bottleneck of these materials is fast charge‐carrier recombination, which limits the supply of photogenerated charge‐carriers for redox reactions.^[^ ^26^, ^27^, ^28^ ^]^ Similarly, various types of photoredox catalysts may be used to catalyze organic synthesis, such as transition metal complexes and organic catalysts. However, these may be limited by their reliance on precious metals, moderate extinction coefficients, as well as challenges with synthesizing these catalysts at scale.^[^ ^29^, ^30^ ^]^

**Figure 1:**
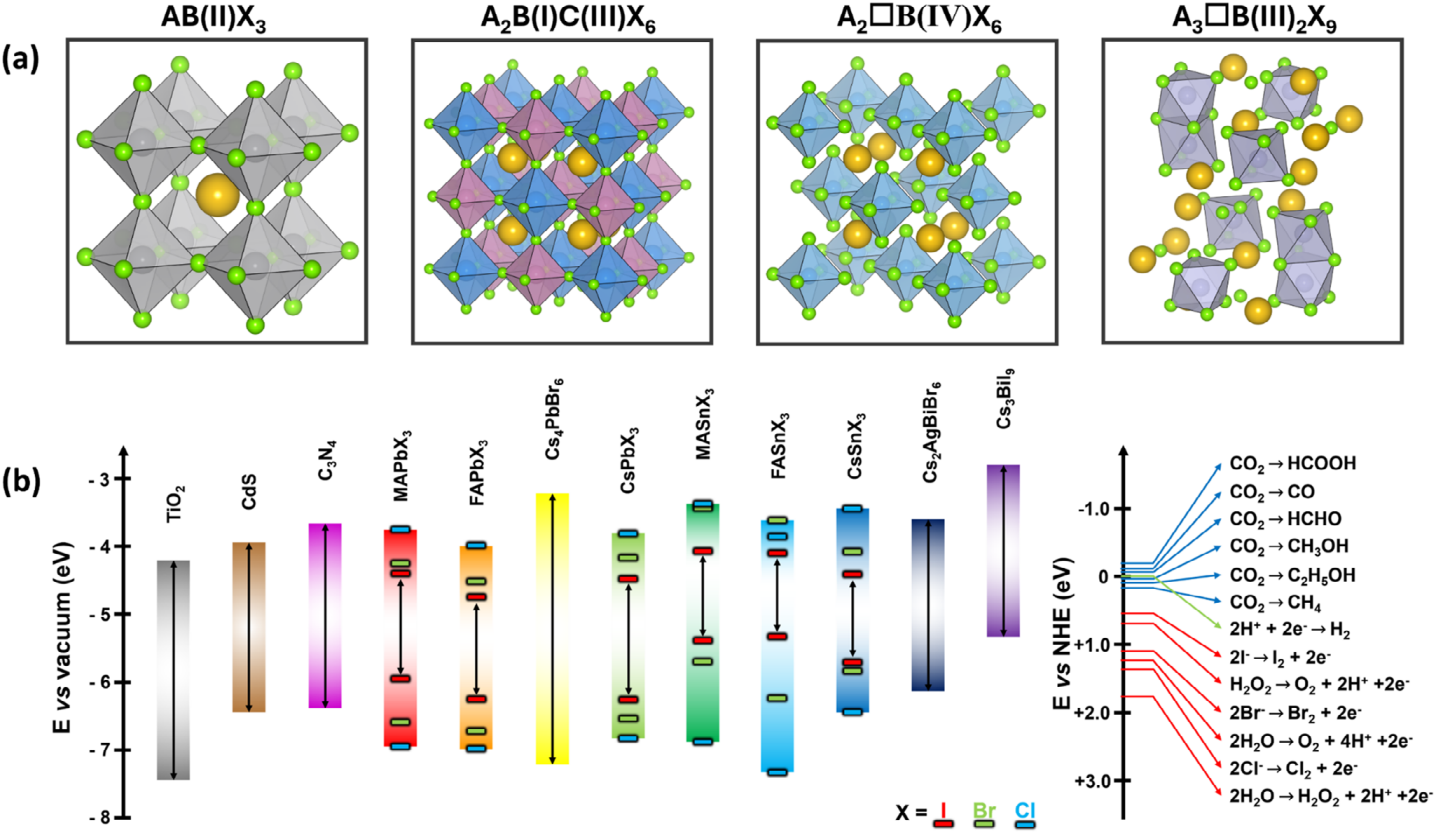
a) Crystal structures of ABX_3_ halide perovskites, A_2_B(I)B(III)X_6_ double perovskites, A_2_◻B(IV)X_6_ vacancy‐ordered perovskites (◻: vacancy), and A_3_B(III)_2_X_6_ 0D perovskite derivatives, which are potential photocatalysts for H_2_ evolution, CO_2_ reduction and organic synthesis.^[^ ^37^ ^]^ The crystal structures were drawn using the VESTA software. b) Band edge positions of different types of perovskite and perovskite‐derivative photocatalysts relative to vacuum and the reversible hydrogen electrode (RHE).^[^ ^38^ ^]^

Over the last decade, metal halide perovskites (MHPs) have garnered significant attention due to their exceptional optoelectronic properties.^[^ ^31^, ^32^ ^]^ They are categorized into two types: organic–inorganic hybrid and all‐inorganic perovskites. They are commonly denoted by the chemical formula ABX_3_, where the A‐site can be either a monovalent inorganic or organic cation (such as Cs^+^ or CH_3_NH_3_^+^), B‐site a divalent cation (like Pb^2+^, Sn^2+^, Sr^2+^, or their combinations), and the X‐site a halide anion (Cl^−^, Br^−^, I^−^, or their combinations) (**Figure** **1**a). The hybrid perovskites can also exist in layered structures. These include Ruddlesden–Popper perovskites, which have the general formula L_2_A_n−1_Pb_n_X_3n+1_, where the L‐site is occupied by a long‐chain organic amine cation (spacer), the A‐site is a short‐chain organic cation, and *n* is an integer.^[^ ^33^ ^]^ Since 2009, when Miyasaka and co‐workers published the first report on the use of 3D MHPs in solar cells,^[^ ^34^ ^]^ a broad set of materials within the halide perovskite family have been used in photovoltaics, demonstrating remarkable efficiencies of up to a certified value of 27.0% in single‐junction cells and 34.6% in perovskite/Si tandem photovoltaics (exceeding the practical limit of single‐junction crystalline Si solar cells of 28%).^[^ ^35^ ^]^ These figures underscore the exceptional light‐absorbing properties of MHPs across a wide spectrum, coupled with their extended charge‐carrier diffusion lengths.^[^ ^31^ ^]^ The properties of MHPs, in terms of their ease of synthesis, tunable bandgaps, defect tolerance, and charge transport, make them promising candidates in solar cells and as photocatalysts to drive solar‐fuel generation and organic synthesis. For instance, the bandgap of MHPs could be finely tuned approximately from 1.2 to 3 eV (Figure 1b).^[^ ^36^ ^]^ This enables the valence and conduction band edges to be optimized to fulfill the thermodynamic requirements to drive a variety of chemical reactions (Figure 1b).

However, despite these favorable characteristics, the ionic nature of MHPs renders them prone to dissolution in moisture and polar solvents, thereby imposing significant challenges to their employment in photocatalytic applications in polar solvents.^[^ ^39^ ^]^ To tackle this issue, various strategies have been used, such as H_2_ generation in saturated hydrohalic acid solution, the use of non‐polar or low‐polar solvents, lowering the structural dimensionality, and relying on heterojunctions or encapsulants.^[^ ^40^, ^41^, ^42^, ^43^, ^44^, ^45^, ^46^ ^]^ The first strategy was pioneered by Park and co‐workers when they relied on the common‐ion effect to produce water‐stable MHPs in aqueous environments to produce H_2_ from HI splitting.^[^ ^47^ ^]^ Employing heterojunctions or encapsulants based on organic ligands, polymers, MOFs, and inorganic materials has also been a strategy used to enhance their stability in water.^[^ ^42^, ^48^, ^49^, ^50^, ^51^, ^52^ ^]^ For example, in recent literature, bola‐amphiphilic ligands (organic ligands) utilized to cap CsPbBr_3_ NCs and successfully employed in a photocatalytic acrylamide polymerization in an aqueous medium.^[^ ^42^, ^53^, ^54^ ^]^ Other than that, few all inorganic MHPs were realized for their inherent stability in polar environments or moisture, attributed to their composition, which includes materials such as Rb_3_Bi_2_I_9_, Cs_3_Bi_2_I_9_, MA_3_Bi_2_I_9_, Cs_2_AgBiBr_6_, among others.^[^ ^55^, ^56^, ^57^, ^58^ ^]^ Nonetheless, while the above approaches can improve stability, photocatalytic activity remains low. Indeed, another bottleneck to overcome is the presence of active sites, whether intrinsic or co‐catalyst incorporation. Such sites are key to reducing the energetic barriers associated with the reactions involved, thus enhancing activities.

Bearing in mind the importance of the photocatalytic applications outlined above, and the pros and cons of MHPs in photocatalysis, this review aims to discuss the research progress in using MHPs as photo(electro)catalysts for hydrogen evolution, CO_2_ reduction, and organic synthesis. While various existing reviews tackle the same topics independently at different levels of depth,^[^ ^59^, ^60^, ^61^, ^62^, ^63^, ^64^, ^65^ ^]^ this review provides a comprehensive overview of halide perovskite‐based photocatalytic applications covering solar‐to‐fuel generation as well as a wide range of organic reactions along with discussions on associated challenges. Especially, the challenges associated with water oxidation are often neglected when focusing on the proton reduction half‐reaction but are key to moving away from relying on sacrificial oxidation half‐reactions to drive fuel synthesis.

## 2 Design Strategies for Perovskite Photocatalysis

**Figure 2:**
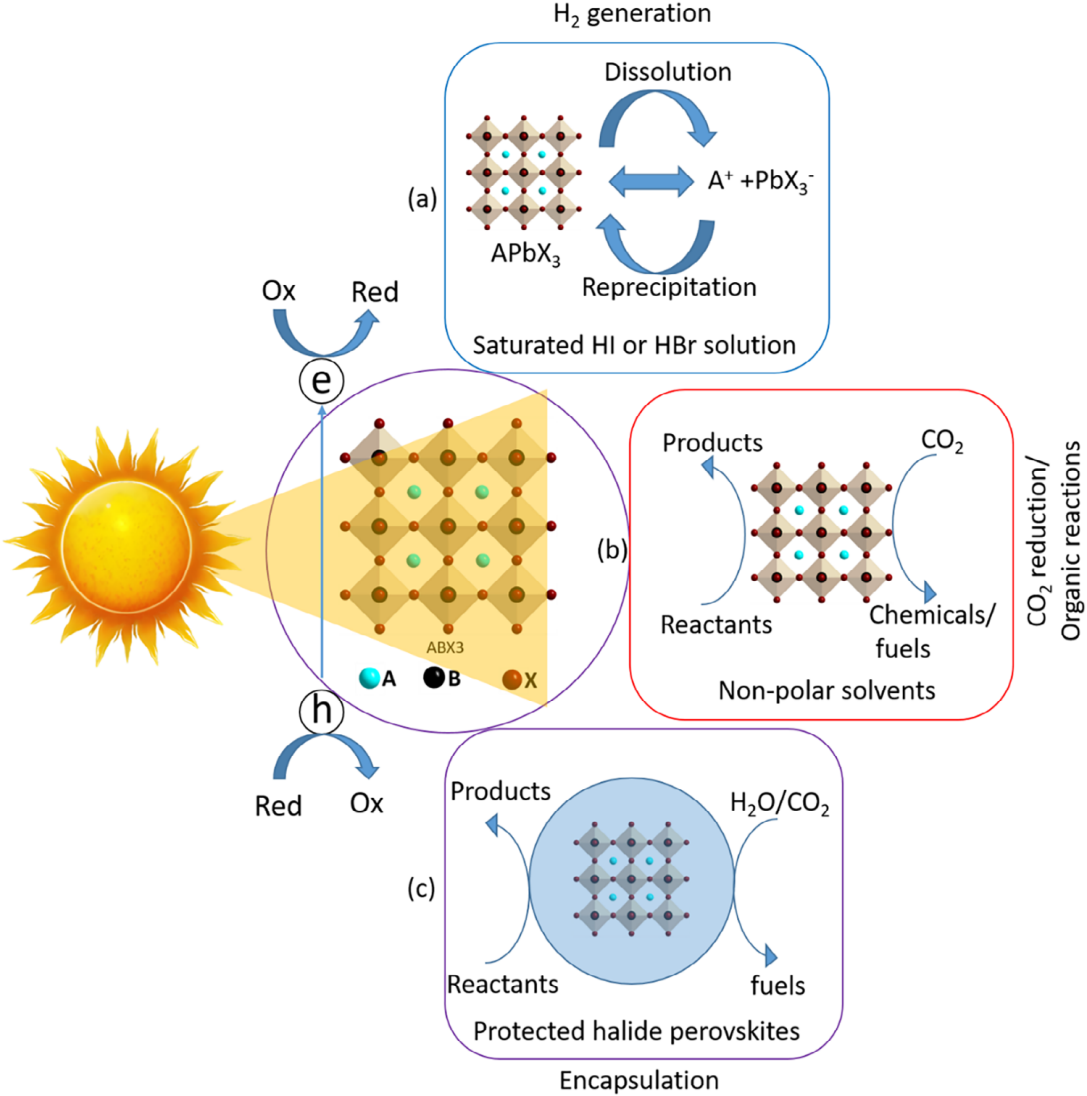
Strategies used to avoid polar solvent‐induced degradation in perovskite photocatalysis for H_2_ generation and CO_2_ reduction: a) Using saturated haloacid (HI or HBr) solutions that prevent the dissolution of perovskites by establishing a dynamic equilibrium between the dissolved ions (Cs^+^/MA^+^/FA^+^ and PbX_3_^−^) and perovskites. b) Using relatively less polar or non‐polar organic solvents to dissolve CO_2_ for its reduction. c) Encapsulation with bulky ligands or shell structures that prevent direct contact of colloidal or thin film perovskites with polar solvents. The materials used for encapsulation should not degrade in electrolyte solution but should transport photogenerated charges.

The reaction medium or solvent in which heterogeneous photocatalytic reactions are performed plays a crucial role in the efficiency and long‐term stability of catalysts.^[^ ^66^, ^67^, ^68^, ^69^ ^]^ In general, photocatalytic H_2_O splitting, CO_2_ reduction, and dye degradation reactions require water or polar solvents.^[^ ^17^, ^70^, ^71^, ^72^ ^]^ However, unlike traditional photocatalysts, such as oxides (e.g., TiO_2_) and metal chalcogenides (e.g., CdS), halide perovskites are prone to degradation in water due to the soft and ionic nature of these materials.^[^ ^40^, ^45^, ^46^, ^62^, ^64^, ^73^, ^74^, ^75^, ^76^, ^77^ ^]^ This section briefly discusses the most widely implemented strategies for performing perovskite photocatalysis by overcoming their degradation in polar solvents.^[^ ^46^, ^78^, ^79^, ^80^ ^]^ These methods include the use of hydrohalic acid solutions (e.g., HBr, HCl) instead of pure water for hydrogen evolution reactions,^[^ ^47^ ^]^ low‐polarity solvents for dissolution of CO_2_ for its reduction (e.g., ethyl acetate),^[^ ^81^ ^]^ encapsulated perovskites (thin films and colloids) that survive in aqueous solutions,^[^ ^39^, ^82^, ^83^, ^84^ ^]^ surface and ligand engineering of perovskites that offers the inherent stability in water, which are schematically illustrated in **Figure** **2**.

### 2.1 Saturated Hydrohalic Acid Solutions

An innovative approach that has been used to overcome the instability of MHPs in polar solvents is the use of saturated hydrohalic acids (HBr or HI) as a solvent medium that helps to establish a dynamic equilibrium between the disintegration of perovskites into ions (A^+^ and PbX_3_^−^) and their recrystallization, thereby enabling the photocatalytic transformation of HX into hydrogen (Figure 2a).^[^ ^47^, ^85^, ^86^, ^87^, ^88^ ^]^ Park and co‐workers first reported this approach to convert HI into H_2_ using MAPbI_3_ as the photocatalyst.^[^ ^47^ ^]^ The idea behind this approach is that the perovskites precipitate in a saturated solution as the concentration of ions exceeds the solubility limit, thus stabilizing the perovskite material. Therefore, the minimum concentration of perovskites required for a stable colloidal dispersion, i.e., a saturated perovskite, in HI solution can be obtained from solubility measurements. For MAPbI_3_, the solubility limit at 20 °C was found to be ≈0.645 mol L^−1^. In addition, the concentration of H^+^ and I^−^ ions was found to be crucial for the stabilization of the perovskite crystals in the saturated aqueous solution. This is because PbI_2_, which is less soluble in aqueous solution, dissolves in water in the form of PbI_3_^−^ or PbI_4_^2−^ at high iodide concentrations.^[^ ^47^ ^]^ On the other hand, the intermolecular hydrogen bonding between water molecules at highly acidic conditions can decrease the hydration of perovskites. Therefore, a high ionic strength along with a low iodide concentration ([I^−^] ≤ [H^+^]) and high acidic conditions are required for the long‐term stability of a perovskite phase. This (i.e., [I^−^] ≤ [H^+^]) can be achieved by the addition of HClO_4_ to a saturated HI solution.^[^ ^47^ ^]^ Under these optimum conditions, the MAPbI_3_ photocatalyst remains stable under continuous light irradiation for 160 h and splits HI into H_2_ with constant efficiency. It should be noted that the I^−^ of HI converts into I_3_^−^ during the photocatalytic reaction, and the solution turns brown, affecting light absorption by the MHP. To overcome this, H_3_PO_2_ was added as a selective reducing agent to reconvert I_3_^−^ into I^−^, enabling constant H_2_ production from HI (This is also briefly repeated in section 3: H_2_ evolution). This approach has been extensively used in perovskite photocatalysis for H_2_ production using different photocatalysts, including Pb‐based (e.g., MAPbBr_3_, CsPbBr_3_) and Pb‐free (e.g., A_2_^I^B^I^B^III^X_6_, MA_3_Bi_2_I_9_) materials.^[^ ^31^, ^40^, ^43^, ^44^, ^46^, ^85^, ^89^, ^90^, ^91^, ^92^, ^93^, ^94^ ^]^ Currently, perovskite photocatalysts are undergoing further chemical engineering (tuning halide composition and combining them with co‐catalysts such as TiO_2_, Pt, black phosphorus, and graphene oxide) to improve their stability and efficiency.^[^ ^87^, ^88^, ^90^, ^95^, ^96^, ^97^, ^98^, ^99^, ^100^, ^101^, ^102^, ^103^, ^104^ ^]^ Some examples are discussed in section 3 regarding their preparation and H_2_ evolution efficiency (µmol g^−1^ h^−1^).

### 2.2 Organic Solvents for CO_2_ Reduction and Organic Chemical Reactions

Another solution to the problem of perovskite instability in polar electrolytes would be to use lower‐polarity or non‐polar organic solvents to perform perovskite photocatalysis for CO_2_ reduction and organic chemical reactions (Figure 2b).^[^ ^29^, ^81^, ^105^, ^106^, ^107^, ^108^, ^109^ ^]^ Organic solvents do not dissociate ionic perovskite crystals as they do not coordinate well with the corresponding ions. For this reason, organic solvents have been commonly used as antisolvents in perovskite crystallization on a substrate and colloidal solution.^[^ ^29^, ^81^, ^105^, ^106^, ^107^, ^108^, ^109^ ^]^ In addition, ligand‐capped colloidal perovskite nanocrystals can be well dispersed in organic solvents, which makes them ideal candidates for photocatalytic CO_2_ reduction and organic chemical transformations.^[^ ^110^, ^111^ ^]^ However, the solubility of CO_2_ is also crucial in choosing the most appropriate solvent for efficient photocatalysis. In addition, one has to be cautious in the analysis of the reaction products of photocatalysis in organic solvents. This is because perovskite photoreduction of organic solvents can lead to CO production up to a rate of 1000 µmol g^−1 ^h^−1^. Such photoredox organic molecules can produce CO and CH_4_ at a much higher rate than in typical photocatalytic CO_2_ reduction reactions. There is a chance the reaction products could simply be due to the organic chemical transformation, rather than CO_2_ reduction. Therefore, control experiments, e.g., analysis of reaction products without CO_2_ in the reaction medium, are needed to avoid the overestimation of efficiencies.^[^ ^112^ ^]^ Furthermore, the photodecomposition of organic capping ligands can also lead to the production of CO without CO_2_ in the reaction medium. However, the process mainly occurs under UV light irradiation. Therefore, it is important to cut off UV light from the excitation source using a UV filter.^[^ ^113^ ^]^ Despite many available organic solvents, acetonitrile and ethyl acetate, which have relatively low polarity, have been the most widely used in perovskite photocatalysis for CO_2_ reduction.^[^ ^46^, ^111^ ^]^ In addition, a trace amount of water is also used as a hole scavenger and thus generates oxygen and hydrogen through a water‐splitting reaction that competes with CO_2_ reduction (Figure 2b). On the other hand, for organic chemical reactions, organic solvents, such as acetonitrile, dichloroethane, dichloromethane, toluene, chloroform, tetrahydrofuran, dichloroethane, dioxane, and ethanol have often been used depending on the required reaction conditions.^[^ ^29^, ^114^, ^115^, ^116^, ^117^, ^118^, ^119^, ^120^ ^]^ Over the years, both Pb‐based and Pb‐free perovskites (e.g., CsPbBr_3_, Cs_2_AgBiBr_6_, Cs_2_SnI_6_, Cs_3_Bi_2_I_9_) in combination with co‐catalysts, such as graphene oxide, graphene, carbon nitride, TiO_2_, MOFs, and Mxene, have been studied as photocatalysts for CO_2_ reduction.^[^ ^43^, ^46^, ^111^, ^121^, ^122^, ^123^, ^124^, ^125^, ^126^, ^127^, ^128^, ^129^ ^]^ Some of these are discussed in Section 4 on perovskite photocatalytic CO_2_ reduction. On the other hand, colloidal NCs of inorganic halide perovskites, especially, CsPbBr_3_, have been used in photocatalytic chemical transformations (See Section 5 about chemical transformations).^[^ ^29^, ^31^, ^130^ ^]^

### 2.3 Encapsulation of Perovskites

Physically separating the perovskite material from the dispersant/electrolyte is another effective way to prevent their degradation.^[^ ^53^, ^73^, ^131^, ^132^, ^133^ ^]^ This can be achieved through a core‐shell structure (for nanocrystals), or the use of a protective over‐layer in the case of thin films (Figure 2c).^[^ ^41^, ^42^, ^43^, ^80^, ^132^, ^134^, ^135^ ^]^ These protective layers can also prevent the degradation of perovskites by harsh conditions, such as heat and UV light, and provide long‐term stability.^[^ ^73^ ^]^ However, the shells must be transparent to light and allow charge carriers to pass through to reach their surface where light‐induced chemical transformations (water splitting, CO_2_ reduction, and chemical synthesis) occur.^[^ ^136^, ^137^ ^]^ In addition, the protective materials also act as co‐catalysts for enhanced charge separation.^[^ ^81^, ^137^ ^]^

Various inorganic coatings have been explored using techniques like atomic layer deposition (ALD) and solution processing to apply thin layers onto the perovskites with an organic buffer layer in between. Materials such as AlO_x_, zeolites, and glassy substances (including silicates and lead oxide) offer stability for up to 3–4 months against water but fail to address photocatalytic challenges due to their insulating properties.^[^ ^39^ ^]^ In contrast, inorganic semiconducting coatings of a few nanometers (1–10 nm) resolve this issue while facilitating charge‐carrier transport. For example, Li et al. reported coating CsPbBr_3_ with TiO_2_ using a titanium precursor followed by calcination at 300 °C (higher temperatures could lead to perovskite degradation). The resulting composites maintained stable charge transport properties for over three months. The composite exhibited reduced resistance for charge transport and thus led to enhanced photocurrent compared to pure CsPbBr_3_, as revealed by electrochemical impedance spectroscopy and photocurrent measurements, respectively.^[^ ^52^ ^]^ Similarly, Xu et al. coated CsPbBr_3_ with amorphous TiO_2_ and used it as a photocatalyst for CO_2_ reduction.^[^ ^83^ ^]^ In this case, charge consumption increased by 6.5 times, and CO_2_ adsorption improved significantly. Such coatings are of great interest to the catalysis community, offering a synergistic effect that enhances charge separation and reactant adsorption. Although these structures possess poor dispersibility in water and thus are not well suited for solution‐based photocatalysis.^[^ ^138^ ^]^ However, in the case of photoelectrochemical water‐splitting devices, the hole and electron transport layers, as well as the electrodes coated on the perovskite layers, also serve as protective layers.^[^ ^40^, ^131^, ^139^ ^]^ Further details on the use of encapsulated colloidal perovskite NCs and perovskite photoelectrochemical cells for water splitting, CO_2_ reduction, and chemical reactions are discussed in later sections.

### 2.4 Surface Engineering

**Figure 3:**
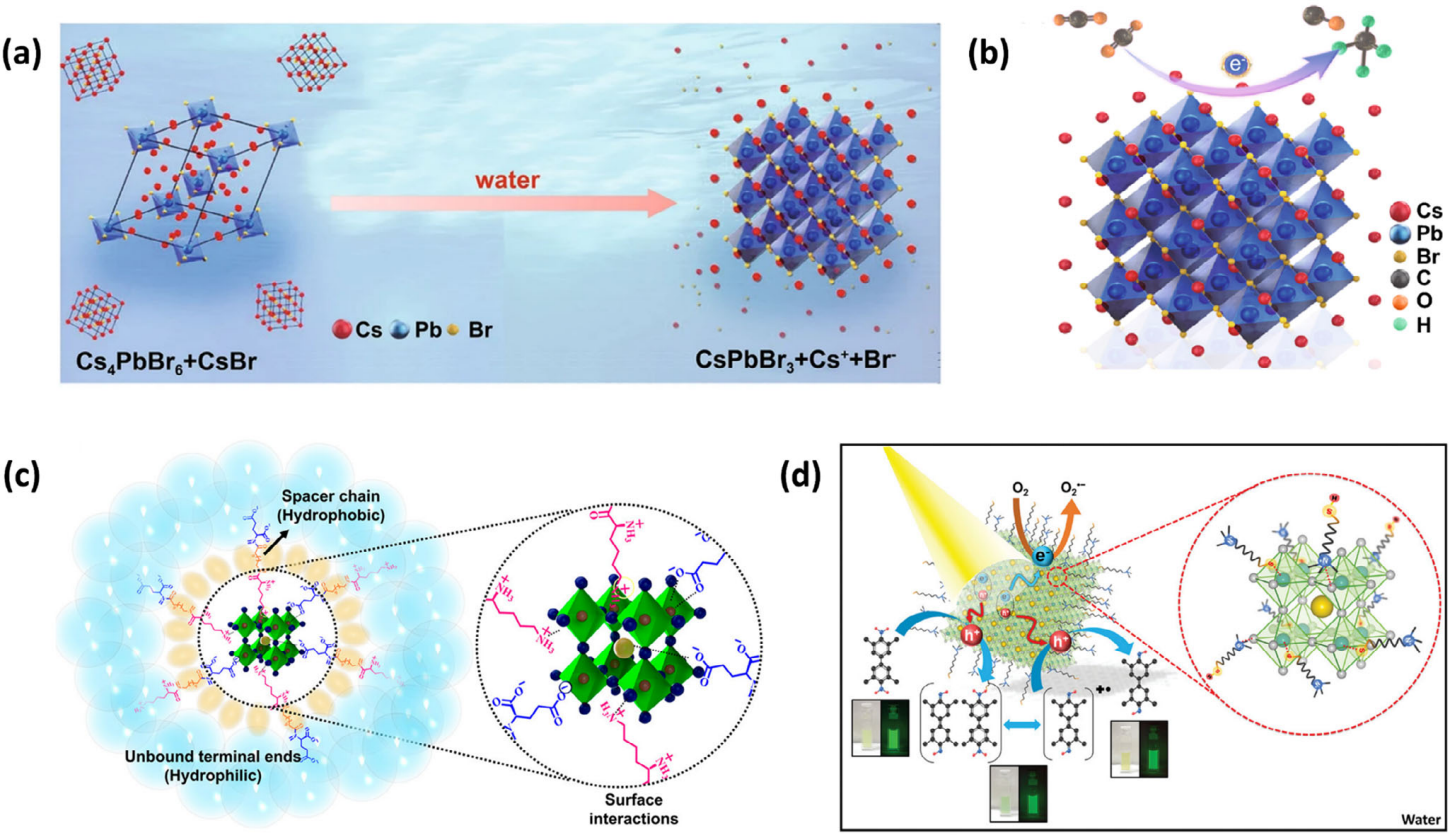
a) Schematic illustration of the phase transition from Cs_4_PbBr_6_ to CsPbBr_3_ in water. b) Illustration of CO_2_ reduction reaction using water‐dispersed CsPbBr_3_. Reproduced with permission.^[^ ^138^ ^]^ Copyright 2021, the Author(s). c,d) Schematic representation of NKE‐12 (c) (Reprinted with permission.^[^ ^42^ ^]^ Copyright 2023, American Chemical Society) and MUTAB (d) ligand anchored on perovskite surface and their interaction with water. Reproduced with permission.^[^ ^141^ ^]^ Copyright 2024, Wiley‐VCH.

Another strategy to maintain charge transfer properties with improved stability in polar solvents is surface engineering. This approach involves modifying the surface of perovskites to prevent polar molecules, like water, from interacting with the core structure. Using non‐stoichiometric precursors creates different intermediates, leading to the formation of various phases. An example of a core‐shell structure investigated is CsPbBr_3_ core with Cs_4_PbBr_6_ shell, doped with cobalt prepared while capping with hexafluoro butyl methacrylate. The structures were further employed for photocatalytic CO_2_ reduction in pure water.^[^ ^140^ ^]^ Chen et al. synthesized water‐dispersible CsPbBr_3_ nanocrystals (NCs) by utilizing a hot‐injection method under non‐stoichiometric conditions.^[^ ^138^ ^]^ Unlike encapsulated structures, these nanocrystals demonstrated remarkable water dispersibility and exceptional stability for over 200 days. The synthetic process was designed to create a Cs‐ and Br‐rich environment while maintaining low Pb conditions, resulting in mixed‐phase CsBr/Cs_4_PbBr_6_ structures. An optimized washing procedure further facilitated the formation of water‐dispersible CsPbBr_3_ NCs, schematically presented in **Figure** **3**a.^[^ ^11^ ^]^ These findings were attributed to the well‐passivated CsBr termination on the nanocrystals, which can potentially form an electrical double layer, acting as a protective barrier. When tested for catalytic applicability, these nanocrystals were successfully employed in electrochemical CO_2_ reduction in water (Figure 3b). These strategies have met almost all the essential requirements for photocatalysis in polar solvents, making them a promising area for further exploration.

### 2.5 Ligand Engineering

Catalysis requires more than just water‐stable hybrid perovskites. It also necessitates water‐dispersible perovskites with enhanced charge localization to catalytically active sites. Achieving this involves engineering ligands that can stabilize colloidal lead halide perovskite nanocrystals in water. To date, ligands with dual functionality offering both hydrophobicity and strong anchoring properties have been investigated to improve water stability. For example, Haung and colleagues successfully stabilized CsPbX_3_ (X = Br/I) nanocrystals in water for nearly 10 weeks by using polyhedral oligomeric silsesquioxane (POSS). POSS formed a cage‐like structure around the nanocrystals, with lead‐sulfur bonds providing stability.^[^ ^142^ ^]^ Additionally, metal stearates have been explored as ligands, where the stearate imparts hydrophobicity, leading to excellent water stability.^[^ ^143^ ^]^ However, despite these promising approaches, they have not been widely applied in photocatalysis due to challenges related to charge transport, surface accessibility, and water dispersibility.

**Figure 4:**
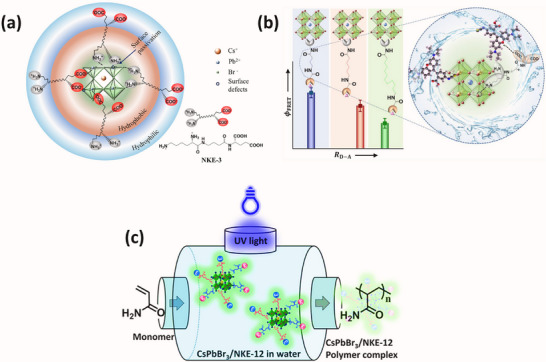
a) Schematic representing interaction strategy of short‐chain NKE‐3 ligands with CsPbBr_3_ surface and water, simultaneously. Reproduced with permission.^[^ ^54^ ^]^ Copyright 2023, American Chemical Society. b) Bar graph showing a qualitative trend of energy transfer efficiency with the variation of donor‐acceptor distance using NKE ligands. Reproduced with permission.^[^ ^144^ ^]^ Copyright 2023, American Chemical Society. c) Schematic showing photocatalytic polymerization of acrylamide in water catalyzed by NKE‐12‐stabilized perovskites. Reproduced with permission.^[^ ^53^ ^]^ Copyright 2024, American Chemical Society.

Recently, Rao and colleagues introduced a novel approach to stabilize CsPbBr_3_ nanocrystals (NCs) in aqueous environments using tailored bolaamphiphilic ligands. These ligands feature two polar functional groups connected by an organic spacer, enabling dual functionality. One polar end interacts directly with the perovskite surface, creating a strong anchoring effect, while the other end effectively localizes water molecules away from the perovskite surface. This dual action minimizes water‐induced degradation and enhances the stability of the NCs. As shown in **Figures** 3c and **4**a, these perovskite NCs dispersed uniformly in water and retained their photophysical properties for over 15 days.^[^ ^42^, ^54^, ^141^ ^]^ The ligands used were the amino acid derivatives NKE‐3 and NKE‐12, where the numbers 3 and 12 indicate the length of the organic spacer. This spacer length plays a crucial role in dictating the stability and functionality of the NCs. Building upon this strategy, the group further employed MUTAB as a ligand (Figure 3d), which leverages the strong lead‐sulfur (Pb‐S) bond for effective surface anchoring. The ammonium functional group at the opposite end of MUTAB serves to localize and organize water molecules away from the perovskite surface, further enhancing the NCs' aqueous stability. Remarkably, these MUTAB‐stabilized NCs maintained their photophysical integrity and excellent dispersibility in water for over 2 months.^[^ ^141^ ^]^ The incorporation of these ligands onto the NC surface was achieved using a post‐synthetic ligand exchange method, which ensured efficient surface coverage and robust ligand binding.

Beyond stability, the stabilized halide perovskites demonstrated their utility in energy and charge transfer applications. They efficiently transferred energy and charges to acceptor dyes, such as rhodamine and coumarin. Additionally, systematic variation of the spacer length (NKE‐3, −5, and −11) revealed an inverse relationship between spacer length and energy transfer efficiency, with shorter spacers showing better energy transfer performance (Figure 4b). This finding highlights the tunable nature of these ligands and their significant potential in photocatalysis, where precise energy and electron transfer processes are critical.^[^ ^144^ ^]^

Building on these foundational studies, subsequent research from the group confirmed the practical applicability of these systems in photocatalysis. The stabilized halide perovskites successfully catalyzed polymerization reactions and redox transformations in aqueous media.^[^ ^53^, ^141^ ^]^ As shown in Figures 3d and 4c, these reactions leveraged the robust stability and enhanced charge transfer properties imparted by the engineered ligands. This work represents a significant advancement in the development of halide perovskite materials for use in polar solvents, offering a pathway to simultaneously improve their stability and preserve their photocatalytic functionality.

The strategies described in these studies could serve as a blueprint for advancing the stability of halide perovskites in challenging environments, particularly in polar media, without compromising their desirable photophysical and photocatalytic properties. This strategy holds transformative potential for the future of halide perovskite‐based catalysis.

## 3 H_2_ Evolution

In the context of hydrogen evolution, we start with a discussion of the main technologies for hydrogen production using sunlight and outline the limiting factors associated with some of these processes both in terms of the technology itself (Section 3.1.), as well as from the viewpoint of thermodynamic and kinetic limitations of the underlying chemistry (Section 3.2.). Specifically, in the case of the latter, we include a discussion of the mechanistic intricacies of the oxygen evolution reaction (OER). This is often neglected in the context of lead‐halide perovskites. Granted, these materials are not a straightforward candidate for water oxidation due to moisture sensitivity. Nonetheless, it is often suggested or implied that MHPs can act as photocatalysts for oxygen evolution, whether in the context of OWS or performing gas‐phase CO_2_ reduction in the presence of water vapor. After discussion of the thermodynamic and kinetic aspects, we outline the metrics used to evaluate catalytic activity (Section 3.3). In Section 3.4., we review the literature relevant to MHP photocatalysts for hydrogen evolution. This is followed by a brief review of the application of these materials in the context of photoelectrochemical water splitting in Section 3.5.

### 3.1 Green Hydrogen Production

**Figure 5:**
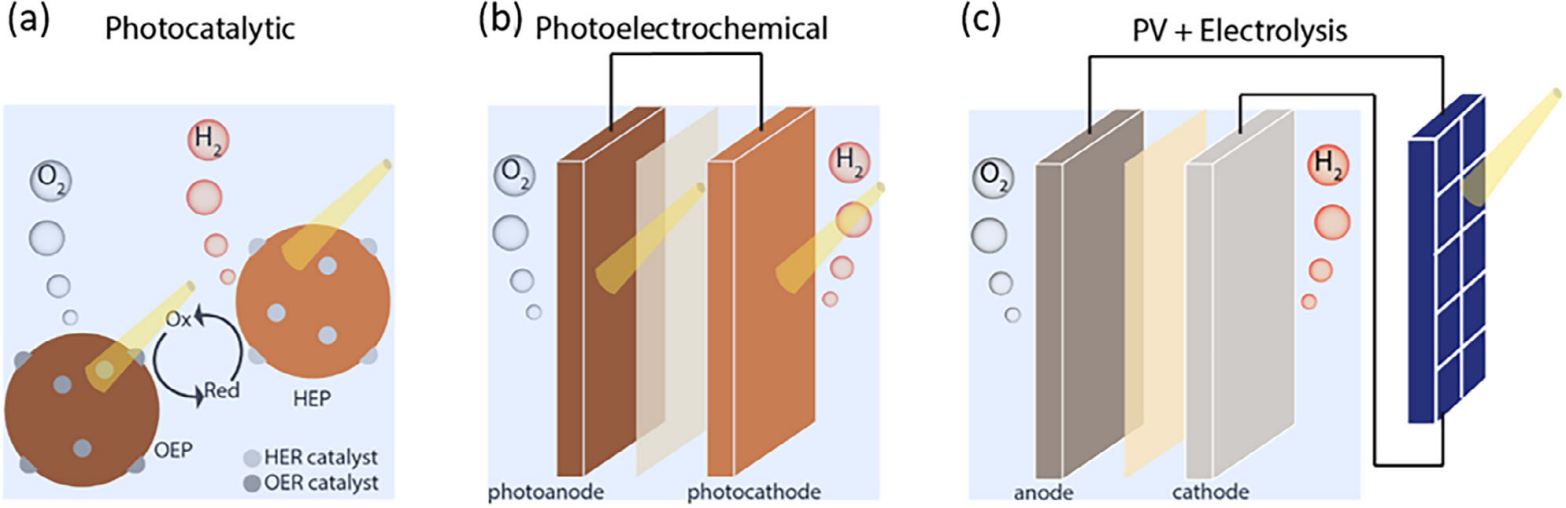
Diagram showing the three main technologies for generating hydrogen from sunlight: a) photocatalytic, b) photoelectrochemical, and c) photovoltaic‐electrolyzer systems. OEP and HEP in (a) correspond to the oxygen evolution photocatalyst and hydrogen evolution photocatalyst, respectively. The illustrations are representations of example systems of each technology, but other configurations are possible for each. For example, in the photocatalytic case represented here, the photogenerated electron on the HEP component reduces protons to make H_2_, while the photogenerated hole is consumed by a redox mediator to form the “Ox” species. Similarly, the photogenerated hole on the OEP oxidizes water, while the corresponding electron is consumed by the oxidized mediator to regenerate the “Red” species. This is referred to as a redox‐mediated Z‐scheme. Another possible device is a hybrid between PEC and PV‐EC, with one of the photoelectrodes in (b) relying on a catalyst‐coated PV cell, and the whole system being fully integrated and wireless. This is referred to as integrated PV‐PEC tandem or artificial leaf.^[^ ^154^, ^156^ ^]^

There are three device types that absorb solar energy to drive water or HI splitting.^[^ ^47^, ^145^ ^]^ These are, in order of increasing complexity: a particulate system, a photoelectrochemical cell (PEC), or a photovoltaic‐electrolyzer (PV‐EC) tandem system (**Figure** **5**). PV‐EC tandems have already surpassed the 10% solar‐to‐fuel efficiency threshold required for commercialization,^[^ ^146^ ^]^ and are the most mature technology, with a technology readiness level of 9.^[^ ^147^ ^]^ The particulate device is the simplest in many respects, requiring no wiring, multi‐component devices, or a corrosive electrolyte, making it the most promising in terms of yielding low cost green hydrogen.^[^ ^148^ ^]^ Nonetheless, there are significant hurdles yet to overcome for this to be viable on a large scale, namely system efficiency, stability, and safety in terms of gas separation in the context of OWS. As for the other two technologies, it is increasingly believed that traditional PECs can no longer compete against PV‐EC, a technology that has benefitted from the low cost of electricity produced by silicon PV cells.^[^ ^147^, ^149^, ^150^, ^151^ ^]^ In fact, the levelized cost of hydrogen is estimated at 6.22 and 8.43 $ kg_H2_^−1^ for a PV‐EC and a PEC system, respectively.^[^ ^150^ ^]^ Nonetheless, these costs remain significantly higher than that of grey hydrogen, which averaged 2.13 $ kg_H2_^−1^ in 2023,^[^ ^145^, ^149^, ^151^, ^152^ ^]^ as well as the US Department of Energy Hydrogen Shot target of 1 $ kg^−1^ H_2_ by 2031.^[^ ^153^ ^]^ This highlights the importance of ongoing research into particulate systems, as well as the promising PV‐PEC hybrids, also termed artificial leaves.^[^ ^154^, ^155^ ^]^ The latter offers the advantage of more compact devices compared to traditional PV‐EC. Furthermore, their potential for commercialization can benefit from the increased flexibility, weight reduction, and cheaper manufacturing, which are afforded by thin‐film PV technologies, such as perovskite solar cells.

### 3.2 Thermodynamics and Kinetics of Water and HI Splitting

Overall Water Splitting (OWS): The light‐driven splitting of water was first reported in 1972 by Fujishima and Honda using a photoelectrochemical cell with TiO_2_ as a photoanode and Pt as a cathode.^[^ ^24^ ^]^ This was followed by the use of particulate photocatalytic systems in 1980.^[^ ^157^ ^]^

**Figure 6:**
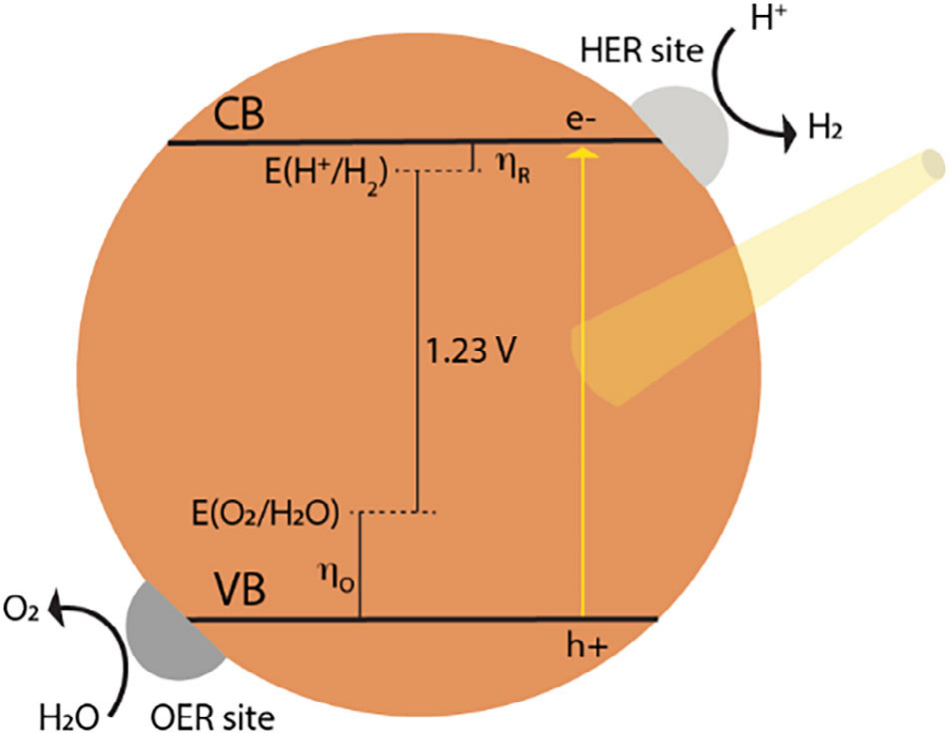
Schematic energy diagram of single‐component‐mediated photocatalytic water splitting. The diagram shows the overpotentials η_O_ and η_R_ for the oxidation and reduction half‐reactions, respectively; the water‐splitting redox potential (1.23 V); photoexcited charges; co‐catalyst active sites for HER and OER.

The major limitation to commercial‐scale green hydrogen production via water‐splitting is finding a system that combines high efficiency, stability, and low cost. The reaction is thermodynamically uphill and requires a standard Gibbs free energy (∆*G* ^0^) of 237.2 kJ mol^−1^, or 1.23 V per electron (Equations (1), (2), (3)). A semiconductor capable of OWS needs to have a bandgap of at least 1.23 eV, with its conduction band (CB) being shallower than the redox potential of the reduction half‐reaction, and its valence band (VB) deeper than the redox potential of the oxidation half‐reaction (**Figure** **6**).

(1) $$\begin{array}{ccc} \mathrm{Proton}\;\mathrm{reduction}: & & 2H_{3}O^{+}+2e^{-}\to H_{2}+2H_{2}O\\ & & E^{\theta }=0.00\;V\;\mathrm{versus}\;\mathrm{RHE}\;\mathrm{at}\;\mathrm{pH}=0 \end{array}$$

(2) $$\begin{array}{ccc} \mathrm{Water}\;\mathrm{oxidation}: & & 6H_{2}O\to O_{2}+4H_{3}O^{+}+4e^{-}\\ & & E^{\theta }=1.23\;\;V\;\mathrm{versus}\;\mathrm{RHE}\;\mathrm{at}\;\mathrm{pH}=0 \end{array}$$

(3) $$\begin{array}{ccc} \mathrm{Water}\;\mathrm{splitting}: & & 2H_{2}O\to 2H_{2}+O_{2}\\ & & E^{\theta }=1.23\;V\;\mathrm{versus}\;\mathrm{RHE}\;\mathrm{at}\;\mathrm{pH}=0 \end{array}$$

The steps involved in OWS are: i) light absorption by the semiconductor, ii) charge separation in the light harvester, iii) migration of the electron (hole) to the reduction (oxidation) site, where iv) charge transfer to H^+^ (H_2_O) occurs, followed by v) H_2_ (O_2_) desorption and collection. Each step presents limitations that depend on the photocatalyst (PC) in question, as well as on the device employed.

Proton reduction is a relatively simple two‐electron process that proceeds via a single adsorbed intermediate and is suggested to proceed via one of two possible mechanisms: Volmer‐Tafel or Volmer‐Heyrovsky.^[^ ^158^ ^]^ The reactions involved in the latter are illustrated in Equations (4) and (5).

(4) $$2H^{+}+2e^{-}\to H_{\mathrm{ads}}+H^{+}+e^{-}$$

(5) $$H_{\mathrm{ads}}+H^{+}+e^{-}\to H_{2}$$

This is in stark contrast with the sluggish, four‐electron transfer, oxygen evolution reaction (OER).^[^ ^159^ ^]^ The conventional mechanism for OER is the adsorbate evolution mechanism (AEM). Herein, discussion of the mechanism for OER will focus on the example of the extensively studied metal‐oxide catalysts, which include perovskite oxides. AEM occurs via three intermediates: OH*, O*, and OOH*.^[^ ^160^, ^161^ ^]^ These occur at active metal ion sites, and the proposed mechanism involved is shown in Equations ((6), (7), (8), (9)).

(6) $$\mathrm{Step}\;1:\;2H_{2}O\to OH^{*}+H^{+}+e^{-}+H_{2}O$$

(7) $$\mathrm{Step}\;2:\;OH^{*}+H^{+}+e^{-}+H_{2}O\to O^{*}+2H^{+}+2e^{-}+H_{2}O$$

(8) $$\mathrm{Step}\;3:\;O^{*}+2H^{+}+2e^{-}+H_{2}O\to \mathrm{OO}H^{*}+3H^{+}+3e^{-}$$

(9) $$\mathrm{Step}\;4:\;\mathrm{OO}H^{*}+3H^{+}+3e^{-}\to O_{2}+4H^{+}+4e^{-}$$

The activation energy barrier for each step varies depending on the material, and the largest barrier defines the potential determining step (note that this may be different from the rate‐determining step).^[^ ^162^, ^163^ ^]^ This results in the need for an overpotential (η) to be supplied beyond the thermodynamic requirement of 1.23 eV to drive OWS. In fact, the band gap of the light‐harvesting semiconductor required for OWS is around 1.9–2.3 eV when taking into account thermodynamic losses (0.3–0.5 eV) and the required overpotential (0.4–0.6 eV).^[^ ^164^ ^]^

**Figure 7:**
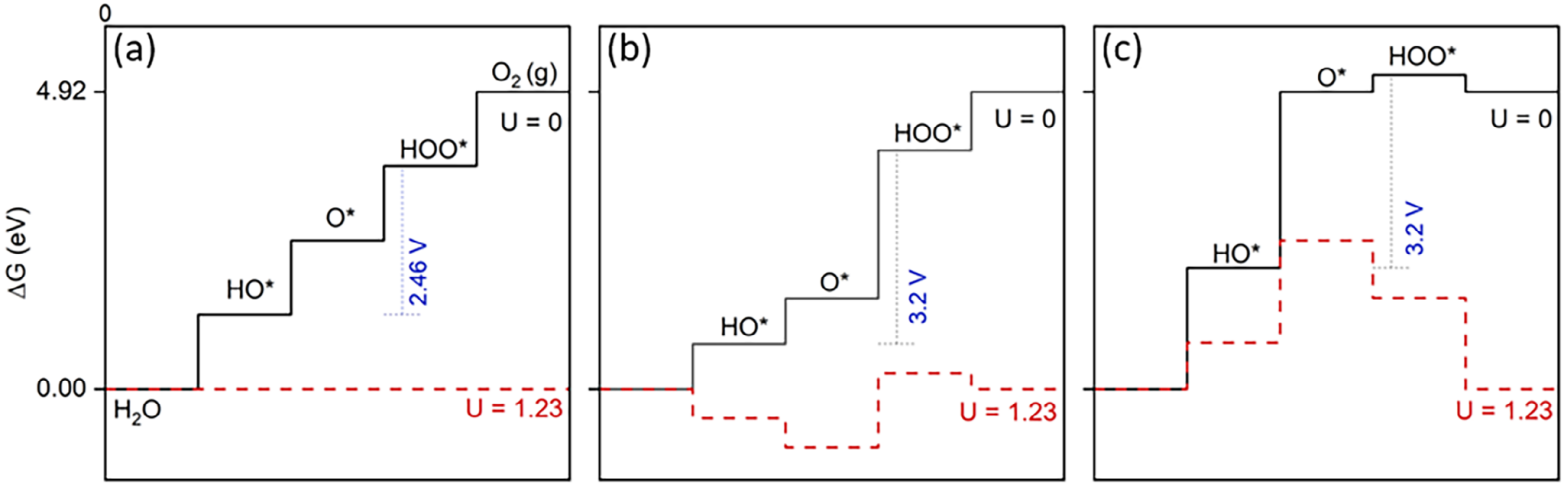
The free energy diagram for three types of metal oxide‐based OER catalysts modeled according to ref. [162]. a) The (non‐existent) ideal catalyst, where thermoneutrality is achieved at an applied potential U = 1.23 V (dashed red curve); b) a catalyst which binds the intermediates too strongly, making oxidation of O* (step 3) potential‐determining; c) a catalyst which binds the intermediates weakly, making oxidation of OH* (step 2) potential determining. In (b) and (c), U has to be significantly larger than 1.23 V to make all steps exothermic (U > 1.23 V not represented above for simplicity). The dashed blue lines and values in blue represent the difference between the binding energies of HOO* and HO*.

**Figure 8:**
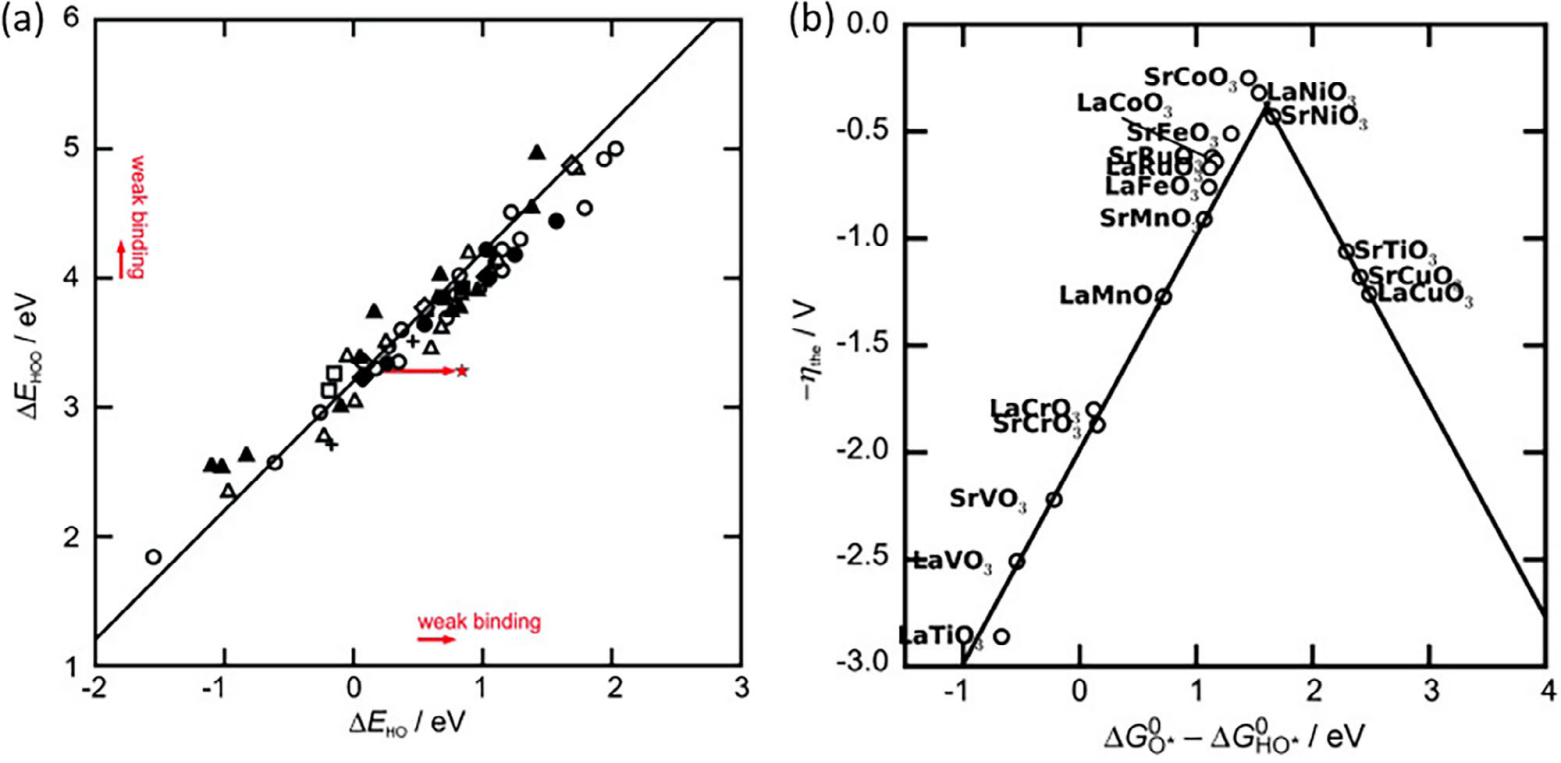
a) Adsorption energy ∆E of OOH* intermediate versus that of OH*, showing a linear scaling relation with an intercept of 3.2 V for a range of metal‐oxide catalysts. b) Volcano plot of the theoretical overpotential as a function of the free energy of the OH* oxidation (step 2) for a range of oxide perovskites. Both figures are reproduced with permission.^[^ ^162^ ^]^ Copyright 2011, Wiley‐VCH.

This is clearly illustrated in the energy diagram in **Figure** **7**, which shows that based on the equilibrium potential, a reaction free‐energy equivalent to +1.23 eV is expected for each of the four OER steps at no applied bias (U = 0 V). It follows that the ideal catalyst would mean that all four reactions have a free‐energy of 0 kJ mol^−1^ at an applied potential of U = 1.23 V (i.e., all steps are thermoneutral), and that at U > 1.23 V, the reactions are exothermic. However, this is never observed, since a potential‐determining step always occurs, such that a significant overpotential is required (U >> 1.23 V) to render all steps exothermic. The reason for this is the scaling relation that has been observed between the binding energies of OH* and OOH* on a wide range of oxide catalysts, as shown in **Figure** **8**a,^[^ ^162^ ^]^ and which is caused by the similar binding of both intermediates, on the same active site.^[^ ^162^, ^163^ ^]^

As a result, a constant free energy difference of 3.2 eV occurs between the two reactions (between steps 2 and 4), as shown in Figure 6, which translates to a minimum overpotential of ≈0.4 eV when we take into account the ideal free energy difference of 2.46 V ([3.2–2.46 V]/2). It thus follows that the difference ∆*G*_Oads_ – Δ*G*_OOHads_ determines the overpotential, i.e., whether step 2 or 3 is the potential‐determining step.^[^ ^162^, ^163^ ^]^ Reaction 4 is never potential‐determining in this model.^[^ ^162^, ^163^ ^]^ Given the above, ∆*G*_Oads_ – Δ*G*_OOHads_ is used as a descriptor to compare the activity of different OER catalysts in a volcano plot, with higher activity being synonymous with lower overpotential (Figure 8b). The minimum overpotential requirement means the apex of the volcano occurs around *η* = 0.4 V.

In the case of the hydrogen evolution reaction (HER), the descriptor is the adsorption energy of the only intermediate involved, i.e., H_ads_. To maximize activity, H_ads_ needs to interact sufficiently strongly with the catalyst for the second step to occur, but not too strongly in order to minimize the back reaction. Pt, at the apex of the volcano, is known to be the most efficient catalyst for HER due to optimal adsorption energy (ΔG_Hads_ ≈ 0).^[^ ^90^, ^165^ ^]^ Given the above considerations regarding thermodynamic and kinetic requirements for the two half‐reactions involved in OWS, photocatalytic H_2_ production has largely relied on sacrificial oxidations to replace the demanding water oxidation half‐reaction.

HI Splitting: The process of splitting hydrohalic acids is thermodynamically and kinetically easier than OWS, as it involves a Gibbs free‐energy change of 103.3 kJ mol^−1^ (less than half that of OWS), and is a two‐electron process, as shown in Equations ((10), (11), (12)).

(10) $$\begin{array}{ccc} \mathrm{Proton}\;\mathrm{reduction}: & & 2H_{3}O^{+}+2e^{-}\to H_{2}+2H_{2}O\\ & & E^{\theta }=0.00\;V\;\mathrm{versus}\;\mathrm{RHE}\;\mathrm{at}\;\mathrm{pH}=0 \end{array}$$

(11) $$\begin{array}{ccc} \mathrm{Iodide}\;\mathrm{oxidation}: & & 3I^{-}\to I_{3}^{-}+2e^{-}\\ & & E^{\theta }=0.53\;V\;\mathrm{versus}\;\mathrm{RHE}\;\mathrm{at}\;\mathrm{pH}=0 \end{array}$$

(12) $$\begin{array}{ccc} \mathrm{HI}\;\mathrm{splitting}: & & 2H_{3}O^{+}+3I^{-}\to H_{2}+I_{3}^{-}+2H_{2}O\\ & & E^{\theta }=0.53\;V\;\mathrm{versus}\;\mathrm{RHE}\;\mathrm{at}\;\mathrm{pH}=0 \end{array}$$

The overpotential is negligible for iodide oxidation, such that photocatalysts displaying smaller bandgaps (i.e., harvest sunlight more efficiently) can be used. As mentioned above, H_3_PO_2_ is added to circumvent browning of the medium with increasing I_3_^−^ formation, as shown in Equation (13).

(13) $$\begin{array}{ccc} {I_{3}}^{-}\;\mathrm{reduction}: & & H_{3}PO_{2}+2I_{3}^{-}+6H_{2}O\to \\ & & H_{3}PO_{4}+6I^{-}+4H_{3}O^{+} \end{array}$$

Control experiments show that H_3_PO_2_ is not oxidized in the absence of I_3_^−^, confirming that photogenerated holes do not react with H_3_PO_2_ directly.^[^ ^47^, ^166^ ^]^ The reaction in Equation (13) regenerates I^−^ and also replenishes the consumed aqueous H^+^ (i.e., H_3_O^+^) at the same ratio at which they react in Equation (12). This highlights the role of H_3_PO_2_ in not just preventing parasitic I_3_^−^ absorption but also in maintaining the delicate balance required between [I^−^] and [H^+^] to suppress side reactions.^[^ ^47^ ^]^

### 3.3 Evaluating Photocatalytic Activity

Strategies to boost the performance of photocatalysts include relying on low‐bandgap semiconductors to harness abundant visible‐light photons; in fact, traditional wide‐bandgap semiconductors used to drive overall water splitting can only harness UV radiation and thus are restricted to a solar‐to‐fuel energy conversion efficiency of around 2% at a quantum efficiency of 100% (i.e., assuming that all photogenerated charge‐carriers react).^[^ ^25^ ^]^ Other important strategies to enhance activity include improving charge separation efficiency and charge transport, extending charge lifetime, and reducing the barrier for charge transfer at active sites.

**Figure 9:**
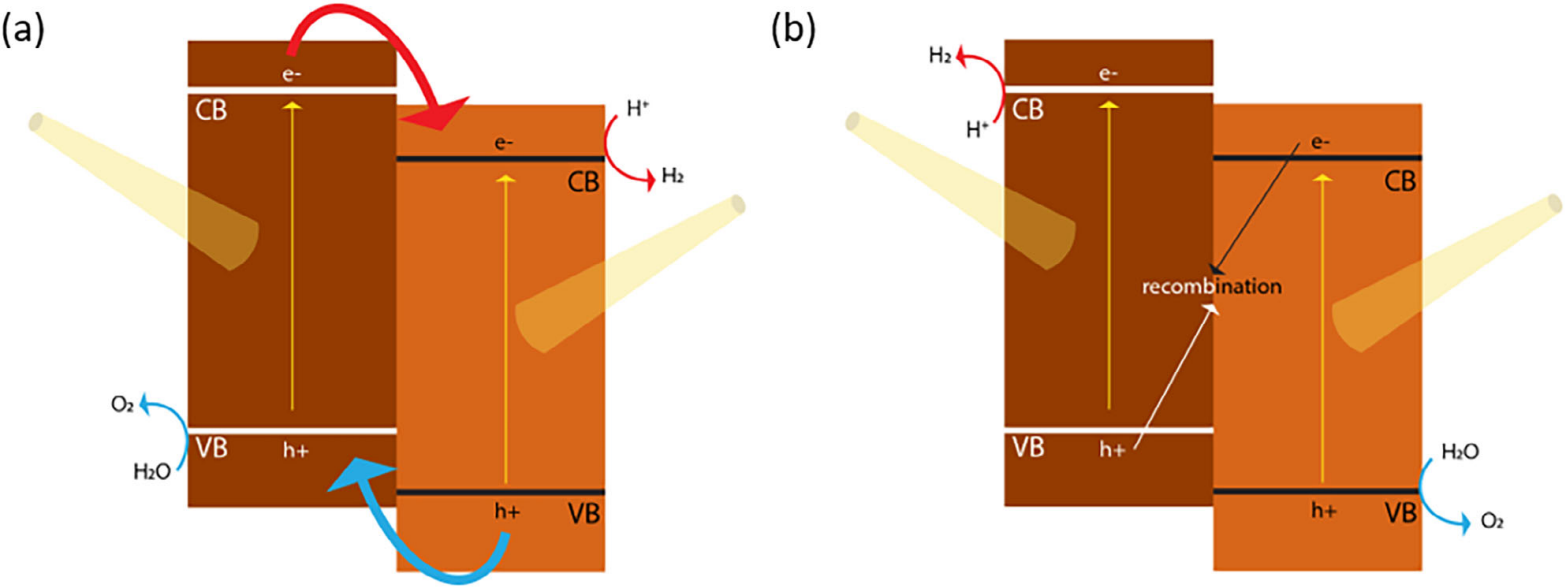
Schematic diagrams of a) type‐II heterojunction and b) Z‐scheme heterojunction. Note that the Z‐scheme represented here illustrates a *direct* Z‐scheme. In alternative configurations, consumption of the low‐energy electron and low‐energy hole occurs via a redox mediator, as illustrated in Figure 5a.

Traditional OWS photocatalysts consist of transition metal oxides given their stability and suitable band edge positions relative to the redox potentials for OWS. While the addition of metal cocatalysts can be used to reduce the energetic barrier, the reliance on a single component to drive both HER and OER, while also harnessing a significant proportion of visible light photons, is very challenging. Hence the use of two‐component systems in binary heterojunctions, which allows the use of smaller bandgap semiconductors to drive OER and HER separately. In a type‐II heterojunction (**Figure** **9**a), the energetic offsets between CB edge (VB edge) and HER (OER) potential at the interface contribute to enhancing charge separation efficiency and reducing recombination. Z‐schemes are difficult to achieve, but compared to type‐II heterojunctions, they present smaller energetic losses since recombination occurs between the lower‐energy electron and hole, leaving the higher‐energy photogenerated charges free for driving the desired redox reactions (Figure 9b).

In terms of evaluating a system's performance, the rate of H_2_ evolution (µmol h^−1^) is a useful parameter within a single study, but cannot be used to draw meaningful comparisons between different studies. Firstly, it does not take into account variables related to the experimental set‐up, like photocatalyst loading, incident energy, and reactor surface area. Normalizing to mass (µmol g^−1^ h^−1^) is not meaningful either as the relationship between activity and mass will depend on the experimental setup, and may be limited at higher concentrations by the stability of the dispersion.^[^ ^167^, ^168^ ^]^ As a result, apparent quantum yield (AQY) is a more reliable wavelength‐specific metric to assess efficiency and compare across systems (Equation 14). The term “apparent” arises from the fact that it depends on the number of incident photons, rather than the number of photons absorbed.^[^ ^167^ ^]^

(14) $$\mathrm{AQY}\left(\%\right)=\frac{\mathrm{number}\;\mathrm{of}\;\mathrm{reacted}\;\mathrm{electrons}}{\mathrm{number}\;\mathrm{of}\;\mathrm{incident}\;\mathrm{photons}}\times 100\%$$

The solar‐to‐fuel (STF) conversion efficiency provides an estimate for the ratio between energy output and energy input. In the case of HER, it is referred to as the STH (solar‐to‐hydrogen) conversion efficiency(Equation 15).

(15) $$\mathrm{STH}=\frac{\mathrm{evolved}\;H_{2}\left(\mathrm{mol}\right)\times N_{A}\times 2\times E_{\mathrm{redox}}\left(\mathrm{eV}\right)\times e}{P_{\mathrm{sol}}\left(W.{\mathrm{cm}}^{-2}\right)\times \mathrm{area}\left({\mathrm{cm}}^{2}\right)\times \mathrm{time}\left(s\right)}\times 100\%$$

where *N*_A_ is Avogadro's number, *E*_red/ox_ indicates the redox potential of the reaction in question (whether water splitting or HI splitting), *e* is the elementary charge, and *P*_sol_ is the power density of incident light.

### 3.4 Halide Perovskites for Photocatalytic H_2_ Evolution

**Figure 10:**
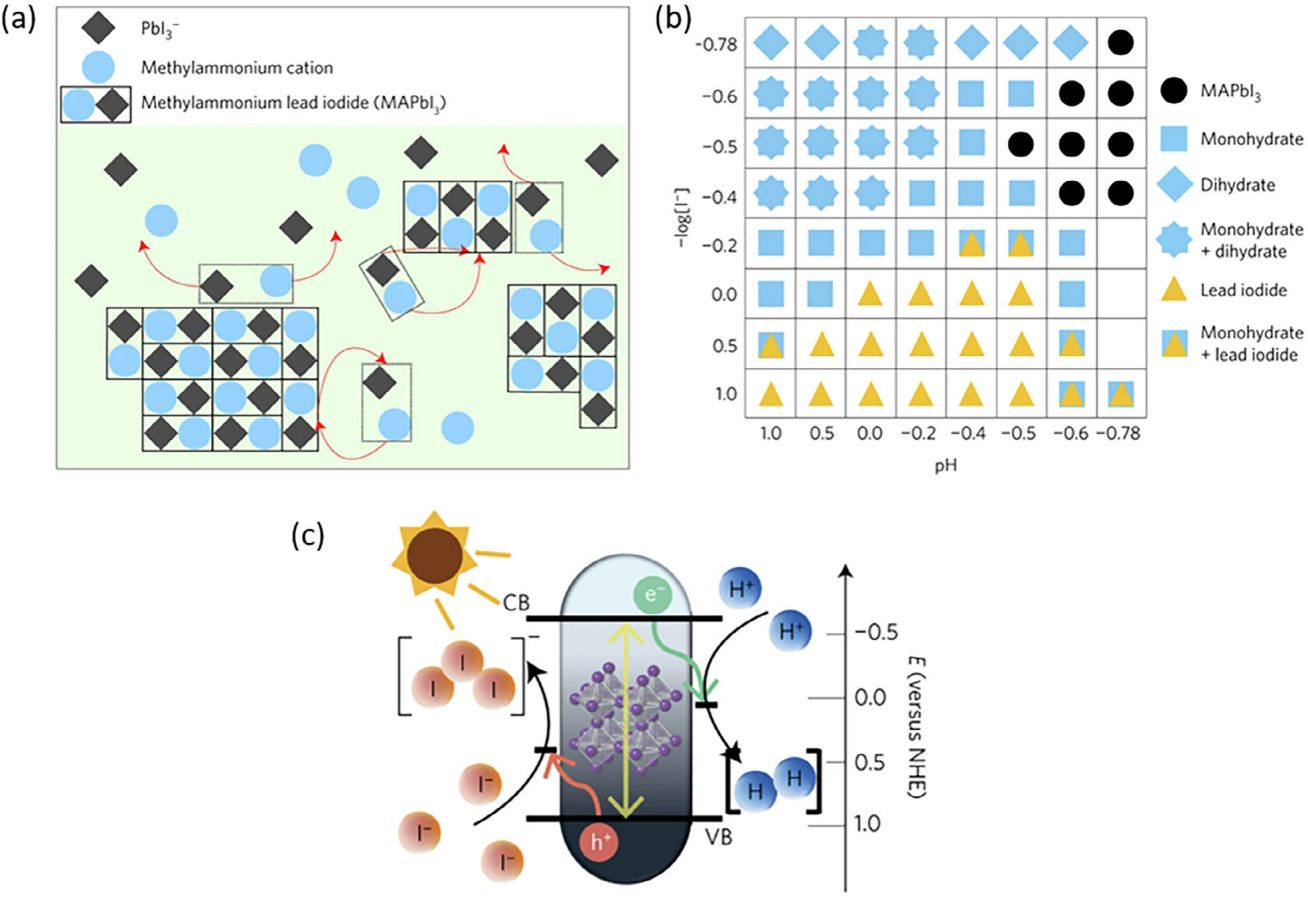
a) Schematic illustrating the dynamic equilibrium between dissolution and reprecipitation of MAPI in HI solution. b) Phase map showing the range of concentrations of I^−^ and H^+^ within which MAPI is stable in HI solution. c) Schematic energy band diagram illustrating H_2_ evolution via HI splitting on MAPI. a–c) Reproduced with permission.^[^ ^47^ ^]^ Copyright 2017, Springer Nature.

As discussed in the introduction, lead‐halide perovskites display tunable bandgaps with strong light absorption in the visible region, as opposed to many of their wider‐bandgap oxide counterparts. However, their instability in the presence of moisture makes their employment as PCs for OWS counterintuitive. In fact, as will be discussed further below, they are thus far mostly employed in the context of splitting hydrohalic acids. Under such conditions, the integrity of the perovskite photocatalyst is maintained by the dynamic equilibrium established between dissolution and reprecipitation (**Figure** **10**a).^[^ ^47^ ^]^

Strategies to stabilize MHPs towards moisture in the field of photovoltaics involve the use of external encapsulation of the device as a whole.^[^ ^169^ ^]^ This cannot be directly applied in the context of perovskite‐based photoelectrodes in PEC devices, since the encapsulants need to be conductive to allow charge transport to the surface of the electrode.^[^ ^154^ ^]^ Other methods involve composition tuning or structural modifications (3D to 2D or ligand‐stabilized QDs).^[^ ^170^ ^]^ However, moisture stability is a significant challenge in photocatalysis, as water molecules need to come in close contact with the surface of perovskite particles in the context of photocatalytic water splitting.

In the next two sections, we review the progress in lead‐halid perovskites (LHP) and lead‐free halide perovskites as photocatalysts for HER, with data from the literature summarized in **Table** **1**.

**Table 1:** Summary of reported photocatalytic hydrogen evolution systems using halide perovskites.

#	Photocatalyst	Reaction	Cocatalyst [wt%]	Light source	Medium	Performance	Measured stability [h]	Refs.
1	MAPbI_3_/Pt	HI splitting	1.7 wt% Pt	Solar simulator, λ ≥ 475 nm, 100 mW cm^−2^	aq. HI + H_3_PO_2_	11.4 µmol h^−1^ (200 mg), STH = 0.81%	160	[47]
	MAPbI_3_ (solvent anneal)					6 µmol h^−1^		
	MAPbI_3_					2 µmol h^−1^		
2	MAPbI_3_/rGO	HI splitting	3.4 wt% rGO	300 W Xe lamp, λ ≥ 425 nm, 120 mW cm^−2^	aq. HI + H_3_PO_2_	93.9 µmol h^−1^ (100 mg), AQY = 1.5% at 450 nm	200	[99]
	MAPbI_3_/Pt		1.9 wt% Pt			4 µmol h^−1^		
	MAPbI_3_					1.4 µmol h^−1^		
3	Pt/TiO2/MAPbI3	HI splitting	0.75 wt% Pt	300 W Xe lamp, λ ≥ 425 nm, 200 mW cm^−2^	aq. HI + H_3_PO_2_	72.8 µmol h^−1^ (10 mg), AQY = 70% at 420 nm, STH = 0.86%	12	[95]
	Pt/Ta_2_O_5_/MAPbI3		0.75 wt% Pt			39.9 µmol h^−1^		
	MAPbI_3_/Pt		0.5 wt% Pt			0.93 µmol h^−1^		
	MAPbI_3_					0.47 µmol h^−1^		
4	MAPbBr_3‐x_I_x_/Pt	HBr splitting	1.2 wt% Pt	Xe lamp, λ ≥ 420 nm, 100 mW cm^−2^	aq. HI/HBr + H_3_PO_2_	651.2 µmol h^−1^ (250 mg), AQY = 8.10% at 450 nm, STH = 1.05%	30	[171]
	MAPbBr_3‐x_I_x_					255.3 µmol h^−1^		
	MAPbBr_3_/Pt		1.2 wt% Pt			8.4 µmol h^−1^		
	MAPbBr_3_					2.8 µmol h^−1^		
5	CsPbBr_3‐x_I_x_/Pt	HBr splitting	1.13 wt% Pt	Xe lamp, λ ≥ 420 nm, 120 mW cm^−2^	aq. HBr + H_3_PO_2_	224 µmol h^−1^ (200 mg), AQY = 2.15% at 450 nm	50	[172]
	CsPbBr_3‐x_I_x_					23.6 µmol h^−1^		
	CsPbBr_3_					negligible (≈0 µmol h^−1^)		
6	MAPbI_3_/Ni_3_C‐15%	HI splitting		Xe lamp, λ ≥ 420 nm, 100 mW cm^−2^	aq. HI + H_3_PO_2_	118 µmol h^−1^ (50 mg), AQY = 16% at 450 nm	200	[173]
	MAPbI_3_/Pt					26.7 µmol h ^−1^		
	MAPbI_3_					2.15 µmol h^−1^		
7	Pt/Ta_2_O_5_/MAPbBr_3_/ PEDOT:PSS	HBr splitting	0.75 wt% Pt	Xe lamp, λ ≥ 420 nm, 150 mW cm^−2^	aq. HBr + H_3_PO_2_	105 µmol h^−1^ (150 mg), AQY = 16.4% at 420 nm	4	[174]
	Pt/Ta_2_O_5_/MAPbBr_3_		0.75 wt% Pt			6 µmol h^−1^		
	Pt/MAPbBr_3_		0.75 wt% Pt			2 µmol h^−1^		
8	Pt/CsPbI_3_/pCN	HER	3.9 wt% Pt	300 W Xe lamp, λ ≥ 420 nm, 120 mW cm^−2^	TEOA + aq. H_2_PtCl_6_	10 µmol h^−1^ (10 mg)	16	[175]
9	MAPb(I_1‐x_Br_x_)_3_ (x = 0.1)	HI splitting	n/a	300 W Xe lamp, λ ≥ 420 nm, 100 mW cm^−2^	aq. HI/HBr + H_3_PO_2_	29.4 µmol h^−1^ (20 mg), STH = 1.42%	252	[85]
	MAPbI_3_					0.74 µmol h^−1^		
10	Pt/TiO_2_/CsPbBr_3_ QDs‐9.9wt%	sacrificial WS	1 wt% Pt	300 W Xe lamp, λ ≥ 420 nm	H_2_O/MeOH vapor	4.05 µmol h^−1^ (30 mg)	160	[176]
11	MAPbI_3_ (piezophotocatalytic)	HI splitting		500 W tungsten‐halogen lamp, 100 mW cm^−2^	aq. HI	23.3 µmol h^−1^ (50 mg)	15	[177]
	MAPbI_3_ (piezocatalytic)					2.21 µmol h^−1^		
	MAPbI_3_ (photocatalytic)					3.42 µmol h^−1^		
12	MAPbI_3_/CoP	HI splitting	20 wt% CoP (theoretical)	150 W Xe lamp, λ ≥ 420 nm	aq. HI + H_3_PO_2_	1.96 µmol h^−1^ (2.5 mg MAPbI_3_)	3	[98]
	MAPbI_3_					0.24 µmol h^−1^		
13	PMA_2_PbI_4_/Pt (labelled PMPI)	HI splitting	2 wt% Pt	300 W Xe lamp, λ ≥ 420 nm, 200 mW cm^−2^	aq. PMAI + H_3_PO_2_	333 µmol h^−1^ (150 mg), STH = 1.57%	16	[178]
	PMA_2_PbI_4_					17 µmol h^−1^		
14	MAPbI_3_/BP	HI splitting	1.2% BP 2D nanoflakes	300 W Xe lamp, λ ≥ 420 nm	aq. HI + H_3_PO_2_	112.3 µmol h^−1^ (30 mg), AQY = 23.2% at 420 nm, STH = 0.93%	200	[97]
	MAPbI_3_/bulk‐BP		5% bulk BP			17.4 µmol h^−1^		
	MAPbI_3_/Pt					5.76 µmol h^−1^		
	MAPbI_3_					1.05 µmol h^−1^		
15	ML‐MoS_2_/MAPbI_3_‐MCs (intercalated)	HI splitting		300 W Xe lamp, λ ≥ 420 nm	aq. HI + H_3_PO_2_	1360 µmol h^−1^ (100 mg), AQY = 11.6% at 450 nm, STH = 1.09%	208	[166]
	ML‐MoS_2_/MAPbI_3_‐MCs (physical mixture)					97.2 µmol h^−1^		
	bulk‐MoS_2_/MAPbI_3_‐MCs					57.4 µmol h^−1^		
	MAPbI_3_‐MCs/Pt		1.88 wt% Pt			57.5 µmol h^−1^		
	MAPbI_3_‐MCs					6 µmol h^−1^		
16	FAPbBr_3‐x_I_x_/PtSA (single‐atom Pt)	HI splitting	1.8 wt% (ICP‐OES value)	300 W Xe lamp, AM1.5G, 100 mW cm^−2^	aq. HI + H_3_PO_2_	682.6 µmol h^−1^ (100 mg), AQY = 33.4% at 530 nm, STH = 4.5%	30	[90]
	FAPbBr_3‐x_I_x_		n/a			39.8 µmol h^−1^		
17	Cs_2_AgBiBr_6_/N‐C‐140	HBr splitting	n/a	300 W Xe lamp, λ ≥ 420 nm, 100 mW cm^−2^	aq. HBr + H_3_PO_2_	3.8 µmol h^−1^ (50 mg), AQY = 0.59% at 420 nm	24	[179]
	Cs_2_AgBiBr_6_		n/a			0.2 µmol h^−1^		
18	MA_3_Bi_2_I_9_/Pt	HI splitting	1.9 wt% Pt	300 W Xe lamp, λ ≥ 400 nm	aq. HI + H_3_PO_2_	6.76 µmol h^−1^ (40 mg), STH = 0.48%	70	[102]
	MA_3_Bi_2_I_9_		n/a			0.49 µmol h^−1^		
19	Cs_2_AgBiBr_6_/rGO	HBr splitting	2.5 wt% rGO	300 W Xe lamp, λ ≥ 420 nm	aq. HBr + H_3_PO_2_	9.78 µmol h^−1^ (200 mg), AQY = 0.16% at 450 nm	120	[180]
	Cs_2_AgBiBr_6_/Pt		2.5 wt% Pt			0.19 µmol h^−1^		
	Cs_2_AgBiBr_6_		n/a			0.12 µmol h^−1^		
20	Cs_3_Bi_2x_Sb_2–2x_I_9_/Pt (denoted CBSI‐0.3)	HI splitting	missing SI	AM1.5G, 100 mW cm^−2^	aq. HI	92.6 µmol h^−1^ (100 mg), AQY = 1.206% at 420 nm, STH = 0.32%	50	[181]
	Cs_3_Bi_2x_Sb_2–2x_I_9_		n/a			4.2 µmol h^−1^		
21	DMASnI_3_	OWS	n/a	300 W Xe lamp	DI water	0.64 µmol h^−1^ (200 mg)	16	[182]
22	33wt%‐DMASnBr_3_@g‐C_3_N_4_/Pt	sacrificial WS	3 wt% Pt	1500 W Xe lamp, λ = 300–800 nm, 50 mW cm^−2^	H_2_O + 10% (v/v) TEOA	36.3 µmol h^−1^ (21 mg), AQY = 6.6% at 300–800 nm	6	[183]
	33wt%‐DMASnBr_3_@g‐C_3_N_4_	sacrificial WS	n/a		H_2_O + 10% (v/v) TEOA	0.39 µmol h^−1^		
	33wt%‐DMASnBr_3_@g‐C_3_N_4_/Pt	OWS	3 wt% Pt		H_2_O	0.29 µmol h^−1^		
	DMASnBr_3_/Pt	sacrificial WS	3 wt% Pt		H_2_O + 10% (v/v) TEOA	0.13 µmol h^−1^		
23	Cs_2_AgBiBr_6_‐10/g‐C_3_N_4_	HBr splitting	9.1 wt% g‐C_3_N_4_	300 W Xe lamp, λ ≥ 420 nm	aq. HBr + H_3_PO_2_	3 µmol h^−1^ (50 mg)	42	[184]
	Cs_2_AgBiBr_6_		n/a			1.2 µmol h^−1^		
24	MA_3_Bi_2_I_9_/DMA_3_BiI_6_ (BBP‐5)	HI splitting	n/a	300 W Xe lamp, λ ≥ 420 nm	aq. HI + H_3_PO_2_	19.82 µmol h^−1^ (100 mg)	100	[185]
	MA_3_Bi_2_I_9_ (BBP‐0)					1.32 µmol h^−1^		
	DMA_3_BiI_6_ (BBP‐10)					2.48 µmol h^−1^		
25	Cs_2_SnI_6_/PtSA	HI splitting	0.12 wt% Pt	300 W Xe lamp, λ ≥ 420 nm	aq. HI + H_3_PO_2_	4.3 µmol h^−1^ (10 mg), TOF = 70.6 h^−1^	180	[186]
	Cs_2_SnI_6_/PtNP		3.88 wt% Pt			1.45 µmol h^−1^		
	Cs_2_SnI_6_					0.25 µmol h^−1^		
26	2.5wt%‐Cs_3_Bi_2_Br_9_/g‐C_3_N_4_/Pt	sacrificial WS	3 wt% Pt	1500 W Xe lamp, λ = 300–800 nm, 50 mW cm^−2^	H_2_O + 10% (v/v) TEOA	22.05 µmol h^−1^ (21 mg)	6	[187]
	Cs_3_Bi_2_Br_9_//Pt		3 wt% Pt			0.46 µmol h^−1^		
27	Cs_2_Pt_0.05_Sn_0.95_Cl_6_/Pt	sacrificial WS	5 wt% Pt	300 W Xe lamp, λ ≥ 420 nm	H_2_O + 10% (v/v) TEOA	0.85 µmol h^−1^ (50 mg)	4	[188]
28	5wt%‐PEA_2_SnBr_4_/g‐C3N4/Pt	sacrificial WS	3 wt% Pt	1500 Xe lamp, 50 mW cm^−2^	H_2_O + 10% (v/v) TEOA	33.9 µmol h^−1^ (21 mg)	6	[189]
	PEA_2_SnBr_4_/g‐C3N4		3 wt% Pt			0.084 µmol h^−1^		
29	[(CH_3_)_2_NH_2_]_3_[BiI_6_]/Pt	HI splitting	1 wt% Pt	425 nm LED	aq. HI + H_3_PO_2_	23.3 µmol h^−1^ (500 mg)	100	[190]
	[(CH_3_)_2_NH_2_]_3_[BiI_6_]					1.9 µmol h^−1^		
30	PMA_2_PbI_4_/MoS_2_	HI splitting	2.5 wt% MoS_2_	300 W Xe lamp, 100 mW cm^−2^	aq. HI	368.3 µmol h^−1^, AQY = 14.26% at 500 nm, STH = 2.31%	25	[191]
31	PMA_2_PbI_4_/MoS_2_−WO_3_/RuO_x_	OWS	2.5 wt% MoS_2_	300 W Xe lamp, 100 mW cm^−2^	aq. HI	154.5 µmol h^−1^, STH = 2.07%	25	[191]

#### 3.4.1 Lead Halide Perovskites

Herein, we start with the first example of LHP reported for HI splitting in 2017. We then review the reported works focusing on boosting activity and mitigating the reliance on precious metals. Works are categorized based on the dominant strategy: compositional engineering, dimensionality, heterojunction composites, and multi‐catalytic devices. Where overlap occurs between two or more strategies, priority is given to the one that allows interesting comparisons between different studies. HER rates (µmol h^−1^) constitute meaningful comparisons within the same study only.

The first successful report of MHPs for photocatalytic H_2_ generation was published in 2017.^[^ ^47^ ^]^ By employing a saturated solution of MAPbI_3_ in hydriodic acid, the authors reported a 0.44% STH efficiency, which reached 0.81% with the incorporation of 1.7 wt% Pt as cocatalyst. To stabilize the MAPbI_3_ powder in solution, precise control of the concentration of I^−^ and H^+^ ions was required, with the perovskite lattice being stable at [I^−^] ≤ [H^+^] when [H^+^] ≤ 3.16 mol L^−1^ (Figure 10). This led to stable H_2_ evolution for up to 160 h. The catalytic activity was optimized by annealing under a polar solvent atmosphere to increase crystallinity for more efficient charge transport and reduced non‐radiative recombination, which led to a 2‐to‐3‐fold increase in H_2_ evolved depending on the solvent used.

Since this report, there have been various attempts to boost the efficiency of photocatalytic hydrogen evolution by A) composition engineering (including co‐catalyst decoration); B) varying the dimensionality; and C) employing perovskite/semiconductor composites. Some studies have also explored the effect of D) combining photocatalysis with piezocatalysis or electrocatalysis.

##### Compositional Engineering

X‐Site Tuning: Tuning the composition of lead‐halide perovskite involves employing (i) mixed halides (X‐site tuning), and (ii) replacing the organic cation with an inorganic equivalent (A‐site tuning). As most efficient systems incorporate Pt as a co‐catalyst, we also review in this sub‐section alternative co‐catalysts.

Various studies investigated the effect of mixed halide compositions, either by doping the bulk of the perovskite or by inducing a gradient in composition. Two such reports made use of light‐induced ion exchange to establish a gradient of iodide ions across MAPbBr_3‐x_I_x_ and CsPbBr_3‐x_I_x_.^[^ ^171^, ^172^ ^]^ This induced a gradual narrowing of the bandgap from the deeper (bromide‐rich) regions towards the surface (iodide‐rich) regions, thus accumulating photogenerated electrons at the surface. The MA‐based system yielded better HER rates: the bandgap‐funnel structure in MAPbBr_3‐x_I_x_ presented a 91‐fold improvement in rate relative to MAPbBr_3_ (from 2.8 to 255.3 µmol h^−1^), compared to a maximal 24‐fold improvement in the cesium‐based system (from ≈0 to 23.6 µmol h^−1^). The addition of 1.2 wt% Pt more than doubled the rate for MAPbBr_3‐x_I_x_, reaching 651.2 µmol h^−1^, with an AQY of 8.1% at 450 nm and a STH efficiency of 1.05%.^[^ ^171^ ^]^ It is worth noting that the authors report STH efficiency based on the HER rate in the first hour only. However, their data shows an induction period at the beginning of the experiment before the rate stabilizes. We re‐calculate STH using the hourly rate based on the total H_2_ evolved by the end of the experiment (651.2 µmol h^−1^, as opposed to 161.5 µmol h^−1^ in the first hour). This yields an STH of 4.2% which we will refer to further below to compare with a similar system in section B, where STH is reported based on the same calculation we made.

Another study investigated the same MA‐based mixed‐halide perovskite, but by doping the bulk of MAPbI_3_ with bromide, rather than inducing a gradient.^[^ ^85^ ^]^ The authors see a more modest 40‐fold improvement in HER rate (0.74 to 29.4 µmol h^−1^), with an STH of 1.42% without the use of Pt. They attribute the improved performance to a combination of longer‐lived charges, more efficient charge separation, and better transport. Furthermore, they suggest that the incorporation of the smaller Br ions induces the formation of Pb defect sites due to the breaking of the X‐Pb‐X linkage, which does not occur in pure MAPI. These defect sites are proposed to act as H_2_ evolution sites within an amine‐assisted mechanism where MA^+^ cations are the source of “H” for H_2_ formation. However, this seems unlikely as it would lead to degradation of the perovskite, and it is mechanistically unclear how MA^+^ cations can transfer “H” to form PbH^−^, as suggested by the authors.

Co‐Catalyst Modifications: In one report, the authors rely on the same bandgap‐funnel structure discussed above, but their main aim was to enhance the utilization of photogenerated charge‐carriers by optimizing Pt‐loading.^[^ ^90^ ^]^ They suggest that the limited HER rates in Pt‐loaded MHP photocatalysts thus far are likely due to poor utilization of photogenerated charges. Two reasons are proposed: i) the dynamic dissolution/reprecipitation equilibrium existing at the surface of the perovskite particles dispersed in saturated HX solutions prevents the formation of static Pt active sites, and ii) the size of Pt clusters. Larger clusters are undesirable as they constitute a poor ratio of available active sites to the amount of Pt used, meaning a lower efficiency of extracting photogenerated electrons. With the aim of enhancing charge extraction, they rely on FAPbBr_3‐x_I_x_ as a photocatalyst given FA‐based MHP's superior carrier lifetime and diffusion compared to its MA‐based counterpart, with the bandgap‐funnel structure acting as an additional driving force to drive electrons to the surface. However, while the authors report an STH of 4.5% and compare it to the 1.05% found for the MAPbBr_3‐x_I_x_/Pt system mentioned above, the two systems perform similarly since, as explained above, the re‐calculated STH for MAPbBr_3‐x_I_x_/Pt yields 4.2%. Furthermore, despite evidence of photodeposited single‐atom Pt (PtSA) on FAPbBr_3‐x_I_x_, the 1.8wt% loading determined from ICP‐OES remains quite high and is comparable to other reports relying on regular Pt nanoparticles.^[^ ^47^, ^186^ ^]^ Nonetheless, thanks to the efficient trapping of photogenerated electrons and the significant reduction in the activation barrier for H_2_ evolution, a 17‐fold improvement in rate was achieved by deposition of Pt, leading to a HER rate of 682.6 µmol h^−1^. We estimate that this corresponds to a turnover frequency (TOF) of 24.7 h^−1^, assuming that the totality of the 1.8wt% Pt used does indeed correspond to single‐atom active sites. Despite the ability of Pt to efficiently catalyze hydrogen evolution, its use on a commercial scale is prohibitive given its cost and scarcity. With this in mind, there are various examples of employing co‐catalysts based on abundant transition metals.

TiO_2_ is an established metal oxide catalyst for OWS. In 2018, the effect of combining TiO_2_ and MAPI for HI splitting was explored, relying on 33 wt% Pt‐TiO_2_ (with a Pt loading of 0.75wt% on TiO_2_).^[^ ^95^ ^]^ they observe a HER rate of 72.8 µmol h^−1^, an 89‐fold improvement on using only Pt‐decorated MAPbI_3_. The authors use a higher light intensity of 2 suns. While TiO_2_ is a photocatalyst in its own right, it requires UV photons to excite electrons across its bandgap. Since only visible light is involved in this study, it is worth highlighting that its role is purely co‐catalytic in this instance. Depositing the Pt co‐catalyst on TiO_2_ rather than the perovskite leads to better activity as it avoids the instability associated with Pt sites on MAPbI_3_ given the dissolution/reprecipitation equilibrium at the surface of MAPI crystals. Following optimization of (i) the amount of Pt/TiO_2_, (ii) Pt‐loading, and (iii) composite fabrication with a preheating treatment, they suggest the enhanced performance is due to a dynamic heterojunction which allows efficient electron transfer from MAPI to TiO_2_, where proton reduction is catalyzed on stable Pt active sites. They apply the same rationale to composites of either Pt‐Ta_2_O_2_ or Pt‐Nb_2_O_5_ with MAPI. The former yields more modest rates, which is assigned to a reduction in surface area, while the latter shows negligible activity due to unsuitable band positions. The same group then extended this idea. They employed a hole‐extraction layer to try to boost the performance of their Pt/Ta_2_O_5_‐MAPbBr_3_ system.^[^ ^174^ ^]^ They tested various hole‐transporting materials (HTM) in the composite Pt/Ta_2_O_5_‐MAPbBr_3_‐HTM, and PEDOT: PSS yielded the best performance under 1.5 sun (105 µmol H_2_ h^−1^) due to enhanced hole transfer from MAPbBr_3_ and a superior ability to catalyze Br^−^ oxidation. While this represented a 17‐fold improvement on the photocatalyst without PEDOT: PSS, stability was limited by aggregation of the hole‐transport material.

A photocatalyst based on nickel carbide as a co‐catalyst displayed a four‐fold improvement in HER rate compared to the Pt‐loaded MAPI equivalent.^[^ ^173^ ^]^ The reported MAPbI_3_/Ni_3_C‐15% yielded a HER rate of 118 µmol h^−1^, four times that of its Pt‐loaded counterpart and 55‐fold higher than MAPbI_3_. The Ni_3_C‐decorated perovskite was obtained via electrostatic self‐assembly and was stable over 200 h of operation. The origin of the improved performance is attributed to efficient electron transfer onto Ni_3_C, supported by the observed reduction in the lifetime of charge decay. Furthermore, they find that the composite can separate charges more efficiently and improve the kinetics of proton reduction, as evidenced by a reduction in the overpotential required compared to pure MAPI.

Drawing inspiration from electrocatalysis, one study investigated the effect of decorating the perovskite with cobalt phosphide (CoP).^[^ ^98^ ^]^ At a theoretical loading of 20wt% CoP, an 8‐fold improvement in rate was observed in the performance of MAPbI_3_ nanocrystals (0.24 to 1.96 µmol h^−1^), with an AQY of 1.5% at 450 nm. The CoP was photodeposited onto the perovskite, and the loading was optimized for enhanced catalytic activity while mitigating recombination induced by excessive loading. Similar to the effect of other co‐catalysts, it was found that CoP decoration led to enhanced charge transfer from the perovskite to the co‐catalyst, as evidenced by enhanced PL quenching and reduced charge lifetime. Another strategy employed in this study was reducing the size of the perovskite crystals to increase the exposed surface area. An increase in the HER rate is observed over the first 21 hours, which the authors attribute to enhanced crystallinity of MAPI and more CoP being photodeposited onto the surface.

##### Dimensionality

The dimensionality can be tuned on two levels: form factor and material structural dimensionality.^[^ ^192^, ^193^ ^]^ In 2D perovskites (discussed here in terms of material‐dimensionality), the layer of hydrophobic R groups can act as a barrier against water ingress, making these materials potential candidates for water splitting. Some limitations of 2D perovskites compared to their 3D counterpart are stronger exciton binding energies and wider bandgaps. The latter is not necessarily a limitation in the context of photocatalysis given the large overpotentials required to drive some of the desired reactions.

One study employed a bulky organic cation to induce the formation of a 2D perovskite, namely PMA_2_PbI_4_, (abbreviated PMPI).^[^ ^178^ ^]^ It was expected that the bulky cation should confer stability in water for H_2_O splitting. Since this was not the case, the authors used PMPI to drive HI splitting, and observed a HER rate of 333 µmol h^−1^ with 2 wt% Pt (up from 17 µmol h^−1^ without Pt). The authors stabilize the perovskite by tuning the concentration of PMAI salt in aqueous solution. In contrast to the more established scenario where a saturated solution of the perovskite is used to stabilize the photocatalyst powder,^[^ ^47^, ^174^ ^]^ a negligible amount of PMA_2_PbI_4_ is dissolved, likely an effect of the more hydrophobic PMA^+^ compared to MA^+^. Furthermore, they observe that increasing the hydrophobicity further by increasing the length of the organic cation leads to a significant drop in performance, which they attribute to less efficient charge separation/migration and slower charge transfer to Pt. Another study focused on reducing form factor dimensionality by studying 0D quantum dots (QD).^[^ ^176^ ^]^ CsPbBr_3_ QDs were employed in a composite with Pt‐loaded TiO_2_ to promote proton reduction and vapor‐phase methanol oxidation. It was found that control of the ligand density was critical. They observe longer induction times at higher ligand densities, but a more stable perovskite structure. The proposed rationale is based on irradiation‐induced ligand degradation/removal in the first minutes/hours of photocatalysis, until a suitable density is reached to allow electron transfer onto TiO_2_, while also protecting against moisture. A loading of 9.86 wt% perovskite yielded the best rate (4.05 µmol h^−1^), with 1 wt% Pt photodeposited on TiO_2_ and a stability of 160 h (compared with ≤23 h at lower initial ligand densities).

##### Perovskite/Semiconductor Heterojunction Composites

Rather than catalyzing both half‐reactions on the same semiconductor, binary heterojunction composites can be used to enhance charge‐carrier seperation, as discussed earlier. Below we review the MHP/semiconductor composites employed for HX splitting.

##### Inorganic Semiconductors

The metal chalcogenide MoS_2_ is another example of a well‐known metallic electrocatalyst,^[^ ^194^ ^]^ that can also display semiconducting properties with a visible‐light response.^[^ ^195^ ^]^ Its behavior is dictated by the formation of the metallic 1T phase or the semiconducting 2H phase. One study investigated the composite ML‐MoS_2_/LHP‐MC based on monolayers (ML) of MoS_2_ intercalated into microcrystals (MCs) of various LHPs.^[^ ^166^ ^]^ Of the four LHPs tested, MAPbI_3_ was by far the best‐performing. The authors confirm the presence of both 1T and 2H phases of MoS_2_ using Raman spectroscopy and XPS. It is worth noting that while the reported absorption spectrum of ML‐ MoS_2_ suggests a dominant metallic behavior, we classify this system under perovskite/semiconductor heterojunction composites rather than perovskite/co‐catalyst composites, given the known visible‐light response of ML‐MoS_2_.^[^ ^196^ ^]^ The resultant type‐II heterojunction successfully enhanced charge separation efficiency. In fact, they observe a clear spatial separation of electrons and holes on ML‐MoS_2_ and MAPbI_3_‐MC, respectively. They also find that careful control of the thickness of ML‐ MoS_2_ and size of MAPbI_3_‐MC is required to reach the optimal rate of 1360 H_2_ µmol h^−1^ and an STH efficiency of 1.09%. Simple mixing of ML‐MoS_2_ and MAPbI_3_‐MC leads to a 93% drop in the HER rate, while employing bulk‐MoS_2_ leads to a 96% drop. Furthermore, an intermediate size of the MAPbI_3_‐MC is needed, indicating the required balance between a high surface area but also an appropriate density of intercalated ML‐MoS_2_.^[^ ^185^ ^]^

Also relying on a perovskite/MoS_2_ composite, a recent study offers an elegant solution to the additional costs associated with relying on H_3_PO_2_ as a sacrificial agent in HX splitting by replacing it with a water‐oxidation catalyst, namely WO_3_ decorated with RuO_x_. This not only serves as a more useful oxidation, but also regenerates I^−^ from I_3_^−^. Furthermore, the reaction medium is much less corrosive than the traditional HI‐reliant systems (pH = 2.9 vs −0.5).^[^ ^47^, ^191^ ^]^ OWS proceeds at a promising STH of 2.07%.^[^ ^191^ ^]^

In another approach, a 1.2 wt% black phosphorous (BP)/MAPbI_3_ system showed a 19‐fold improvement in rate compared to Pt‐loaded MAPI (112.3 vs 5.76 µmol h^−1^).^[^ ^97^ ^]^ This required the use of 2D few‐layer BP, as opposed to bulk BP which yielded a rate of only 17.4 µmol h^−1^ due to a more limited number of active sites. A promising AQY of 23.2% at 420 nm was reported, the highest so far amongst Pt‐free LHP composites. Despite the type‐I heterojunction formed, with the associated risk of recombination on the smaller‐bandgap material, photoelectrochemical and spectroscopic characterization indicate efficient charge separation and transfer at the semiconductor interface, as well as improved transport.

##### Organic Semiconductors

Initial efforts following the first 2017 report of MAPI for hydroiodic acid splitting were directed towards perovskite/organic heterojunction composites. One study published in 2018 showed a 23‐fold increase in the activity of MAPbI_3_ by replacing 1.9 wt% Pt with 3.4 wt% reduced graphene oxide (rGO), going from 4.0 to 93.9 µmol h^−1^.^[^ ^99^ ^]^ The system was stable during 200 h of testing and achieved an AQY of 1.5% at 450 nm. The improved performance was attributed to enhanced charge separation via favorable electron transfer from perovskite to rGO, thus reducing recombination.^[^ ^180^ ^]^

##### Photocatalysis + Piezocatalysis/Electrocatalysis

While the focus so far has been on purely photocatalytic redox processes, two studies combine photocatalysis with a second technology to boost activity.

An interesting study used the piezoelectric properties of MAPbI_3_ to induce a built‐in electric field for enhanced charge separation and accumulation of charges in opposite directions, favoring a spatial separation of oxidation and reduction processes.^[^ ^177^ ^]^ The piezophotocatalytic effect led to an HER rate of 23.3 µmol h^−1^, a 7‐fold and a 11‐fold enhancement in rate compared to photocatalytic and piezocatalytic, respectively. The study relied on ultrasonic vibration as a source of mechanical stress, and it was observed that control of sonication power was critical to avoid conversion from the piezoactive tetragonal MAPbI_3_ to the piezoinactive orthorhombic structure. Even if catalytic activity is improved, such a system seems difficult to scale‐up; larger‐scale ultrasonication is more difficult to control. Furthermore, the authors rely on measuring the concentration of I_3_^−^ in solution to quantify evolved H_2_. However, the absorption of I_3_^−^, especially at higher concentrations, limits the absorption of MAPI in the visible range.^[^ ^47^ ^]^

**Figure 11:**
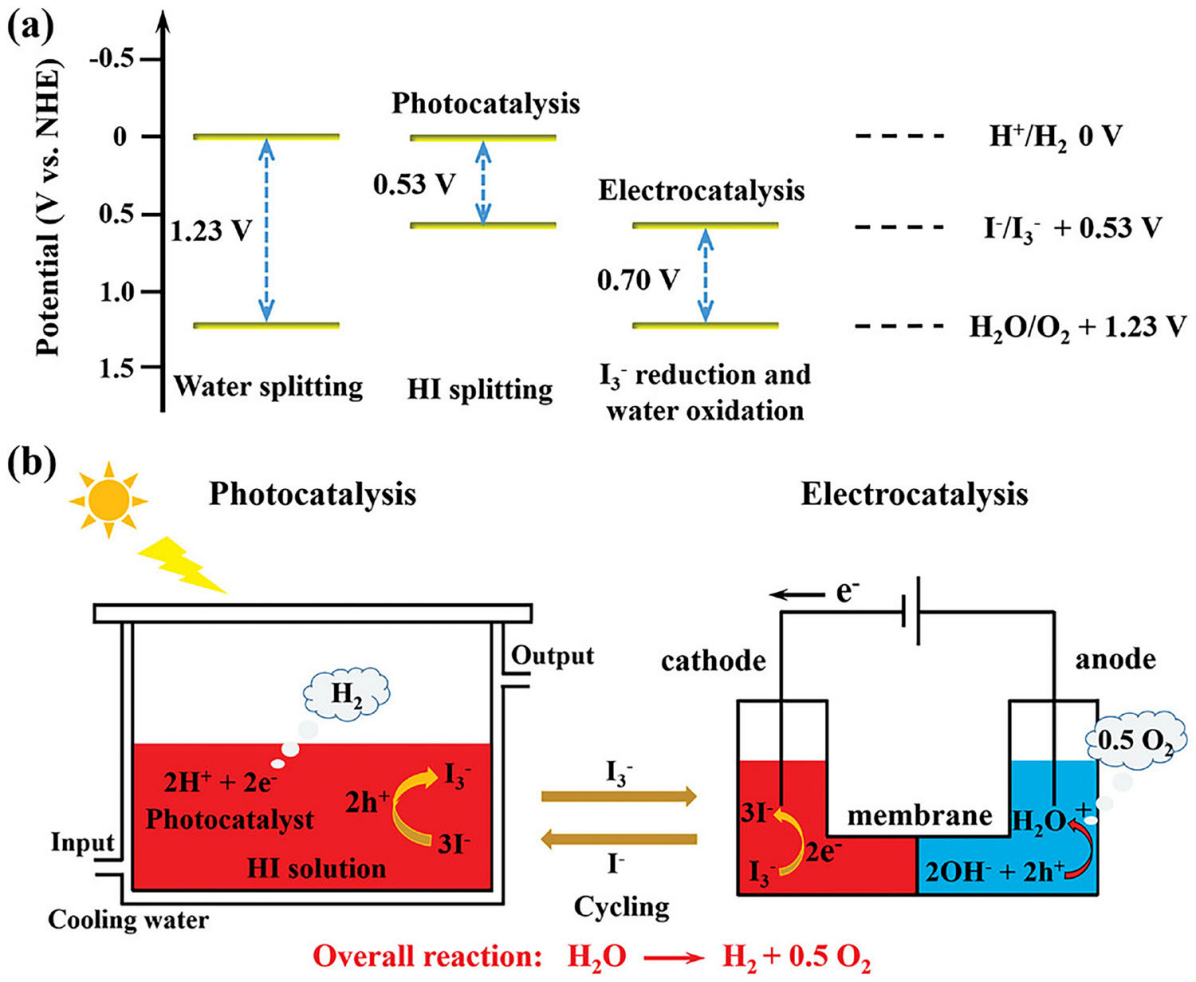
a) Redox potentials for the photocatalytic/electrocatalytic system for sequential HI splitting and water oxidation and b) schematic illustration of the steps involved. Reproduced with permission from ref. [197]. Copyright 2023 Wiley‐VCH.

A more recent study combines photocatalytic HI splitting with electrocatalytic water oxidation to carry out OWS using I_3_^−^/I^−^ as a redox shuttle between the two systems (**Figure** **11**).^[^ ^197^ ^]^ The authors couple a metal chalcogenide co‐catalyst (MoSe_2_) to a mixed‐halide perovskite, MAPbBr_3‐x_I_x_, and observe the same positive effects as mentioned for the ML‐MoS_2_ system discussed above. In fact, both the 1T and 2H phases of MoSe_2_ are also detected in this study, with an optimized loading of 3wt% MoSe_2_. The importance of the more catalytically active 1T phase is illustrated by the reduced performance of 3wt% 2H‐MoSe_2_/MAPbBr_3‐x_I_x_. The highest performing system yielded an HER rate of 3440 µmol h^−1^, 153‐ and 2‐fold higher than the pure perovskite or its Pt‐loaded counterpart, respectively. In the presence of H_3_PO_2_ as a sacrificial agent to reduce I_3_^−^, the authors reported an STH of 3.85% and an AQY of 37.67% at 530 nm. Without H_3_PO_2_, an impressive STH of 1.02% is obtained when HI splitting and water oxidation are performed sequentially. While offering an elegant way to circumvent the large overpotential required to drive OWS, one major drawback of this system is the sequential HI splitting/water oxidation, which requires double the time compared to another system performing concerted hydrogen and oxygen evolution. Furthermore, the activity of the photocatalyst seems to drop progressively after the first cycle.

#### 3.4.2 Lead‐free halide perovskites

Replacing lead with non‐toxic metal cations is an important step towards a system suitable for commercialization. Various substitutions have been tested since 2018, but performances remained weaker than lead‐based counterparts until a study published in 2020 reported an activity comparable to MAPI using Cs_3_Bi_2x_Sb_2‐2x_I_9_.^[^ ^181^ ^]^ Lead‐free perovskites tend to display superior stability towards moisture compared to their lead‐based counterparts, as illustrated by the higher number of applications in the context of water splitting. These developments are discussed below.

##### Compositional Engineering

**Figure 12:**
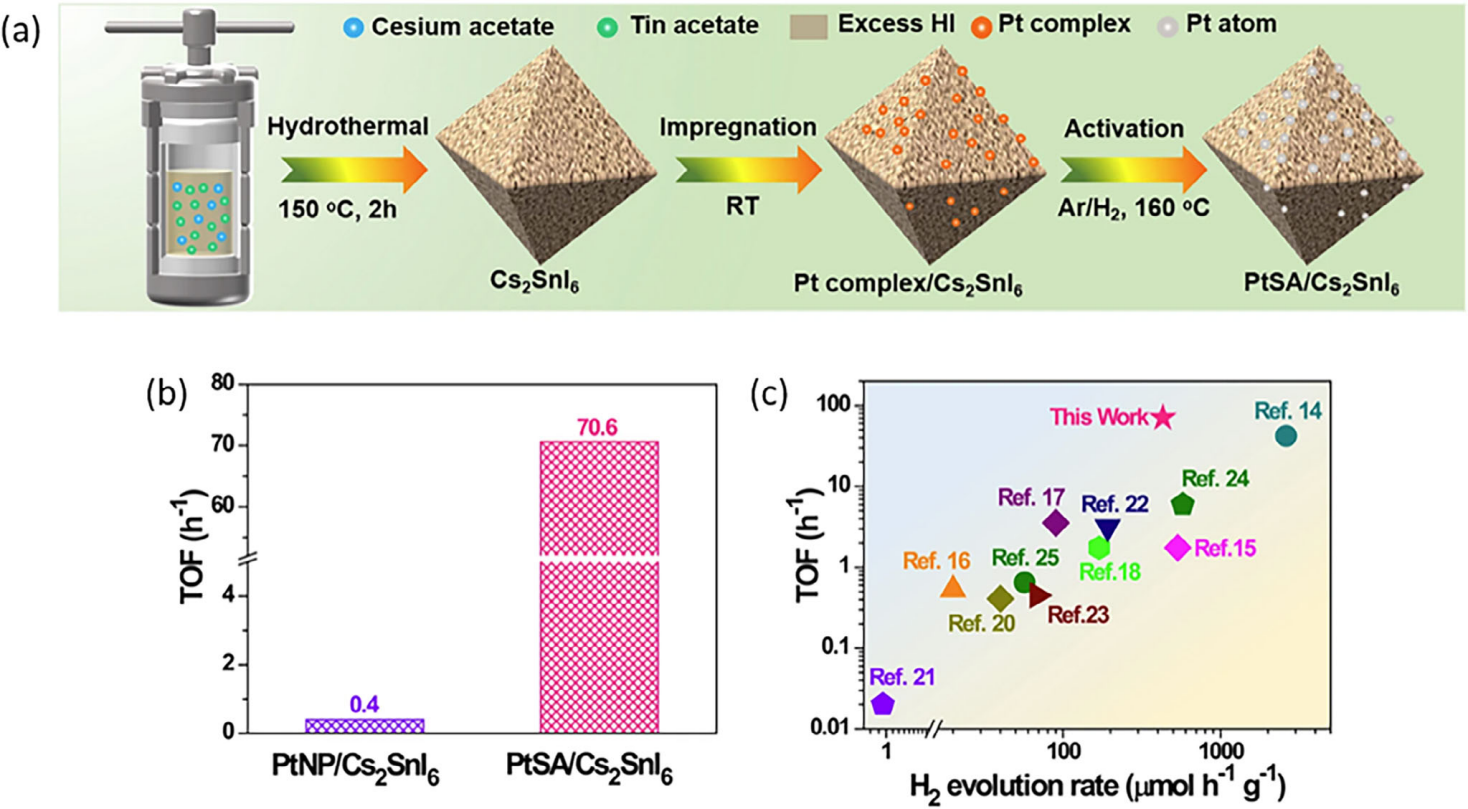
a) Schematic illustrating fabrication procedure of Cs_2_SnI_6_ photocatalyst decorated with PtSA as co‐catalyst. b) TOF of the perovskite photocatalyst with Pt nanoparticles vs single‐atoms. c) Literature comparison of TOF values, showing the record achieved for PtSA/Cs_2_SnI_6_. a–c) Reproduced with permission.^[^ ^186^ ^]^ Copyright 2021. Springer Nature.

Bi and Sn are common substitutes for Pb in lead‐free perovskites. In 2018, DMASnI_3_ was found to be stable in water for 16 h,^[^ ^182^ ^]^ and was tested as a photocatalyst for hydrogen evolution/methanol oxidation. A very modest HER rate of 0.64 µmol h^−1^ was reported, but the authors state that O_2_ evolution from water oxidation induces Sn oxidation. One study employing a Bi‐based perovskite reported photocatalytic activity based on a mechanism of charge transfer by collision, initially reported for a CdZnS‐based HER photocatalyst.^[^ ^198^ ^]^ In the study in question,^[^ ^102^ ^]^ it was suggested that electron transfer occurs via a collision between dispersed MA_3_Bi_2_I_9_ powder and dissolved Pt^2+^ ions, generating 6.76 µmol H_2_ h^−1^, in contrast to 0.49 µmol h^−1^ without Pt. The material's activity corresponds to an STH of 0.48%. The first lead‐free system yielding comparable results to that of MAPI was reported in 2020 and was based on Pt‐decorated Cs_3_Bi_2x_Sb_2–2x_I_9_ with *x* = 0.3 (labeled CBSI‐0.3). They report an AQY of 1.21% at 420 nm. The incorporation of Sb alongside Bi as B‐site cations is suggested to be the main reason behind the enhanced performance due to the reduction in Bi‐vacancy defect sites. Employing a tin‐based (Sn^4+^) double perovskite (Cs_2_Pt_x_Sn_1−x_Cl_6_), one study showed that by introducing different amounts of Pt^4+^, the material displays switching behavior between photoluminescence and photocatalytic proton reduction in water.^[^ ^188^ ^]^ The latter process occurred at the lower Pt concentrations, namely Cs_2_Pt_0.05_Sn_0.95_Cl_6_. The photocatalytic activity at low Pt composition was attributed to the higher density of sub‐gap states promoting thermal de‐trapping of electrons, thus suppressing radiative decay and increasing the number of electrons available to drive proton reduction. The authors report that the perovskite is stable during immersion in water for 25 days, and attribute this to the formation of an amorphous tin oxide protective layer.^[^ ^199^ ^]^ Similar to the principle of single‐atom Pt reported in the study on FAPbBr_3‐x_I_x_ discussed above,^[^ ^90^ ^]^ a new way of forming single‐atom Pt active sites was reported for a lead‐free system (**Figure** **12**).^[^ ^186^ ^]^ The authors relied on a Sn‐based MHP to mitigate the issue of lead toxicity. Furthermore, the all‐inorganic Cs_2_SnI_6_ perovskite was found to be more stable in HI aqueous solution than those based on organic cations. While the HER rate (4.3 µmol h^−1^) is less than in other systems, the novelty of the study is the formation of single‐atom Pt‐I_3_ sites. They report a record TOF of 70.6 h^−1^, indicating much more efficient utilization of Pt active sites compared to the report on FAPbBr_3‐x_I_x_/PtSA.^[^ ^90^ ^]^ The authors suggest three key reasons behind their system's performance: i) coordination structure, whereby I^−^ acts as anchoring sites, leading to atomic dispersion of Pt coordinated with three I^−^; ii) electronic properties of the Pt–I_3_ species, which promote more efficient charge separation and transport; and iii) higher electron density at single‐atom Pt sites compared to Pt NPs due to the favorable metal‐support interaction (SMSI). TOF for the single‐atom system is 176.5 times higher than that of Cs_2_SnI_6_ decorated with Pt NPs (0.4 h^−1^).

##### Perovskite/Semiconductor Heterojunctions

A perovskite/perovskite heterojunction has also been reported, with the aim of mitigating recombination in Bi‐based perovskites. The authors employ MA_3_Bi_2_I_9_/DMA_3_BiI_6_ (BBP‐5) as a binary, lead‐free, perovskite heterostructure and achieve a rate of 19.82 µmol h^−1^ without a noble metal co‐catalyst.^[^ ^185^ ^]^ This constitutes a 15‐ and 8‐fold improvement relative to the individual components, respectively. The heterostructure is formed in situ and requires control of the amount of DMF used, which acts as a co‐solvent and as the source of DMA^+^ cations. At 5 vol% DMF, the resultant type‐II heterojunction leads to efficient charge separation and suppressed recombination. Following the same rationale as for the lead halide perovskite/organic semiconductor heterojunctions, such heterojunctions were adopted for lead‐free systems. Similar to the enhancement observed by replacing MAPI‐Pt with MAPI‐rGO (discussed above), a comparable effect was observed using Cs_2_AgBiBr_6_.^[^ ^180^ ^]^ While photo‐deposition of 2.5wt% rGO did lead to a 51‐fold improvement in HER rate compared to the Pt‐analogue, the efficiency of the system is limited by the wider and indirect band gap in Bi‐based perovskites,^[^ ^200^ ^]^ evidenced by the more modest AQY of 0.16% at 450 nm, compared to 1.5% in the case of MAPI‐rGO. One group relied on the expected superior stability of 2D perovskites to test PEA_2_SnBr_4_ for H_2_ generation under sacrificial water splitting conditions, with TEOA as a hole scavenger.^[^ ^189^ ^]^ Having confirmed the material's stability in water over a period of 4 h, they employ the lead‐free perovskite in a composite with g‐C_3_N_4_. At a low perovskite composition of 5 wt% and with 3 wt% Pt as a co‐catalyst, they observe a HER rate of 33.9 µmol h^−1^. The neat perovskite with 3 wt% Pt displayed a very modest rate of 0.084 µmol h^−1^. The same group later also explored the 3D perovskite DMASnBr_3_ in a similar system,^[^ ^183^ ^]^ encouraged by the relative stability of DMASnX_3_ towards water (X = Br or I),^[^ ^182^, ^201^ ^]^ The highest‐performing composite DMASnBr_3_/g‐C_3_N_4_ contained 33 wt% DMASnBr_3_ and 3 wt% Pt, leading to a HER rate of 36.3 µmol h^−1^ with TEOA as hole scavenger. Based on the favorable impact of nitrogen‐doped carbon materials on electron transport and lifetime,^[^ ^202^ ^]^ one study in 2021 relied on nitrogen‐doped carbon support (N‐C‐140) in their CABB‐based composite.^[^ ^179^ ^]^ Cs_2_AgBiBr_6_/N‐C‐140 yielded an AQY of 0.59% at 420 nm, an improvement relative to CABB‐rGO mentioned above. Apart from the favorable CB alignment to promote electron transfer onto N‐C‐140, the material also performs the role of support onto which CABB is grown and helps prevent aggregation. In fact, a physical mixture of CABB and N‐C‐140 leads to only 25% of the activity achieved with in‐situ growth. The optimized composite evolved H_2_ at a rate of 3.8 µmol h^−1^, 19 times higher than pure CABB. While the aforementioned study relied on g‐C_3_N_4_ as a precursor to make N‐C‐140, g‐C_3_N_4_ is a promising visible‐light, metal‐free semiconductor photocatalyst in its own right.^[^ ^203^ ^]^ Various lead‐free halide perovskite/g‐C_3_N_4_ composites have also been reported in the context of HX splitting, employing either CABB or Cs_3_Bi_2_Br_9_.^[^ ^184^, ^187^ ^]^ *In‐situ* self‐assembly of the CABB composite yields a type‐II heterojunction where H_2_ evolution occurs on the CABB at a rate of 3 µmol h^−1^, a modest 2.5‐fold improvement on the neat perovskite (1.2 µmol h^−1^).^[^ ^184^ ^]^ A different mechanism is proposed for Cs_3_Bi_2_Br_9_/g‐C_3_N_4_/Pt (3wt%), where the best‐performing composition involves a low perovskite content (namely 2.5wt%). The composite yields a HER rate of 22.05 µmol h^−1^.^[^ ^187^ ^]^ According to the proposed mechanism, self‐trapping of photogenerated electrons occurs on the perovskite at higher contents, leading to a drop in HER rates. Inspired by an effect observed in dye‐sensitized solar cells, they suggest a direct excitation from the VB of the perovskite to the CB of g‐C_3_N_4_ is effective at low perovskite content.^[^ ^187^ ^]^

Porous g‐C_3_N_4_ (pCN) has also been combined with 16.7 wt% CsPbI_3_ and decorated with 3.9 wt% Pt. In the resultant type‐II heterojunction, H_2_ evolution occurred on pCN at a rate of 10 µmol h^−1^ while sacrificial oxidation of TEOA occurred on the perovskite. It is worth noting that proton reduction is a competing reaction when performing CO_2_ reduction. In fact, negligible H_2_ evolution has been observed as a by‐product in gas‐phase CO_2_ reduction performed with concomitant water oxidation (without a hole scavenger),^[^ ^204^ ^]^ but further work is needed to understand the feasibility of water oxidation on halide perovskites and how to control it, as evidenced by the studies reviewed above.

### 3.5 Lead‐Halide Perovskites for Photoelectrochemical Water Splitting

As mentioned in section 3.1, PEC and PV‐EC systems are two of the main technologies for green H_2_ production. PEC systems have historically been limited by low solar to fuel conversion efficiencies, while PV‐EC systems have been limited by complex device configurations and high costs.^[^ ^205^ ^]^ Meeting the US Department of Energy target of achieving <$2 kg^−1^ H_2_ requires an STH of > 20% and a device lifetime of >10 years.^[^ ^206^ ^]^ However, after over four decades of research, traditional earth‐abundant materials for PEC systems, including metal oxides, metal nitrides, III‐V materials, and crystalline silicon have low STH values well below this target, and poor stability.^[^ ^207^ ^]^ Lead‐halide perovskites offer opportunities to achieve improved performance, and using integrated PV‐PEC systems provides an opportunity to make use of the extensive work in the wider field of fabricating efficient LHP photovoltaic devices. LHPs have a theoretical STH of 28.7% for single‐junction photoelectrodes,^[^ ^207^ ^]^ and can readily be combined with silicon photovoltaics to achieve efficient tandem devices that have now enabled integrated PV‐PEC systems with >20% STH.^[^ ^208^ ^]^ This section discusses the advances made in the application of LHPs for water splitting in terms of performance, but also in terms of durability.

#### 3.5.1 Single‐Junction Photoelectrodes

LHPs readily dissolve in water, which severely limits the ability to directly use halide perovskites as photoelectrodes for water splitting in direct PEC systems.^[^ ^82^, ^139^, ^209^ ^]^ Over the past decade, substantial progress has been made to overcome this limitation to enable LHPs to be feasible photoelectrodes through the use of effective encapsulation methods (discussed later in section 3.5.3). At the same time, unlike traditional photoelectrodes, in which the bare photoactive layer is often used, LHPs benefit from the use of the charge transport layers developed by the photovoltaics field to enhance the separation and extraction of photogenerated charge carriers to the catalyst. The use of these charge transport layers also improves the open‐circuit voltage, leading to a higher onset potential, thus improving the ability of these devices to overcome the overpotentials necessary to drive solar water splitting.

**Figure 13:**
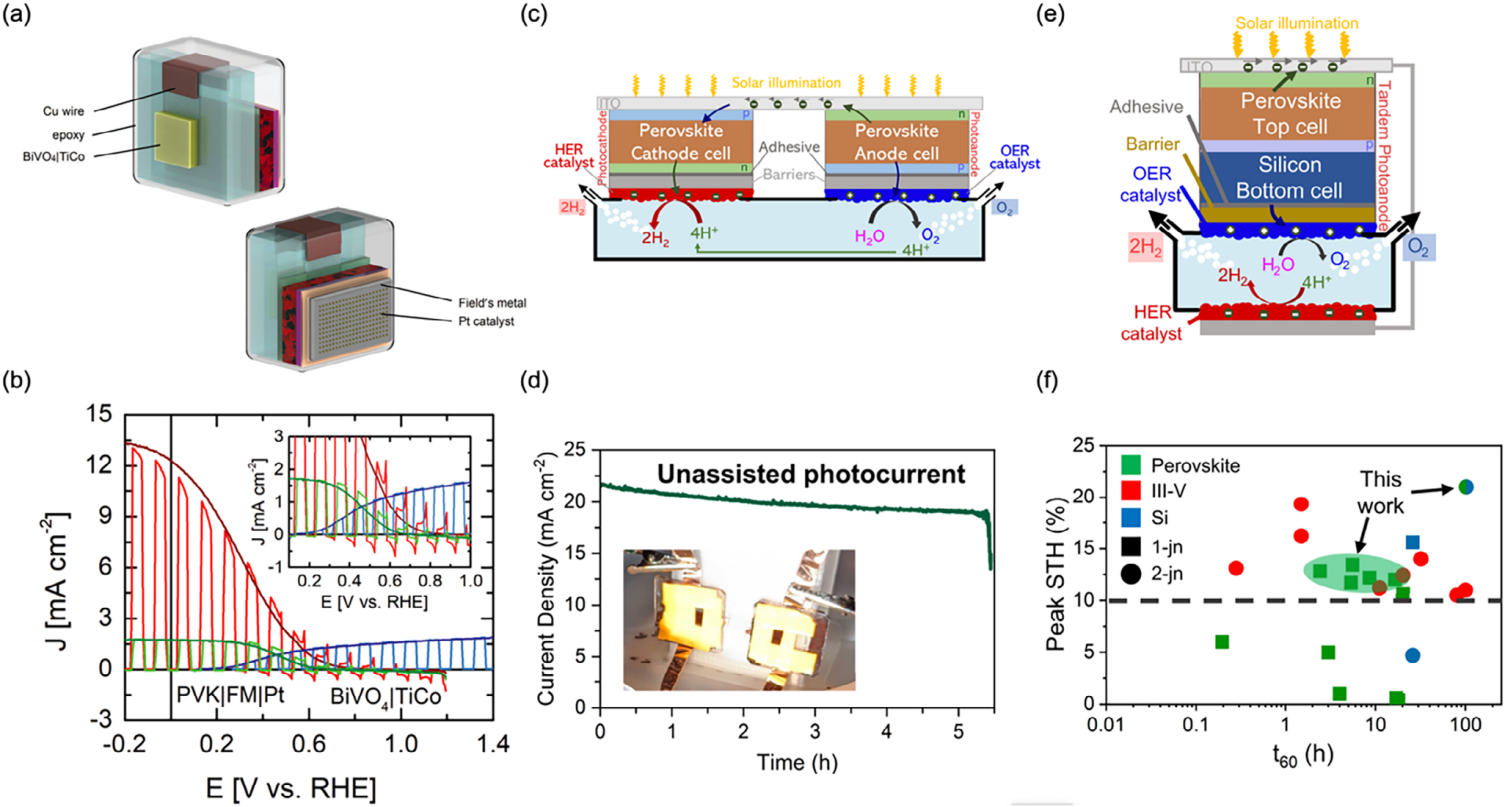
Device architectures for halide perovskite photoelectrodes. a) Photocathode formed from a metal‐halide perovskite heterojunction device, connected to a BiVO_4_ photoanode. b) Continuous and chopped voltammograms of BiVO_4_/TiCo photoanodes (blue), and halide perovskite photocathode without filtering (blue), and with filtering from BiVO_4_/TiCo (green). The device area was 0.25 cm^2^. Parts (a) and (b) are reproduced with permission from Ref.[211] Copyright 2018, Wiley. c) Tandem device with halide perovskite photoelectrodes connected together to achieve unassisted water splitting, and d) stability of the photocurrent with no external bias applied. e) Unassisted water splitting from perovskite‐silicon tandem photoanode, combined with a Pt/C hydrogen evolution reaction (HER) catalyst. f) Comparison of the peak solar‐to‐hydrogen (STH) efficiency of a range of PEC devices based on different light‐harvesting materials. “This work” refers to devices based on halide perovskite‐silicon tandem photoanodes. Parts (c) to (f) are reproduced under the terms of the CC‐BY license from Ref. [208].

LHPs are typically intrinsic or close to being intrinsic, and so are sandwiched between electron‐ and hole‐selective transport layers (ETLs and HTLs, respectively). By adjusting the order of these carrier‐selective contact layers when deposited onto the transparent conducting oxide (TCO)‐coated substrate, the devices can be prepared as photocathodes (*p*‐*i*‐*n*‐structured devices) or photoanodes (*n*‐*i*‐*p*‐structured devices). The materials used as charge‐transport layers significantly influence the STH and stability of the devices. For example, replacing poly(3,4‐ethylenedioxythiophene):poly(styrenesulfonate) (PEDOT: PSS) with NiO*_x_* as the hole transport layer improved the open‐circuit voltage of *p*‐*i*‐*n*‐structured perovskite photovoltaics from 0.6–0.9 V (PEDOT: PSS) to 1.00 ± 0.02 V (NiO*_x_*), and this led to an improvement in the early‐onset potential of these devices used in photocathodes (refer to **Figure** **13**a for an illustration of the device). Improvements in open‐circuit voltage and early onset potential were also achieved by covering the PEDOT:PSS layer with poly[bis(4‐phenyl)(2,4,6‐trimethylphenyl)amine], or PTAA.^[^ ^154^ ^]^ This reduced interface recombination,^[^ ^210^ ^]^ thus giving rise to an increase in open‐circuit voltage to 1.04 ± 0.02 V. The PTAA layer was also doped with 2,3,5,6‐tetrafluoro‐7,7,8,8‐tetracyanoquinodimethane (F4TCNQ) to improve the conductivity of this layer. Increasing the early onset potential of these photocathodes leads to greater overlap in their cyclic voltammetry (*CV*) curves with the *CV* curves of the photoanode (see Figure 13b for an example),^[^ ^211^ ^]^ thus enabling the development of self‐driven photoelectrochemical tandem devices (see section 3.5.2 for more details). The use of PTAA rather than NiO*_x_* to improve the open‐circuit voltage of these devices is advantageous in that PTAA can be deposited at lower temperatures than NiO*_x_*, and is compatible with flexible polymer substrates (rather than bulky glass substrates), which enabled the demonstration of lightweight, floating LHP photoelectrochemical tandem devices.^[^ ^154^ ^]^ Similarly, for *n*‐*i*‐*p*‐structured devices, making use of charge transport layers that have enabled reduced interface recombination has led to photoanodes with improved performance.^[^ ^208^ ^]^ There is therefore substantial work that can be done in the LHP photoelectrochemical field by drawing off the many varied improvements made in the photovoltaics field.

LHP photoanodes can be achieved using *n*‐*i*‐*p*‐structured devices with an oxygen evolution catalyst (e.g., Ni, NiFe, or NiFeOOH) connected to the hole transport layer.^[^ ^82^ ^]^ The advantage of LHPs is that their bandgaps can be readily tuned through the composition. MAPbI_3_ and triple‐cation perovskites, which have been widely explored for photocathodes, have also been investigated for photoanodes. However, the valence band maximum of MAPbI_3_ is too high for water oxidation to oxygen.^[^ ^209^ ^]^ Replacing I with Br in these LHPs increases the ionization potential, such that water oxidation can proceed. For example, CsPbBr_3_ and FAPbBr_3_ have both been demonstrated in photoanodes, achieving photocurrent densities of up to 3.8 mA cm^−2[^ ^82^ ^]^ and 8.5 mA cm^−2^ photocurrent density^[^ ^212^ ^]^ at 1.23 V (RHE), respectively. The latter exceeds the photocurrent of state‐of‐the‐art metal oxide‐based photoanodes (5–7 mA cm^−2^ at 1.23 V (RHE)).^[^ ^207^ ^]^ Very recently, a substantially higher photocurrent of 22.82 mA cm^−2^ was achieved at 1.23 V (RHE) using an α‐phase FAPbI_3_ perovskite photoanode, encapsulated with Ni foil, and with a NiFeOOH oxygen evolution reaction photocatalyst^[^ ^207^ ^]^ (as measured in a three‐electrode setup). α‐FAPbI_3_ has a deeper band position than MAPbI_3_, although, the same spiro‐OMeTAD hole transport layer was used.^[^ ^207^ ^]^ When these α‐FAPbI_3_ photoanodes (0.25 cm^2^ active area) were wired to an external α‐FAPbI_3_ photovoltaic device, and also wires to a Pt cathode (dark; only acts as an electrocatalyst) to achieve a hybrid PEC‐PV device, unassisted solar water splitting with an STH of 9.8% was realized.^[^ ^207^ ^]^

#### 3.5.2 Perovskite‐Based Tandem Devices for Photoelectrodes

Single‐junction halide perovskites face significant limitations when it comes to unassisted solar water splitting. The open‐circuit voltage produced by a single‐junction lead‐iodide‐based device falls below the minimum practical voltage required to drive both the hydrogen and oxygen evolution reactions (1.5–1.9 V).^[^ ^213^ ^]^ Increasing the bandgap of the halide perovskite increases the open‐circuit voltage (but also increases non‐radiative voltage losses) but at the cost of a reduced photocurrent. This trade‐off can be overcome by combining multiple photovoltaic devices together, harvesting light from complementary parts of the solar spectrum. This can be in the form of separate devices operating as the photocathode and photoanode (see Figure 13c for an example), or from a tandem photovoltaic device either driving an electrocatalyst or integrated into a photoelectrochemical system (see Figure 13e for an example).

For example, Mohite and co‐workers recently developed *p*‐*i*‐*n* photocathodes based on triple‐cation halide perovskites, and *n*‐*i*‐*p* photoanodes based mostly on α‐FAPbI_3_, with a small amount of methylammonium mixed into the A‐site.^[^ ^207^ ^]^ The perovskite photocathode had a Pt hydrogen evolution catalyst, while the perovskite photoanode had an IrO*_x_* oxygen evolution catalyst (Figure 13c). The back contacts of these two devices were connected, and, when both photoelectrodes were illuminated with AM 1.5G radiation, were shown to achieve unassisted solar water splitting with an initial photocurrent of 22.1 mA cm^−2^ and STH of 13.4% (Figure 13d). These devices operated for just over 5 h, with an average decrease in photocurrent of approximately 0.5 mA cm^−2^ h^−1^ before failure (Figure 13d).^[^ ^207^ ^]^

A similar wired design was demonstrated earlier by Andrei et al. for halide perovskite (photocathode)–BiVO_4_ (photoanode) tandems.^[^ ^154^ ^]^ In both cases, Pt was used as the hydrogen evolution catalyst, while the BiVO_4_ photoanode used TiCoO*_x_* oxygen evolution catalysts. Increasing the open‐circuit voltage of the halide perovskite photocathode to > 1 V by reducing interface recombination between the perovskite and hole transport layer (by using an interfacial layer of PTAA) was important to increase the overlap in the CV curves of photocathode and BiVO_4_ in order to achieve unassisted solar water splitting. The STH reached was <1% in both cases.^[^ ^154^, ^211^ ^]^ An important loss in performance came from the shading of the photocathode by the photoanode when these devices were wired together back‐to‐back (Figure 13a).^[^ ^211^ ^]^ This was overcome by having the two photoelectrodes placed next to each other, thereby taking a larger area, but avoiding any shading losses (see Figure 13c for an example).^[^ ^154^, ^207^ ^]^

An important limiting factor of the wired‐tandem design described above is that it is difficult to achieve an ideal overlap in the maximum power points of the two photoelectrodes. An alternative approach is to fabricate a monolithic tandem photovoltaic device that can provide sufficient photovoltage to drive unassisted solar water splitting. Mohite and co‐workers demonstrated this with a perovskite‐silicon tandem photovoltaic device (with 29% power conversion efficiency under 1‐sun illumination, along with 1.9 V open‐circuit voltage), with the Ir/IrO*_x_* oxygen evolution catalyst attached to the silicon cell. The perovskite top cell was wired to a Pt foil counter electrode for hydrogen evolution (Figure 13e). This PEC–PV‐EC hybrid device achieved unassisted solar water splitting with 20.8% STH. This peak STH exceeds previous reports of halide‐perovskite‐based PEC systems (Figure 13f), also exceeds the performance of III‐V tandem PEC systems,^[^ ^208^ ^]^ and is competitive with PV‐EC systems using a separate monolithic perovskite‐silicon tandem photovoltaic device driving electrolysis.^[^ ^214^ ^]^

Monolithic perovskite‐perovskite tandem devices for PEC–PV‐EC systems have also been demonstrated by Yan and co‐workers.^[^ ^213^ ^]^ A mixed iodide‐bromide perovskite top‐cell (1.75 eV bandgap) was monolithically integrated over a mixed Pb‐Sn perovskite bottom‐cell (1.25 eV bandgap), leading to photovoltaic devices with 26% power conversion efficiency and 2.1 V open‐circuit voltage. In a PV‐EC system, 17% STH was achieved (using Pt and IrO*_x_* catalysts). To create a hybrid system, the monolithic perovskite‐perovskite tandem was encapsulated with metal foil and graphite/Ag paste, and a Pt catalyst connected to the top electrode of the narrow‐gap perovskite device, while the back contact of the tandem was wired to an IrO*_x_* electrocatalyst. These hybrid systems achieved an STH of 15% and retained 95% of the initial performance after 120 h of continuous operation under chopped illumination. Whilst the performance of these devices is lower than that obtained from III–V tandems, technoeconomic analyses showed that the fabrication cost of the all thin film perovskite tandem is two orders of magnitude smaller, and hybrid PEC–PV‐EC systems that can operate for 3 years could reach a levelized cost of hydrogen of US$1 kg^−1^.^[^ ^213^ ^]^

#### 3.5.3 Improving Stability and Minimizing Lead Release from Halide Perovskite Photoelectrodes

While directly integrating a photovoltaic device into a photoelectrochemical system can improve the simplicity of the design, it places more stringent requirements on the stability of the device. Halide perovskites readily dissolve in water, and effective encapsulation is therefore essential. Early efforts at developing halide perovskite photoelectrodes encapsulated the perovskite device stack with metal foil. For example, a thin layer (8 nm) of nickel was used to protect *n*‐*i*‐*p*‐structured MAPbI_3_ photovoltaic devices, giving rise to the first demonstration of halide perovskite photoanodes.^[^ ^209^ ^]^ Nickel was selected because it had been shown previously to protect silicon photoanodes from corrosion and to act as an oxygen evolution reaction catalyst.^[^ ^139^, ^215^ ^]^ Although the Ni top layer was more effective than Au as a top layer, the stability of the halide perovskite photoanodes was only on the order of tens of minutes.^[^ ^209^ ^]^ However, the degradation of the photoanode was partly due to the low stability of the MAPbI_3_ perovskite layer. Recently, Hansora et al. used the more stable α‐FAPbI_3_ as the photoanode active layer, which was made into a photoanode with a similar architecture at the MAPbI_3_ device, and again with Ni foil encapsulation.^[^ ^207^ ^]^ But this time, the Ni foil was thicker (25 µm, rather than 8 nm), and this was critical for improving the stability of these photoanodes. When the Ni foil was <10 nm thick, the photoanodes were only stable for a few minutes because they were insufficient for preventing electrolyte permeation. Foils >10 µm thick were needed to prevent Pb leaching into the electrolyte after >5 h of operation, and these thick foils were attached to the perovskite device stack using Ag paste, which was adequate for forming an ohmic contact.^[^ ^207^ ^]^

**Figure 14:**
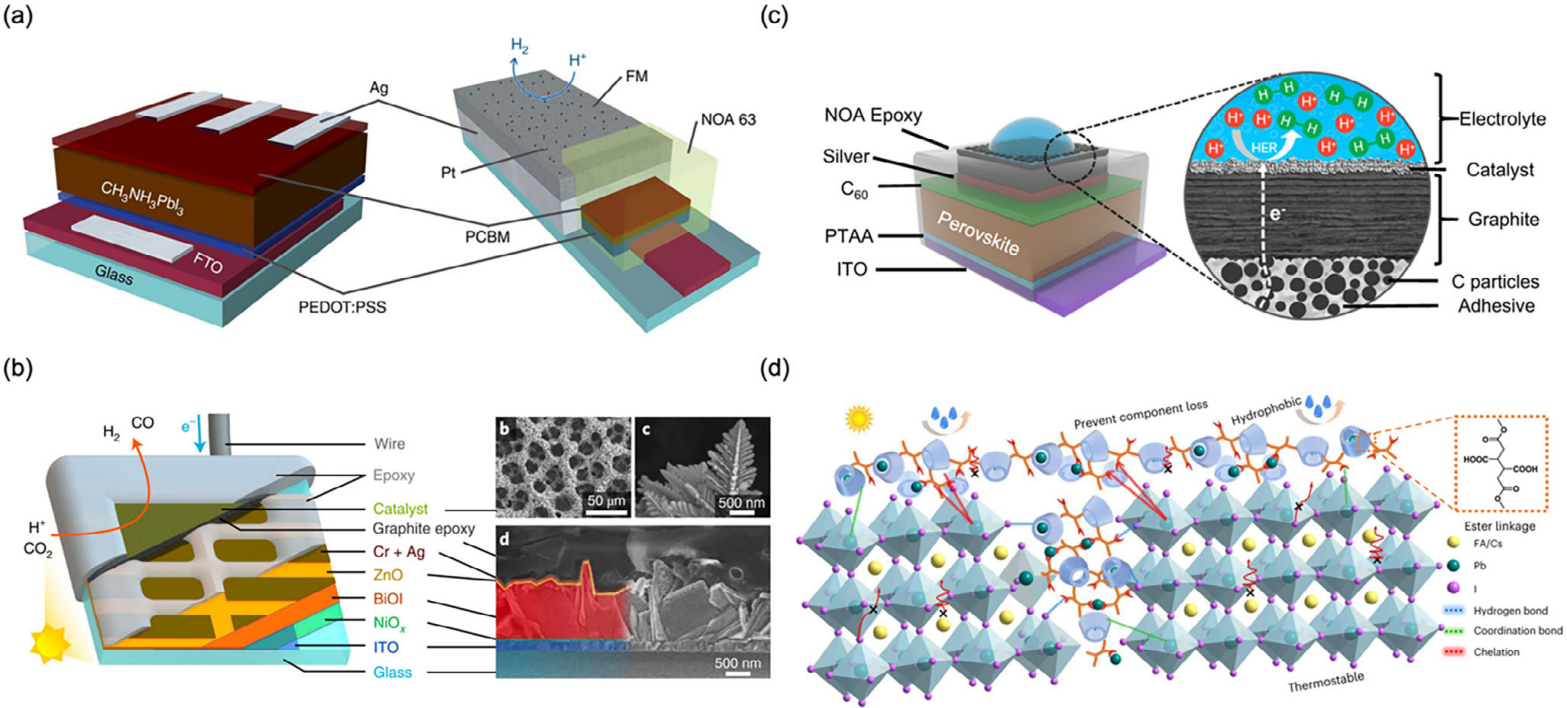
Mitigating the release of Pb from halide perovskite PEC devices. a) Illustration of the use of metallic encapsulating layers, such as Field's metal (FM). In this case, FM was deposited over a methylammonium lead iodide photovoltaic device, which was used as a photocathode by depositing Pt over the FM. Reproduced under the terms of the CC‐BY license from Ref. [131]. b) Illustration of BiOI photocathodes, which are stabilized using graphite epoxy. Reproduced with permission from Ref. [220] Copyright 2022, Springer Nature. c) Halide perovskite photocathodes stabilized with a conductive adhesive barrier (CAB), comprised of graphite sheets with a pressure‐sensitive adhesive. Reproduced under the terms of the CC‐BY license from Ref. [208]. d) Use of supramolecular complexes to sequester Pb^2+^ from degraded halide perovskite grains. The supramolecular complex in this case was comprised of cyclodextrin cross‐linked with butyltetracarboxylic acid. Reproduced with permission from Ref. [221]. Copyright 2023, Springer Nature.

A similar idea was used to protect monolithic perovskite‐perovskite tandems by using ≈100 µm thick Pt foil to encapsulate these devices and using a mixed graphite/Ag paste to glue these foils onto the device stack.^[^ ^213^ ^]^ Pt was also effective as a hydrogen evolution catalyst, and these device were stable under operation for 120 h.^[^ ^213^ ^]^ Leading on from the concept of metal‐foil‐encapsulation, Reisner and co‐workers introduced the idea of using Field's metal (eutectic alloy of 51% In, 32.5% Bi, and 16.5% Sn).^[^ ^131^ ^]^ This has the advantage of being able to directly integrate onto the perovskite devices, since this alloy melts at 62 °C, and can therefore be integrated by placing a solid piece of Field's metal onto the device and heating at 70 °C, which melts the Field's metal but does not degrade the perovskite device. As a result, the use of Ag paste is unnecessary. At the same time, the perovskite devices still had Ag top electrodes deposited because it was found that Field's metal effects wet this surface (**Figure** **14**a). Where Field's metal spread to could therefore be controlled by the pattern of the Ag electrode.^[^ ^131^ ^]^ Catalysts could then be deposited on top of the Field's metal, e.g., Pt for hydrogen evolution reactions. The stability of MAPbI_3_ photocathodes encapsulated with Field's metal was initially poor (degrading after only 2 h of operation),^[^ ^131^ ^]^ but was improved to >15 h by replacing the acidic PEDOT: PSS hole transport layer in contact with the perovskite thin film with NiO*_x_*.^[^ ^211^ ^]^

Further stability improvements were obtained by integrating conductive particles into an epoxy to provide more effective encapsulation, whilst still allowing photogenerated charge carriers to be transported to an electrocatalyst. Andrei et al. developed graphite epoxy by mixing in graphite powder with Araldite Standard epoxy adhesive.^[^ ^154^ ^]^ This is advantageous over Field's metal by requiring no heating and can be easily spread in a controlled manner, even when the substrate used is a polymer. As a result, Andrei et al. demonstrated the stable operation of the perovskite photocathodes for both H^+^ and CO_2_ reduction for >30 h and also were able to fabricate the photocathodes on flexible plastic substrates, giving rise to lightweight photocathodes that could float based on the bubbles produced.^[^ ^154^ ^]^ Such devices could be used in the future for offshore photoelectrochemical reactors for producing solar fuels. Graphite epoxy has also been demonstrated as an effective encapsulant for BiOI photocathode devices (Figure 14b).^[^ ^216^ ^]^ BiOI photocathodes directly used in contact with electrolytes degrade within minutes, and applying a bias to these devices results in Bi^3+^ being reduced to Bi^0^. Encapsulating these photocathodes with the oxide charge transport layers developed for photovoltaics,^[^ ^217^ ^]^ as well as graphite epoxy encapsulant, improves the stability to ≈500 h. Indeed, the BiOI photocathode was more stable than the Pt catalyst used for H^+^ reduction, which is unusual among novel light harvesters.^[^ ^217^ ^]^ Similarly, Mohite and co‐workers developed a conductive adhesive barrier, comprised of a graphite barrier glued to the perovskite device with a pressure‐sensitive adhesive mixed with conductive particles (Ag, Cu, and amorphous C) to improve the conductivity (Figure 14c).^[^ ^208^ ^]^ In photovoltaic devices measured in air, perovskite devices encapsulated with this conductive adhesive barrier retained 95% of their initial efficiency after 10 h of continuous maximum power point tracking, whereas perovskite photovoltaic devices covered only with graphite and carbon paste lost 40% of their initial performance. Encapsulating perovskite photoelectrodes, connected together in tandem (Figure 13c), with this conductive adhesive barrier enabled the tandem photoelectrochemical system to be stable for 5 h (Figure 13d) with >10% STH. Improved stability was achieved in monolithic perovskite‐silicon tandems (Figure 13e), which achieved unassisted water splitting for >100 h.^[^ ^208^ ^]^

Further enhancements in stability were achieved by Eslava and co‐workers using glassy carbon and boron‐doped diamond as encapsulation,^[^ ^218^ ^]^ achieving CsPbBr_3_ photoanodes with 168 h and 210 h stability, respectively. This was a substantial improvement over CsPbBr_3_ and FAPbBr_3_ photoanodes with graphite encapsulation, which has stabilities on the order of tens of hours.^[^ ^82^, ^212^, ^219^ ^]^ The limitation of graphite is that these layered materials suffer from chemical and mechanical stresses during operation, particularly when bubblers nucleate and grow. Both glassy carbon and B‐doped diamond are more chemically stable and maintain high electrical conductivity. These sheets were prepared separately to the perovskite device stack, and glued on using a thin layer of epoxy that did not completely isolate the perovskite device from these sheets, thus allowing photogenerated holes to still be transported through the encapsulation layer to the NiFeOOH catalyst loaded onto the sheet.^[^ ^218^ ^]^

Finally, it is important to consider what mitigation measures can be put in place to prevent the release of lead in aqueous solution following the catastrophic failure of encapsulation. This has led to the exploration of lead sequestration strategies, which make use of high surface area structures that can bind to Pb^2+^ before it is released into flowing water.^[^ ^222^ ^]^ One of these strategies is to use a supramolecular complex comprised of a β‐cyclodextrin derivative, linked together with 1,2,3,4‐butane tetracarboxylic acid (Figure 14d). These supramolecular complexes can be integrated into the perovskite active layer during thin film deposition and have been shown to reduce the leaching rate of Pb^2+^ by a factor of 18 without affecting device performance.^[^ ^221^ ^]^ As discussed above, a wide range of internal and external encapsulation strategies are being developed to enhance the stability of perovskites in polar solvents, thus reducing Pb's leaching into the solvent medium. Some of the external strategies include the encapsulation of perovskites with conductive materials like Ni, Pt, Au, alloys (Field's metal), graphite, carbon, conductive epoxy (conductive particles combined with epoxy), and organic supramolecules like cyclodextrins. Although these enhancement strategies enabled the operation of perovskite photoelectrodes from a few hours to a few days depending on the encapsulant, achieving long‐term stability (months and years) is still an outstanding challenge. The material space of encapsulants should be expanded while keeping in mind that they have to be highly conductive. For example, internal encapsulation through direct interactions with Pb^2^⁺ through lead‐coordinating functional groups such as carbonyl, hydroxyl, and thiol can effectively suppress lead leakage. Explored encapsulants including the use of functional hole‐transporting materials like spiro‐NPU that form Pb‐O bonds,^[^ ^223^ ^]^ thiol‐functionalized 2D MOFs with strong Pb‐S interactions,^[^ ^224^ ^]^ polymers like PBAT containing carbonyl groups,^[^ ^225^ ^]^ and small molecules like thiol‐functionalized perfluoroalkyls.^[^ ^226^ ^]^ In addition, one should think of designing photoelectrochemical devices in which the perovskites are not in direct contact with the reaction medium. Therefore, more studies should be devoted to improving the stability and thus reducing Pb leaching in polar reaction mediums.

#### 3.5.4 Strategies for Device Scale‐Up

**Figure 15:**
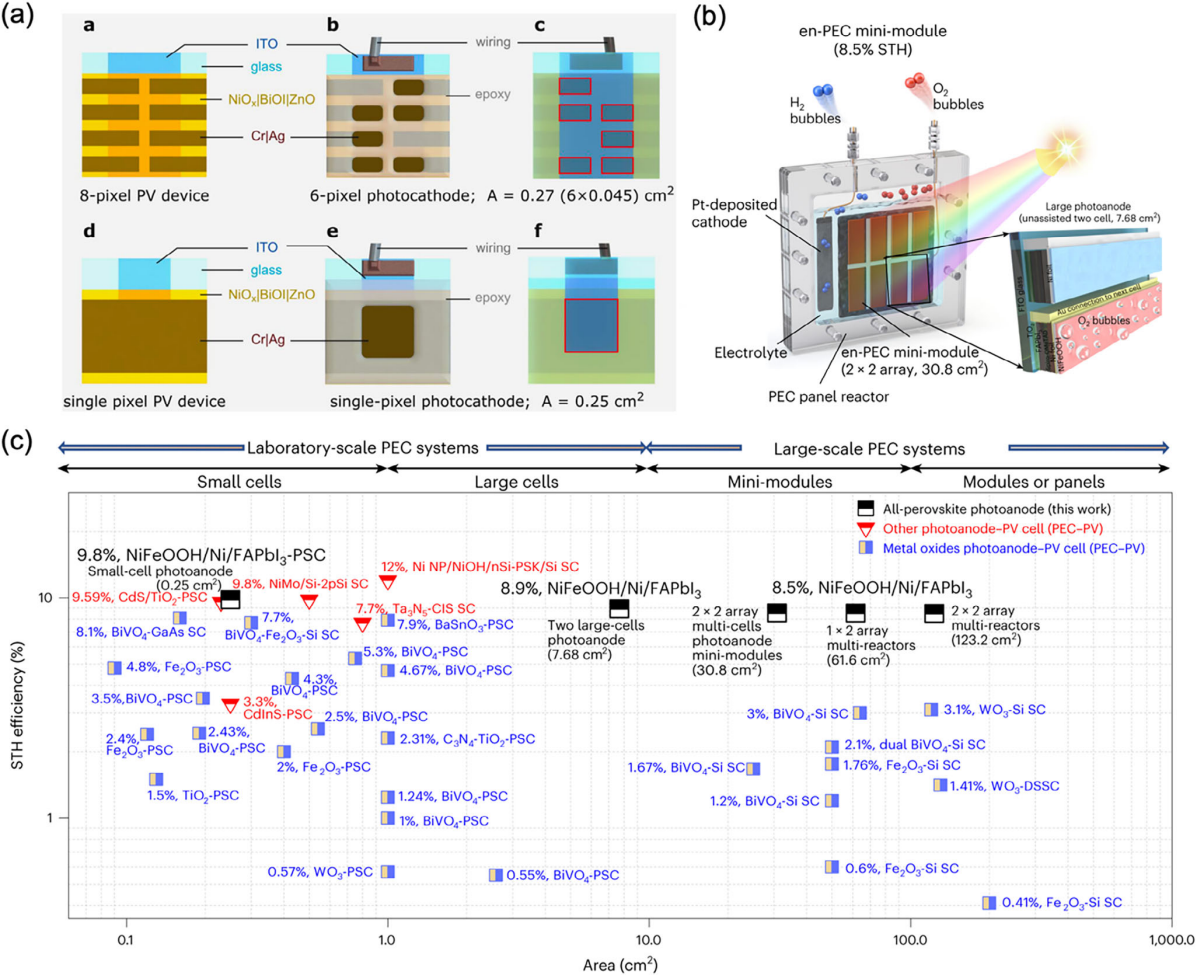
Strategies to scale up halide perovskite photoelectrodes. a) Illustration of two strategies for scale‐up: the traditional use of a large single‐pixel device, versus the combination of several smaller pixels that add up to a larger device area. Reproduced with permission.^[^ ^220^ ^]^ Copyright 2022, Springer Nature. b) Formation of mini‐modules of photoelectrochemical cells by combining halide perovskite photoanodes. c) Comparison of the solar to hydrogen (STH) conversion efficiency against device area for a wide range of photoelectrochemical cells, ranging from small cells to modules. Parts (b) and (c) are reproduced with permission.^[^ ^207^ ^]^ Copyright 2024, Springer Nature.

The conventional approach to scaling up devices is making one with a larger area. This leads to reductions in performance because of an increased likelihood of shunt pathways (e.g., pinholes in any of the thin film layers) being present, as well as increased series resistance from the longer pathway for charge carriers to travel through the transparent electrode. But unlike photovoltaics, in photoelectrochemical cells, it is not necessary to have one continuous large pixel. Rather, smaller pixels, operating simultaneously and side‐by‐side on a large substrate can be used since photoelectrochemical reactions take place on any exposed surface that has photogenerated charge‐carriers introduced to it (**Figure** **15**a).^[^ ^216^ ^]^ Andrei et al. demonstrated this concept for BiOI photocathodes, showing that combining several smaller pixels leads to a higher photocurrent and larger early onset potential because of reduced losses due to shunting, and this improved performance was essential to achieve unassisted water splitting working in tandem with BiVO_4_ photoanodes. Furthermore, the BiOI pixels can be pre‐screened to identify shunted or faulty pixels, and these can be covered using epoxy encapsulating.^[^ ^216^ ^]^

Similarly, Hansora et al. fabricated α‐FAPbI_3_ mini‐modules by combining multiple individual cells (3.84 cm^2^ each) altogether (Figure 15b). As a result, there was little decrease in the STH from small photoelectrochemical cells (1 cm^2^; 9.2% STH) to 123.2 cm^2^ area panels (8.5% STH). The STH values obtained from the large‐area panels are competitive compared to oxide‐based systems (Figure 15c). Furthermore, the 3.84 cm^2^ area devices had similar performance to the smaller lab‐scale 0.25 cm^2^ cells (20.5 mA cm^−2^ vs 22.82 mA cm^−2^ photocurrent at 1.23 V (RHE), respectively).^[^ ^207^ ^]^

## 4 CO_2_ Reduction

**Figure 16:**
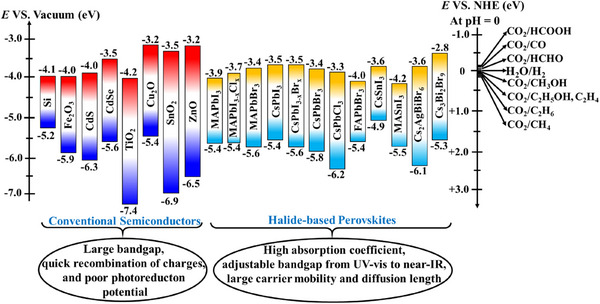
Conduction and valence band positions of conventional and halide‐based perovskite materials relative to CO_2_ reduction potential. The relative potentials at pH 0 involved in CO_2_ reduction are plotted versus vacuum (left). This plotting helps to compare the energy levels of different electrochemical reactions with a common reference point, which is the vacuum level.

Converting carbon dioxide (CO_2_) into fuels like methane or methanol using solar energy offers a promising solution to both environmental concerns and future energy needs.^[^ ^227^ ^]^ This process not only mitigates the increasing levels of CO_2_ in the atmosphere, but also offers a convenient means of energy storage.^[^ ^228^ ^]^ In this regard, several methods and materials have been developed primarily by harnessing solar energy for CO_2_ reduction.^[^ ^139^, ^229^, ^230^ ^]^ One of the early notable contributions to this field was made by Bard et al.^[^ ^231^, ^232^ ^]^ who developed a photocatalytic device based on a particle suspension system. This device was initially employed for water‐splitting studies, following the pioneering discovery of photocatalytic water splitting at titanium dioxide electrodes by Honda and Fujishima in the early 1970s.^[^ ^24^ ^]^ The utilization of solar energy in such photocatalytic systems enables the conversion of CO_2_ into useful fuels and has opened doors to sustainable energy and remediation. Ever since the early report of photocatalytic CO_2_ reduction in 1979,^[^ ^21^ ^]^ numerous research efforts have been devoted to finding suitable photocatalysts. They play a vital role in converting solar light into charge carriers, namely electrons and holes, which are then utilized for oxidation and reduction reactions.^[^ ^233^, ^234^, ^235^, ^236^ ^]^ A wide range of semiconducting materials, e.g., TiO_2_,^[^ ^237^, ^238^, ^239^, ^240^, ^241^ ^]^ Cu_2_O,^[^ ^242^, ^243^, ^244^, ^245^, ^246^, ^247^, ^248^ ^]^ CdS,^[^ ^249^, ^250^ ^]^ C_3_N_4_,^[^ ^251^, ^252^, ^253^, ^254^ ^]^ and perovskite oxides,^[^ ^255^, ^256^, ^257^, ^258^ ^]^ have been developed for solar‐driven photocatalytic CO_2_ reduction. However, most of these materials are far from ideal photocatalytic systems because of various limitations, e.g., rapid recombination of photo‐generated charge‐carriers, wide bandgaps, and poor photoreduction potentials (**Figure** **16**). Over the past decade, MHPs have received significant attention as one of the most promising photocatalysts for reduction of CO_2_ because of their high absorption coefficient,^[^ ^259^, ^260^, ^261^, ^262^, ^263^, ^264^, ^265^ ^]^ large charge carrier motility,^[^ ^259^, ^266^, ^267^ ^]^ and wide tunability of their optoelectronic properties through the composition (Figure 16).^[^ ^268^, ^269^, ^270^, ^271^ ^]^ In the last few years, several review articles have been published on halide perovskite‐based photocatalysts for CO_2_ reduction, which mainly address the stability issues and basic mechanisms.^[^ ^38^, ^40^, ^111^, ^227^ ^]^ However, demand remains for a timely and comprehensive review that explores deeper into the photoreduction mechanisms, reductants, and products, as well as the structure‐photocatalytic performances relationships of halide perovskites and their composites. In this section, we highlight recent advances in solar‐driven CO_2_ conversion into fuels using organic‐inorganic and all‐inorganic colloidal perovskite materials including their heterojunction construction, defects engineering, architectural modifications, and other strategies with the aim of enhancing the efficiency and selectivity of these materials for CO_2_ conversion.

### 4.1 Typical Experimental Setup and Mechanism of Photocatalytic CO_2_ Reduction

**Figure 17:**
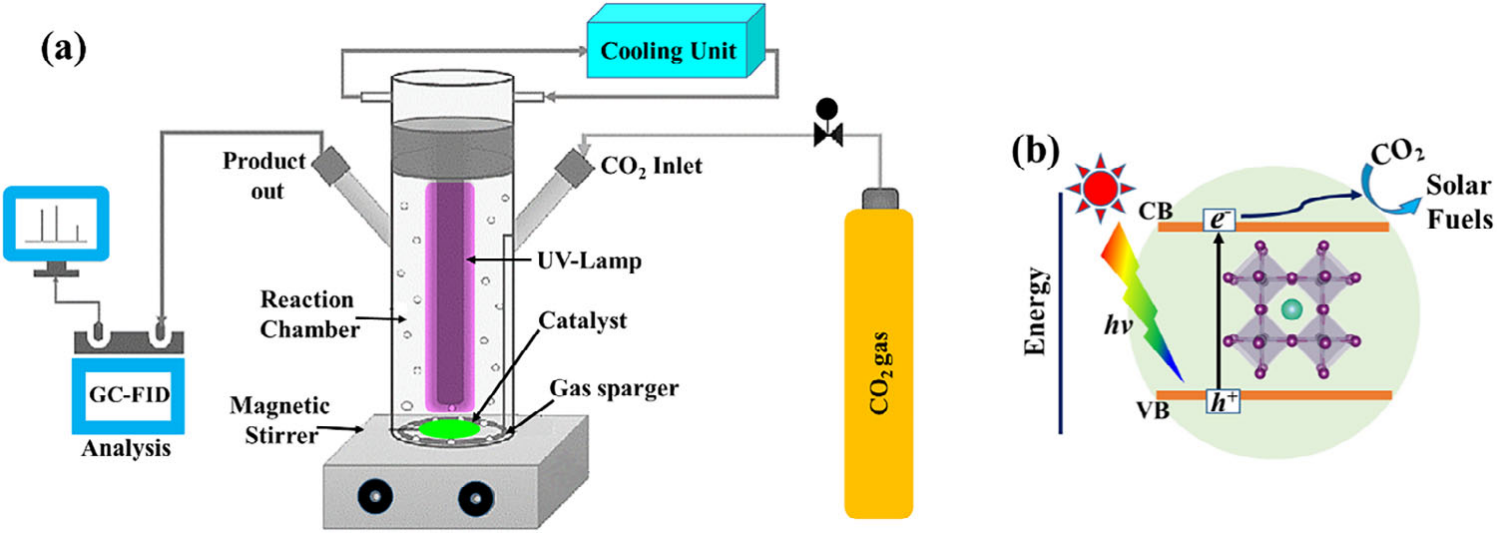
Schematic illustrations of a Photocatalytic reaction operator (a) and a typical mechanism for the photocatalytic CO_2_ reduction (b).

A photocatalytic reaction is generally carried out in a closed gas‐circulation reactor containing semiconductor particles (in the form of thin film, colloidal dispersion, or colloided deposited on a substrate) as the photocatalysts, and dissolved CO_2_ in various reaction medium (as shown in Table 3).^[^ ^233^, ^234^, ^235^, ^272^, ^273^ ^]^ The typical experimental setup is illustrated in **Figure** **17**. The reduction of CO_2_ takes place in the presence of semiconductor materials under light irradiation. The CO_2_ reduction process proceeds when the incident light has a photon energy equal or greater than that of the bandgap (*E*_g_) of the semiconductor materials. In general, the basic mechanism of the photocatalytic CO_2_ reduction consists of three synergistic steps (see Figure 17). In the first step, the absorption of light by the photocatalyst generates electrons and holes. Then, the second step involves the separation and migration of electrons to the interface, leaving an equal number of electrons and holes in the conduction band (CB) and valence band (VB), respectively. At this stage, a large fractions of photogenerated electron‐hole pairs could be consumed by recombination, because of the dominance of recombination over charge separation or vice versa, depending on their relative time scales. This recombination issue can be reduced by carefully engineering various structural and material properties. Creating complex interactions among factors such as dimension, surface properties, material crystallinity, and making heterostructures with co‐catalysts can improve the charge separation.^[^ ^38^ ^]^ In the final step, the transfer of electrons from the photocatalyst to respective sites, and then undergoes an electrochemical catalytic reaction to produce fuels. In general, an efficient photocatalyst should exhibit four important features; (i) the ability to enhance the charge separation and transfer them to reaction sites, (ii) the selectivity of photoreduction of CO_2_, (iii) durability in a peculiar environment (e.g., high humidity and high temperature), and (iv) suppressing the side reactions (e.g., H_2_ reduction). In addition, other factors such as photocatalyst loading, particle size, structure, dispersion, crystal facets, composition, alloy phase, morphology, and valence states play an important role in the efficiency and selectivity of photocatalytic CO_2_ reduction. In particular, the loading of an optimum amount of photocatalysts is important for maximum performance, otherwise, excess or lack of catalyst may lead to a reduction in the performance owing to the low yield and poor selectivity.

Photocatalytic CO_2_ reduction requires high energy because CO_2_ is one of the most thermodynamically stable carbon‐based molecules, with stronger C═O bonds (750 kJ/mol bond dissociation energy) than C−H bonds (411 kJ/mol bond dissociation energy) and C−C bonds (336 kJ mol^−1^ bond dissociation energy).^[^ ^274^ ^]^ Hence, CO_2_ needs to be activated before the photocatalytic reduction. This process occurs on the surface of photocatalysts through chemisorption by converting a linear molecule into a bent carbonate anion radical (CO_2_^−^) via monodentate or bidentate coordination. Thus, the reactive carbonate anion is responsible for the various products obtained from the photoreduction of CO_2_, as given in **Table** **2**. These hydrocarbon products are evaluated through two different pathways, one is carbine (highly reactive species that can further react to form various hydrocarbons) and the other one is aldehyde (which also undergoes further reduction to form hydrocarbons).^[^ ^275^ ^]^ Both pathways ultimately lead to the formation of methane (CH_4_) as a major product. Beside methane, other hydrocarbons such as ethane, ethylene, and propane (C1‐C3 products) are also produced as side products during the CO_2_ reduction process, which are shown in Table 2. It was proposed that the selective conversion of CO_2_ to CO or HCOOH by reactive CO_2_^−^ intermediates depends on the catalyst and media (aqueous or nonaqueous).^[^ ^276^ ^]^ In the aqueous media, the reactive CO_2_^−^ takes protons from water and photogenerated electrons from the catalyst leading to the formation of HCOOH or CO depending on the type of catalyst. However, the selectivity of the product is still challenging, and it depends on many factors, such as reaction condition, active site of the photocatalysts, medium, etc. The efficiency of the photoreduction of CO_2_ can be expressed in terms of CO_2_ conversion rate, product formation rate, turnover frequency (TOF), and quantum efficiency.^[^ ^277^ ^]^ Furthermore, the photocatalytic reduction of CO_2_ is a multielectron reduction process assisted by protons, and it consists of numerous intermediate steps that lead to different products (e.g., CO, formic acid, methanol, and methane), as shown in Table 2.^[^ ^18^, ^278^ ^]^

**Table 2:** Common products from CO_2_ reduction with their simplified redox equations.

S. No.	Equation	Product	*E°* (V)
01	CO_2_ + 1e¯ = CO_2_^−^	Carbonate anion radical	−1.90
02	CO_2_ + 2H^+^ + 2e^−^ = HCOOH (aq)	Formic acid	−0.61
03	CO_2_ + 2H^+^ + 2e^−^ = CO(g) + H_2_O	Carbon monoxide	−0.53
04	CO_2_ + 4H^+^ + 4e^−^ = HCOH (aq) + H_2_O	Formaldehyde	−0.48
05	CO_2_ + 6H^+^ + 6e^−^ = CH_3_OH(aq) + H_2_O	Methanol	−0.38
06	CO_2_ + 8H^+^ + 8e^−^ = CH_4_ (g) + 2H_2_O	Methane	−0.24

### 4.2 Organic–Inorganic Hybrid Perovskites

**Figure 18:**
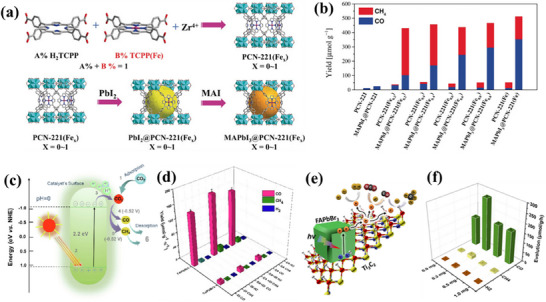
a) Schematic presentation for synthesis of PCN‐221(Fex) MOF and MAPbI_3_/PCN‐221(Fex) composite photocatalyst for stable and product‐selective CO_2_ reduction. b) Histogram of product formation using PCN‐221(Fex) and MAPbI_3_/PCN‐221(Fex) after 25 h of photocatalytic reaction. a,b) Reproduced with permission.^[^ ^279^ ^]^ Copyright 2019, Wiley‐VCH. c) Schematic illustration on band diagram of photocatalytic CO_2_ reduction into chemical fuels of FAPbBr_3_ QDs, and d) its product histogram in DI, the DI/EA system, and EA. (c) and (d) reproduced with permission from ref. [281]. Copyright 2021, with permission from Elseviere) Schottky heterojunction photocatalytic system for CO_2_ reduction in FAPbBr_3_/Ti_3_C_2_, and (f) its product histogram in deionized water. (e) and (f) reproduced with permission from ref. [280]. Copyright Copyright 2021, American Chemical Society.

The photocatalytic activity of organic‐inorganic hybrid perovskites (OIHP) towards CO_2_ reduction has been mainly focused using MAPbI_3_, and FAPbBr_3_ perovskites.^[^ ^28^ ^]^ It is well known that the halide perovskites suffer from poor stability due to their ionic character, leading to their degradation under moisture. Therefore, the halide perovskite photocatalyst needs to be encapsulated to obtain a stable photocatalyst. The encapsulant matrix could also assist in charge carrier separation upon excitation of a photocatalyst. In addition, the band gap and band position of halide perovskites are important to build efficient photocatalysts. Various strategies have been reported to encapsulate halide perovskites to obtain stable photocatalysts. For instance, Lu and co‐workers developed a series of efficient photocatalysts for CO_2_ reduction by encapsulating CH_3_NH_3_PbI_3_ (MAPbI_3_) perovskite NCs into earth‐abundant porous Fe‐porphyrin‐based metal‐organic framework (MOF) PCN‐221(Fe_x_), *x* = 0–1) by a sequential deposition process (see in **Figure** **18**).^[^ ^279^ ^]^ The MAPbI_3_/PCN‐221(Fe_x_) composite showed much improved photocatalytic activity towards CO_2_ reduction compared with their individual counterparts (MAPbI_3_ NCs and PCN‐221(Fe_x_), *x* = 0–1)). In addition, the composite photocatalyst exhibited record stability over 80 hours with a total production yield of 1559 µmol/g for CO (34%) and CH_4_ (66%). Besides, the halide perovskites combined with 2D materials exhibit enhanced photocatalytic activity. For example, FAPbBr_3_ NCs anchored on Ti_3_C_2_ nanosheets form a Schottky heterojunction (Figure 18) for an efficient photocatalytic CO_2_ reduction under visible light.^[^ ^280^ ^]^ In this composite system, the Ti_3_C_2_ ultrathin 2D nanosheet is an electron acceptor that promotes the prompt separation of excitons and helps to supply the charge carriers to specific catalytic sites. The FAPbBr_3_/Ti_3_C_2_ composite demonstrated significantly enhanced production of CO from CO_2_, which is over 1.84‐fold higher than that of pure FAPbBr_3_ quantum dots. In addition, the system exhibited an optimal electron consumption rate of 717.18 µmol g^−1^ h^−1^, which was far larger (2.08‐fold improvement) than that of 343.90 µmol g^−1^ h^−1^ observed of pristine FAPbBr_3_ QDs. Besides, pure perovskite NCs have also been explored for photocatalytic CO_2_ reduction. In this regard, Que et al.^[^ ^281^ ^]^ demonstrated that pure colloidal FAPbBr_3_ and CsPbBr_3_ NCs exhibit photocatalytic activity toward CO_2_ reduction in a mixture of deionized water/ethyl acetate solvent without further encapsulation. They found that the FAPbBr_3_ NCs exhibit much better catalytic activity than CsPbBr_3_ NCs towards CO_2_ reduction into CO. The CO production rate for FAPbBr_3_ NCs has reached 181.25 µmolg^−1^ h^−^1, which is ≈13.8‐fold higher compared to 9.73 µmol g^−1^ h^−1^ achieved with CsPbBr_3_ NCs.

### 4.3 All‐Inorganic Perovskite NCs and Their Composites

**Figure 19:**
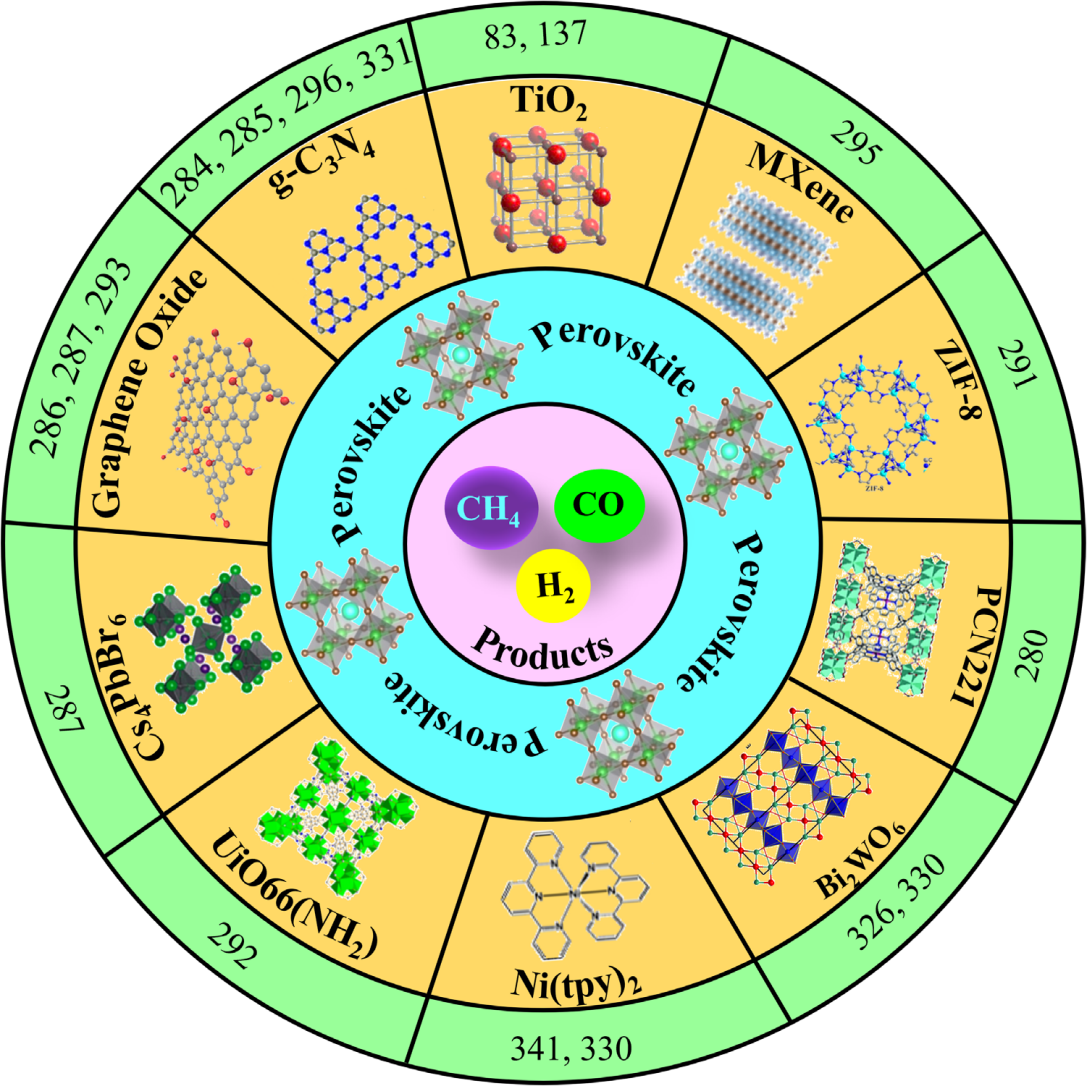
Halide perovskites combined with various other semiconductors and metal complexes for photocatalytic CO_2_ reduction. The related works can be found in these references: 1) refs. [83, 137], 2) ref. [292], 3) ref. [288], 4) ref. [279], 5) refs. [323, 327], 6) refs. [327, 337], 7) ref. [289], 8) ref. [284], 9) refs. [81, 284, 290], 10) refs. [124, 283, 293, 328].

All‐inorganic lead halide perovskites have been extensively explored in the photocatalytic application. In particular, they emerged as one of the most promising candidates for conversion of CO_2_ into value‐added chemicals and fuels in recent years due to their facile synthesis, tunable bandgap, and relatively higher stability than hybrid counterparts. In this regard, one of the early reports came from Hou et al.,^[^ ^282^ ^]^ in which they demonstrated the potentiality of colloidal CsPbBr_3_ QDs for photocatalytic reduction of CO_2_ with a yield of 20.9 µmol g^−1^ and a high selectivity greater than 99%. In addition, the high product yields of ≈4.3, 1.5, and 0.1 µmol g^−1^ h^−1^ achieved for CO, CH_4_, and H_2_, respectively. To enhance their photocatalytic efficiency, the inorganic perovskite NCs were combined with carbon nitride (g‐C_3_N_4_) through improved charge seperation.^[^ ^124^, ^283^ ^]^ Halide Perovskite combined with various other semiconductors and metal complexes for enhanced photocatalytic CO_2_ reduction are summarized in **Figure** **19**. The CsPbBr_3_‐g‐C_3_N_4_ composite showed improved stability for about 12 h with high yields of gaseous products (CH_4_ and CO) (≈300 µmol g^−1^) even at low concentrations of CO_2_ with a selectivity upto 91%. The photocatalytic activity of the composite was found to be ≈6‐fold and 4‐fold higher than that of pure g‐C_3_N_4_ and CsPbBr_3_, respectively. Similarly, the combination of perovskite NCs with GO has been significantly exploited for photocatalytic CO_2_ reduction. It was demonstrated that CsPbBr_3_ NCs/graphene oxide (GO) composite exhibits enhanced photocatalytic reduction of CO_2_.^[^ ^81^ ^]^ Notably, CsPbBr_3_ NCs/GO composite demonstrates an improved rate of the electron consumption from 23.7 to 29.8 µmol g^−1^ h^−1^ compared with individual CsPbBr_3_ QDs. The enhanced photocatalytic activity was attributed to electron extraction ability of conductive GO. Moreover, the CsPbBr_3_ QD/GO composite exhibits superior stability of 12 hours with a high amount of gaseous products, 58.7 and 29.6 µmol g^−1^ for CH_4_ and CO, respectively, with a selectivity of the product greater than 99%. Another potential nanocomposite systems for CO_2_ reduction was reported based on reduced‐GO (rGO) and zero‐dimentional (0D) Cs_4_PbBr_6_ perovskite.^[^ ^284^ ^]^ The combined composite demonstated a high rate CO production at 11.4 µmol g^−1^ h^−1^, maintaining long‐term stability for about 60 h and exhibited a high selectivity of 94.6%. This performance is nearly 6.2‐times higher then that of pristine Cs_4_PbBr_6_ perovskite. Similarly, the CO productivity of yield of 129 µmol g^−1^ was achieved for 10 hrs of visible light illumination using multi‐layer CsPbBr_3_ NCs/graphitic carbon nitride (CN)‐Titanium‐oxide (TiO) composite system.^[^ ^285^ ^]^ The production rate of CO is ≈3 and 6‐fold higher than pristine CsPbBr_3_ NCs and TiO‐CN nanosheets, respectively. The enhanced productivity was attributed to an increase of active sites and swift interfacial charge separation between CsPbBr_3_ and TiO‐CN. Likewise, multi‐layer CsPbBr_3_/g‐C_3_N_4_/thionyl bromide (SOBr_2_) composite also showed enhanced photocatalytic activity toward CO_2_ reduction.^[^ ^286^ ^]^ In this case, the SOBr_2_ does not only repair Br vacancies but also promotes the stripping of native ligands that effects charge transfer to nearby cocatalysts.^[^ ^287^ ^]^ The multilayer‐CsPbBr_3_/g‐C_3_N_4_/SOBr_2_ composite exhibits superior performance in reduction of CO_2_ into CO and CH_4_ evaluation with a high yield rate of 190 µmol g^−1^ h^−1^ under visible light irradiation, which is nearly 16 times higher than the pristine CsPbBr_3_. Besides, porous materials such as TiO_2_ have been used to extract the charge carriers and stabilize the NCs. The electron acceptor materials also can protect the NC surface in some cases. For instance, amorphous TiO_2_ (a‐TiO_2_) was used for encapsulation of CsPbBr_3_ NCs through a solution process.^[^ ^83^ ^]^ The encapsulated CsPbBr_3_ NC/a‐TiO_2_ composite exhibits ≈6.5‐fold higher photocatalytic activity toward CO_2_ reduction as compared with the pristine CsPbBr_3_ NC. Besides, CsPbBr_3_ NCs were combined with Cobalt/Zinc‐based zeolitic imidazolate framework (ZIF) through encapsulation and demonstrated a large enhancement of moisture stability, CO_2_ capturing ability, and charge separation efficiency.^[^ ^288^ ^]^ The electron consumption rate of CsPbBr_3_@ZIF for gaseous CO_2_ reduction is 2.66‐fold higher than that of pristine CsPbBr_3_. Another potential application of metal‐organic framework encapsulation has been reported by Wang and his co‐workers, where CsPbBr_3_ NCs/ UiO‐66(NH_2_) nanohybrid demonstrated considerable improvement in photocatalytic CO_2_ reduction under visible light irradiation than that of pristine CsPbBr_3_ QDs and UiO66(NH_2_).^[^ ^289^ ^]^ The improved photocatalytic performance was also attributed to the enhanced electron extraction and transfer between UiO66(NH_2_) and CsPbBr_3_ QDs, facilitated by the large accessible specific surface area and enhanced visible light absorption capacity of the nanojunction composite. Furthermore, CsPbBr_3_ NCs were loaded on hierarchical branched ZnO nanowire (BZNW) and microporous graphene scaffold (MRGO) to construct a multi‐layer device for fast charge transport and improved CO_2_ capture ability afforded by ZnO nanowire‐branched macroporous graphene.^[^ ^290^ ^]^ Due to the favorable synergistic effect, an enhanced photocatalytic performance was achieved with a photoelectron consumption rate of 52.02 µmolg_cat_^−1^ h^−1^, which is ≈4.98 and 1.65 times higher than that of CsPbBr_3_ NC (10.44 µmolg_cat_^−1^ h^−1^) and CsPbBr_3_ NC/MRGO (31.52 µmolg_cat_^−1^ h^−1^), respectively, with ≈96.7% selectivity of desirable CH_4_ production. Nanocomposites of certain types of materials can form Schottky‐junction at the interfaces. For example, Schottky‐junction photocatalyst was obtained by combining CsPbBr_3_ NCs with palladium nanosheet (Pd NS).^[^ ^291^ ^]^ In this composite system, the Pd NS acts as an electron reservoir to promptly separate the photogenerated electron−hole pairs of CsPbBr_3_ NC through a Schottky contact and thus provides an ideal site for CO_2_ reduction. The Schottky‐junction photocatalyst showed a high electron consumption rate of 33.79 µmol g^−1^ h^−1^, which is ≈2.43‐fold higher than that of pristine CsPbBr_3_ NCs (9.86 µmol g^−1^ h^−1^). Another interesting 2D material that has been exploited to enhance the charge separation and extraction in perovskite NCs is Mxenes. For instance, Pan et al. Demonstrated an In situ growth of uniform CsPbBr_3_ NCs in on the surface of two‐dimensional MXene nanosheets.^[^ ^292^ ^]^ The resulting nanocomposite exhibited enhanced charge seperation than in pristine CsPbBr_3_. The yields of the obtained CO and CH_4_ increased linearly with the light illumination time. The rate of the CO and CH_4_ evaluation are 26.32 and 7.25 µmol g^−1^ h^−1^ respectively, which are far higher than that of pristine CsPbBr_3_ NCs (<4.4 µmol/gh) as well as a few newly developed CsPbBr_3_ NCs‐based heterostructure photocatalysts.^[^ ^81^, ^282^, ^293^ ^]^ The bandgap of the CsPbX_3_ NCs is easily tunable by the halide ion composition, which enables the tunability of VB and CB energy levels according to the requirements. It has been shown that activity of mixed halide perovskites (CsPb(Cl_0.5_/Br_0.5_)_3_) exhibits photocatalytic activity toward CO_2_ reduction without using a photosensitizer, and maintains 99% selectivity toward CO and CH_4_ production with a total yield up to 875 µmol g^−1^.^[^ ^294^ ^]^ The yields in this case are far better than any other reported halide perovskites.^[^ ^81^, ^282^ ^]^ Moreover, the photocatalytic activity of the mixed halide perovskite toward CO_2_ reduction is ≈4.5‐ and 9.1‐fold higher than that of CsPbBr_3_ and CsPbCl_3_ under the same experimental conditions. The enhanced performance of mixed halide perovskites could be due to the better band alignment for efficient charge separation.

#### 4.3.1 Amino Acid‐Assisted Anchoring

To have a better charge transfer from perovskite NCs to acceptor materials, both need to be closely connected. Although physical adsorption of one material on top of other materials could lead to charge separation, linking the two systems through covalent functionalization can significantly increase the charge separation efficiency. For example, CsPbBr_3_ NCs were coupled to the surface of porous g‐C_3_N_4_ nanosheets by anchoring the amino group via N‐Br chemical bonding.^[^ ^293^ ^]^ The covalently linked composite showed superior stability and outstanding photocatalytic activity for CO_2_ reduction with a high CO production rate of 149 µmol/gh under visible light irradiation, which is almost 15 times higher than that of pristine CsPbBr_3_ NCs. The covalently‐bond composite exhibited superior stability and outstanding photocatalytic activity for CO_2_ reduction with a high production yield rate of 149 µmol/gh for CO evaluation under visible light irradiation, which is almost 15 times higher than that of pristine CsPbBr_3_ NCs. Despite the covalent binding between the two systems of photocatalytic nanocomposites, the incoherent interfaces caused by large lattice mismatches create high energy barriers for charge separation.^[^ ^295^ ^]^ Consequently, the catalytic activity of such composite systems can be significantly affected. To address this issue, Zhang et al.^[^ ^296^ ^]^ developed a semicoherent lattice‐matching composite by combining CsPbBr_3_ NCs and two‐dimensional (2D) PbSe. The photocatalytic activity of CsPbBr_3_/PbSe composite towards CO_2_ reduction showed selective CO evaluation with a rate of 322.4 µmol g^−1^ h^−1^, which is 3 times higher than that of pristine CsPbBr_3_ NCs. Besides, the photocatalytic activity of perovskite NCs and their stability is their surface defects (halide vacancies and uncoordinated Pb^2+^ atoms). It was shown that the addition of SO_4_^2−^ removes the uncoordinated Pb^2+^ ion from the surface of CsPbBr_3_ NCs through the formation of a water‐resistant PbSO_4_ product, and thus offers better stability in water. In this regard, Wang et al.^[^ ^297^ ^]^ found that the water‐stable CsPbBr_3_‐SO_4_ NCs exhibit photocatalytic CO_2_ reduction into CO and CH_4_ production. The CsPbBr_3_‐SO_4_ composite exhibits improved stability in water and high CO_2_ consumption with a rate of 56 µmol g^−1^h^−1^, which is nearly 8 times higher than that of pristine CsPbBr_3_.

#### 4.3.2 Metal Atoms Doping

Doping refers to the process of incorporation of a small percentage of hetero atom into a target lattice without significantly affecting the host crystal structure and basic characteristics. It is a general and effective approach for the modification of fundamental properties of semiconductors and improves their stability with a particular interest in optoelectronic application.^[^ ^298^ ^]^ Doping of metal ions in perovskites both the bulk and NCs have been extensively exploited to induce new optical features as well as enhance their stability and luminescence efficiency.^[^ ^298^, ^299^, ^300^, ^301^, ^302^ ^]^ However, the effect of doping on the photocatalytic performance has been relatively less explored. As discussed in above section, the selectivity of final product is very important but extremely challenging in photoctalytic CO_2_ reduction.^[^ ^303^ ^]^ Tang et al.^[^ ^304^ ^]^ theoretically predicted that Co and Fe‐doping in CsPbBr_3_ NCs lead to high selectivity of methane (CH_4_) production in photocatalytic CO_2_ reduction. The high selectivity was attributed to the decreased band gap of Co and Fe‐doped CsPbBr_3_ NCs, leading to broadened absorption toward visible light. Inspired by this report, furthermore, it was experimentally found that Fe(II) doped CsPbBr_3_ NCs exhibit high selectivity toward CH_4_ production instead of CO, while undoped CsPbBr_3_ NCs yield a mixture of CO and CH_4_.^[^ ^305^ ^]^ In another work, Kumar et al.^[^ ^306^ ^]^ investigated the photocatalytic ability of different morphologies of CsPbBr_3_ NCs for photocatalytic CO_2_ reduction and found that nanosheets produce CH_4_, CO, and H_2_ with a low yield of 0.43, 2.25, and 0.08 µmol g^−1^ h^−1^, respectively, however when the nanosheets combined with Cu‐loaded rGO (Cu‐rGO), the reaction yields CH_4_ with 98.5% selectivity and an yield of 12.7 µmol g^−1^ h^−1^. The improved activity, selectivity, and stability were attributed to efficient charge separation and the hydrophobic character of the composite. Similarly, Pb‐rich Mn and Ni‐doped CsPbCl_3_ NCs were also found to exhibit efficient photocatalytic ability toward CO_2_ reduction, where the Ni‐doped CsPbCl_3_ NCs exhibits higher product yields with a rate of 169.37 µmol g^−1^ h^−1^ in comparison to the rate of 152.49 µmol g^−1^ h^−1^ for Mn‐doped CsPbCl_3_ NCs under similar operating conditions.^[^ ^307^ ^]^ As discussed in the earlier section, the solvent plays an important role in the selectivity and efficiency of the product. In this regard, *Kuang* and co‐workers studied the effect of various non‐aqueous solvents such as toluene, ethyl acetate, isopropanol, acetonitrile, dimethyl formamide, and methanol on the photocatalytic CO_2_ reduction and found that the rate of product evolution was improved by 25 times when toluene was replaced with ethyl acetate.^[^ ^69^ ^]^ Moreover, the rate of CO_2_ consumption doubled when Pt co‐catalyst was photodeposited on CsPbBr_3_ NCs. Although the doping and introduction of co‐catalysts improve the efficiency of activity, the instability in water is a major challenge. A few studies reported that the stability of CsPbBr_3_ NCs can be greatly improved by forming a shell of Cs_4_PbBr_6_. This approach was also used to carry out the photocatalytic CO_2_ reduction in water using CsPbBr_3_/Cs_4_PbBr_6_‐composite photocatalysts.^[^ ^308^ ^]^ The whole catalytic reaction was carried out in pure water with an overall yield of CO and CH_4_ evaluation is 247 µmol g^−1^ after continuous irradiation of 20 h. The catalyst was also tested in acetonitrile and water medium and achieved a considerable enhancement of CO evolution with a yield of 1835 µmol g^−1^ under visible light continuous irradiation of 15 h.^[^ ^309^ ^]^ Furthermore, Mn‐doped CsPb(Cl/Br)_3_ mixed halide perovskite system was also tested for photocatalytic CO_2_ reduction and found that they produced CO and CH_4_ with optimum yields of 1917 and 82 µmol g^−1^ respectively, under visible light irradiation for about 9 h. These yields are significantly higher than those obtained using CsPbBr_3_ alone, by factors of approximately 14.2 and 1.4 times for CO and CH₄, respectively.^[^ ^310^ ^]^

#### 4.3.3 Pb‐Free All‐Inorganic Perovskites

Despite tremendous progress in lead halide perovskite‐based photocatalytic CO_2_ reduction, water oxidation, and organic transformation, their toxicity is a major concern for further use in real‐world applications.^[^ ^124^, ^259^, ^282^, ^290^, ^296^ ^]^ Therefore, researchers have been trying to find alternative halide perovskites with the same efficiency as Pb‐based counterparts. In this regard, Zhou et al.^[^ ^311^ ^]^ demonstrated the potentiality of Cs_2_AgBiBr_6_ double perovskite NCs as active materials for photocatalytic CO_2_ reduction into solar fuels. The double perovskite exhibits the total electron consumption of 105 µmol/g for evaluation of CO and CH_4_ after continues irradiation of 6 hours. In addition, 2D‐nanoplatelates of Cs_2_AgBiBr_6_ double perovskites were also synthesized and exploited in photocatalytic CO_2_ reduction into CO and CH_4_ selective products.^[^ ^127^ ^]^ During the course of the reaction time (about 6 h), the electron consumption of Cs_2_AgBiBr_6_ double perovskite nanoplates showed 8‐fold (255 µmol g^−1^) larger in comparison to the Cs_2_AgBiBr_6_ double perovskite NCs (30.8 µmol g^−1^). Furthermore, a series of non‐toxic zero‐dimentional (0D) A_3_Bi_2_I_9_ perovskite NCs (A = Rb^+^, Cs^+^, and CH_3_NH_3_^+^) were tested for photocatalytic reduction of CO_2_ into CO and CH_4_ at a gas‐solid interface approach, and the conversion mechanism was investigated by electron paramagnetic resonance and diffuse‐reflectance infrared spectra.^[^ ^312^ ^]^ The photocatalytic activity was found to be efficient for reduction of CO_2_ at the gas−solid interface with formation yields 14.9 µmol g^−1^ of methane and 77.6 µmol g^−1^ of CO, and indicating that significantly more effective catalyst than TiO_2_ under similar experimental conditions. Another type of 0D all‐inorganic Cs_3_Sb_2_Br_9_ NCs was also tested for CO_2_ reduction and found that only CO evolved with a rate of 510 µmol g^−1^ after 4 hours of continuous irradiation, which is ≈10‐folder higher than that of well‐established CsPbBr_3_ perovskite under similar conditions.^[^ ^313^ ^]^ A heterojunction is an interface between two layers of different semiconductors to suppress the rapid charge recombination and prolong their lifetime, as well as increases carrier density.^[^ ^314^, ^315^, ^316^, ^317^, ^318^ ^]^ However, initially, Wang et al.^[^ ^319^ ^]^ fabricated a heterojunction photocatalyst for CO_2_ reduction using 0D Cs_2_SnI_6_ NCs and 2D SnS_2_ chalcogenide nanosheet in an in situ process. The Cs_2_SnI_6_/SnS_2_ composite showed a prolonged photogenerated electron lifetime of 3080 picoseconds compared to its pristine SnS_2_ lifetime of 1290 picoseconds. Subsequently, a boosted photocatalytic activity was achieved, which is approximately 5.4 and 10.6‐fold higher than that of pristine Cs_2_SnI_6_ NCs and SnS_2_ nanosheet, respectively. In addition to the above, copper‐based Cs_2_CuBr_4_ QDs synthesized before loading on a mesoporous molecular sieve of KIT‐6 for a heterojunction photocatalyst to explore CO_2_ reduction.^[^ ^320^ ^]^The optimized Cs_2_CuBr_4_QDs/KIT‐6 showed noteworthy performance in the evaluation of CO and CH_4_ products with rates of 51.6 and 17.2 µmol g^−1^ h^−1^, respectively, indicating far exceeding activity than that of pristine Cs_2_CuBr_4_ QDs.

### 4.4 Z‐Scheme

**Figure 20:**
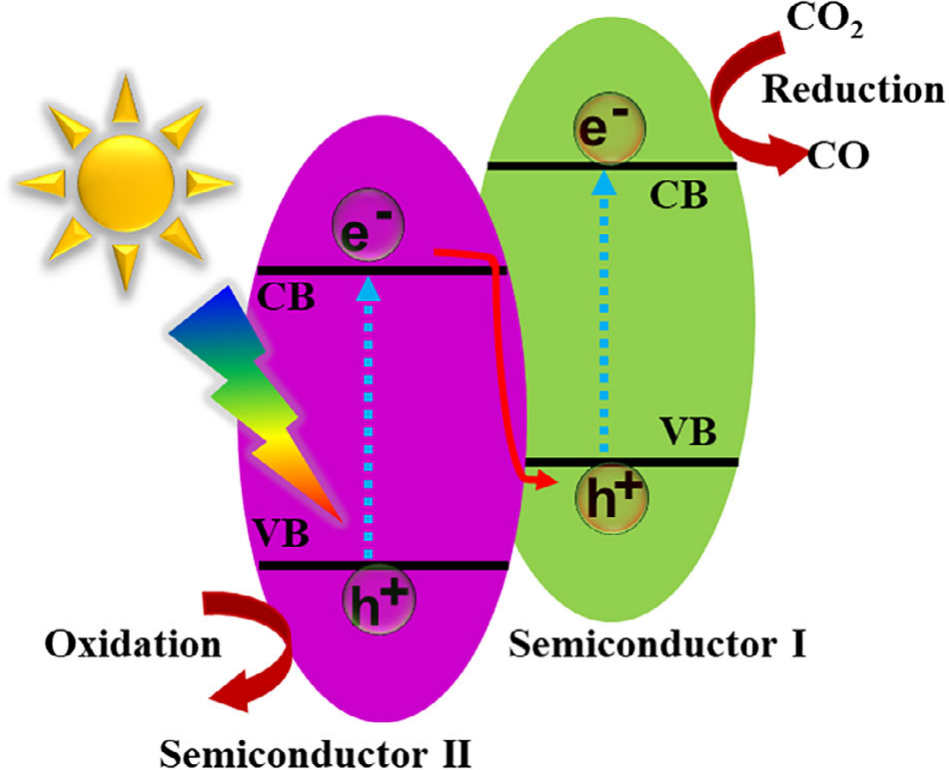
Schematic illustration of direct Z‐scheme heterojunction of photocatalyst for CO_2_ reduction.

Z‐scheme photocatalysis for CO₂ reduction offers a promising approach to convert CO₂ into valuable fuels and chemicals using solar energy. This photocatalyst operates in a similar manner to the photosynthesis process observed in nature. In a typical photosynthesis process, electrons are transferred between different components to facilitate energy conversion, whereas in the Z‐scheme composite, photogenerated electrons move between two photocatalysts with staggered band alignments, as shown in **Figure** **20**. By optimizing the interaction between the two photocatalysts and ensuring efficient charge transfer, the Z‐scheme system can significantly improve the overall photocatalytic performance. This is because the Z‐scheme photocatalyst effectively promotes light harvesting by utilizing two different photocatalysts, each absorbing different wavelengths of light and thereby broadening the range of the solar spectrum that can be utilized. Moreover, the operation of the Z‐scheme photocatalyst involves the following three key steps: i) it promotes light harvesting and maintains strong reducibility and oxidizability of reduction and oxidation of semiconductors, respectively. ii) the rapid charge transfer due to the Z‐scheme system significantly decreases the recombination of photoinduced electron‐hole pairs and then enhances the separation efficiency of effective charge carriers. iii) the separation of charge carriers can further promote the spatial separation of reductive and oxidative active sites, which ensures that specific photocatalytic reactions can occur in their corresponding active sites.^[^ ^321^, ^322^ ^]^

#### 4.4.1 Organic–Inorganic Hybrid Perovskites

Taking advantage of the Z‐scheme, Huang et al.^[^ ^323^ ^]^ developed a direct Z‐scheme photocatalyst for CO_2_ reduction using the combination of formamidinium lead bromide and bismuth tungstate (FAPbBr_3_/Bi_2_WO_6_) semiconductors with a strong redox ability. The reaction produced CO and benzaldehyde with an evolution rate of 170 µmol g^−1^ h^−1^ and 250 µmol g^−1^ h^−1^, respectively. The quantum yields of CO and benzaldehyde were determined to be 1.2% and 1.7% respectively, which are far larger in comparison to some of the reported perovskite‐based photocatalysts.^[^ ^324^, ^325^, ^326^ ^]^

#### 4.4.2 All‐Inorganic Perovskites

As discussed in earlier sections, pristine CsPbBr_3_ exhibits low catalytic performance mainly due to quick recombination and low carrier mobility of charges. Therefore, it was combined with other semiconductor materials to achieve Z‐scheme configuration and thus to improve the performance. In this regard, Wang et al.^[^ ^327^ ^]^ fabricated heterogeneous Z‐scheme photocatalyst by joining CsPbBr_3_ NCs and Bi_2_WO_6_ Nanosheets for efficient CO_2_ reduction. This Z‐scheme composite produced CO and CH_4_ with a total yield of 503 µmol g^−1^, which is nearly 9.5 times greater than that of pristine CsPbBr_3_ NCs during 10 h of reaction. The enhanced photocatalytic activity exhibits due to high spatial separation of photoexcited charge carriers, and strong individual redox abilities of two components. Likewise, another effective Z‐scheme photocatalyst for CO2 reduction was fabricated by selecting α‐Fe_2_O_3_ (2.2 eV) and CsPbBr_3_ NCs (2.3 eV) on account of their similar bandgap.^[^ ^324^ ^]^ The catalytic efficiency was further improved when combined with amine‐functionalized reduced graphene oxide (rGO), which helped to prolong the charge carrier lifetime.

#### 4.4.3 Pb‐Free All‐Inorganic Perovskites

The development of Pb‐free Z‐scheme photocatalysts represents a significant advancement in the field of photocatalytic CO₂ reduction. These systems offer enhanced stability, higher selectivity, and improved efficiency while being environmentally friendly. Wang et al.^[^ ^328^ ^]^ reported a Pb‐free Z‐scheme photocatalyst for a selective evaluation of CH_4_ by the reduction of CO_2_ using Cs_2_AgBiBr_6_ double perovskite and carbon nitride (g‐C_3_N_4_) combination. In this composite, g‐C_3_N_4_ conduction band acts as a reducing agent, and Cs_2_AgBiBr_6_ double perovskite valance band acts as an oxidizing agent in the catalytic reaction. The Pb‐free Z‐scheme photocatalyst exhibits excellent stability for about 4 h and the CH_4_ selectivity of over 70% with a rate of 2.0 µmol g^−1^ h^−1^_,_ which is 10‐fold and 16‐fold higher than that of pure g‐C_3_N_4_ and Cs_2_AgBiBr_6_ double perovskite, respectively. Another Pb‐free and eco‐friendly Z‐scheme photocatalyst was developed by decorating the ZnSe nanorods on the surface of CsSnCl_3_ NCs through an electrostatic self‐assembly approach.^[^ ^128^ ^]^ The ZnSe/CsSnCl_3_ composite produced CO and CH_4_ with a total rate of 57 µmol g^−1^ h^−1^, which is ≈5‐fold higher when compared to pristine ZnSe nanorods.

### 4.5 S‐Scheme Photocatalyst

The motivation behind the use of the S‐scheme heterojunction is aimed at overcoming the limitations of Z‐scheme photocatalysts, particularly the issue of low charge‐carrier separation efficiency.^[^ ^329^ ^]^ While both systems share similar charge transfer mechanisms, the S‐scheme was proposed as a refinement of the conventional Z‐scheme to provide a more precise depiction of charge transfer pathways. The key difference between these two systems lies in the electron‐hole pair generation mechanism. In Z‐scheme photocatalysts, electron‐hole pairs are generated sequentially through two different semiconductors connected in series. In contrast, S‐scheme photocatalysts generate electron‐hole pairs simultaneously through connected semiconductors in parallel. The crucial advantages of the S‐scheme include Fermi level alignment, work function, band alignment, band bending, and the generated interfacial electric field (E).^[^ ^329^, ^330^ ^]^ These aspects enable a more accurate representation of charge transfer pathways. Z‐scheme systems, however, do not account for these factors, often leading to an ambiguous depiction of charge transfer mechanisms.

Xu et al.^[^ ^137^ ^]^ developed a unique S‐scheme heterojunction by fabricating CsPbBr_3_ QDs onto mesoporous TiO_2_ nanofibers through an electrostatic self‐assembling approach for a boosting outcome in photochemical CO_2_ reduction. The S‐scheme TiO_2_/CsPbBr_3_ nanohybrid reduced the CO_2_ into solar fuels with a high rate of 9.02 µmol g^−1^ h^−1^, which is almost double than that of 4.68 µmol g^−1^ h^−1^ of pristine TiO_2_ under UV–vis irradiation. This high performance of S‐scheme composite is likely due to non‐aggregation of TiO_2_ nanofibers upon dispersion in solution, which fascinates to have high photocatalytic active sites for greatly encourage the separation of electron‐hole pairs in CO_2_ reduction. Furthermore, Dong et al.^[^ ^331^ ^]^ originated a S‐scheme heterojunction by incorporating CsPbBr_3_ NCs into a microporous Cu_2_O microsphere for exploration of CO_2_ reduction under visible light irradiation. Due to staggered bond alignment between CsPbBr_3_ and Cu_2_O, the Fermi level (*E*_F_) of Cu_2_O is much lower than that of CsPbBr_3_. Consequently, the S‐scheme composite acts as an efficient charge separator with a highly energetic electron and holes for excellent photocatalytic activity. Another novel S‐scheme photocatalyst of CsPbBr_3_ QDs and BiOBr nanosheets fabricated via a facile self‐assembly process.^[^ ^332^ ^]^ The resultant CsPbBr_3_/BiOBr composite delivered notable photocatalytic activity in CO_2_ reduction, with an electron consumption rate of 72.3 µmol g^−1^ h^−1^, which is almost 4.1 and 5.7 times higher when compared to the pristine CsPbBr_3_ NCs and BiOBr nanosheet, respectively.

#### 4.5.1 S‐Scheme with Pb‐Free Perovskites

In view of the high oscillator strength associated with Cu 3d states, Cs_2_CuBr_4_ perovskite nanodots grown in mesoporous CeO_2_ through a facile impregnation approach for a heterojunction S‐scheme have been applied for CO_2_ reduction.^[^ ^333^ ^]^ An outstanding photocatalytic activity was achieved with optimally loaded Cs_2_CuBr_4_/CeO_2_ composites, with yields of 271.56 and 83.28 µmol g^−1^ for CO and CH_4_ products, respectively. These results are almost 22.02 and 2.46 times larger than that of individual CeO_2_ and Cs_2_CuBr_4_, respectively.

### 4.6 Perovskites with Metal Complexes

Usually, metal complexes are superb candidates for CO_2_ reduction because of their tunable catalytic sites with high activity.^[^ ^334^, ^335^, ^336^ ^]^ But, most of the metal complexes have limits in absorbing visible‐light, which hinders their wide‐range applications in CO_2_ reduction.^[^ ^337^ ^]^ In view of this, metal complexes are coupled with organic or inorganic sensitizers to broaden their absorption range including visible‐light extent.^[^ ^338^, ^339^, ^340^ ^]^ Particularly, Chen et al.^[^ ^337^ ^]^ CsPbBr_3_ NCs coupled on the surface of Ni(tpy) (nickel complex with 2,2′:6′,2′′‐terpyridin) and used as a photocatalyst for CO_2_ reduction in visible‐light‐driven. The catalytic activity of coupled system showed a total yield of 1724 µmol g^−1^ for the evaluation of CO and CH_4_, which is ≈26‐fold higher than that achieved with the pristine CsPbBr_3_ NCs. Additionally, CsPbBr_3_ QDs also coupled on the surface of several transition‐metal chalcogenides (TMC; CdIn_2_S_4_, ZnIn_2_S_4_, and In_2_S_3_) in an interfacial contact, which enables uniform self‐assembly and boosted photoactivity toward CO_2_ conversion into CO compared to that of pristine CsPbBr_3_.^[^ ^341^ ^]^ Recently, Co‐ and Cu‐based metal complexes were coupled with lead‐free Cs₂AgBiBr₆ double perovskite^[^ ^342^ ^]^ and CsPbBr₃ perovskite,^[^ ^343^ ^]^ respectively, for photocatalytic CO₂ reduction. In this regard, Cs₂AgBiBr₆ nanosheets were anchored on cobalt tetraamino phthalocyanine (CoTAPc) and cobalt phthalocyanine tetracarboxylic acid (CoTCPc) for CO₂ photoreduction to CO, using water as the electron source. The Cs₂AgBiBr₆@CoTCPc hybrid photocatalyst exhibited the highest CO generation rate of 150 ± 6 µmol g^−1^ h^−1^ with a stoichiometric O₂ formation from water oxidation, which is over eight times higher than that of pure Cs₂AgBiBr₆ catalyst. It also showed a record‐high electron consumption rate of 300 ± 13 µmol g^−1^ h^−1^ among metal halide perovskite photocatalysts for CO₂ reduction. Experimental and computational studies highlight the critical role of carboxyl groups in anchoring Bi atoms, facilitating electron transfer between CoTCPc and Cs₂AgBiBr₆, thereby enhancing photocatalytic performance. In addition, ultrathin film devices were developed by leveraging synergistic effects from steric hindrance and electrostatic attraction for efficient CO₂ reduction with H₂O as the electron donor. These devices combine dinuclear‐metal molecular catalysts (DMC) and perovskite (PVK) quantum dots (QDs) as photosensitizers, integrated onto a conductive metal‐organic framework (cMOF), namely the copper cathelate of 2,3,6,7,10,11‐hexahydroxytriphenylene. The resulting [DMC@Cu‐CAT]‐PVK composite films produced CO with a rate of 133.36 µmol g^−1^ h^−1^, significantly surpassing the performance of PVK or DMC‐PVK alone. This enhanced photocatalytic activity is attributed to the precise spatial assembly of DMC and PVK together with the scalable fabrication of Cu‐CAT‐based film photocatalysts. In addition, a Zn‐phthalocyanine (ZnPc) complex was coupled with CsPbBr₃ perovskite in a heterojunction Z‐scheme process for photocatalytic CO₂ methanation with a CH₄ selectivity of up to 89% and an activity of 168 µmol g^−1^ h^−1^ for CH₄ production.^[^ ^108^ ^]^ This performance surpassed all previously reported perovskite‐based photocatalysts and was nearly 114 times higher than that of bare ZnPc.(**Figure** **20**)

### 4.7 Photoelectrocatalytic CO_2_ Reduction

**Figure 21:**
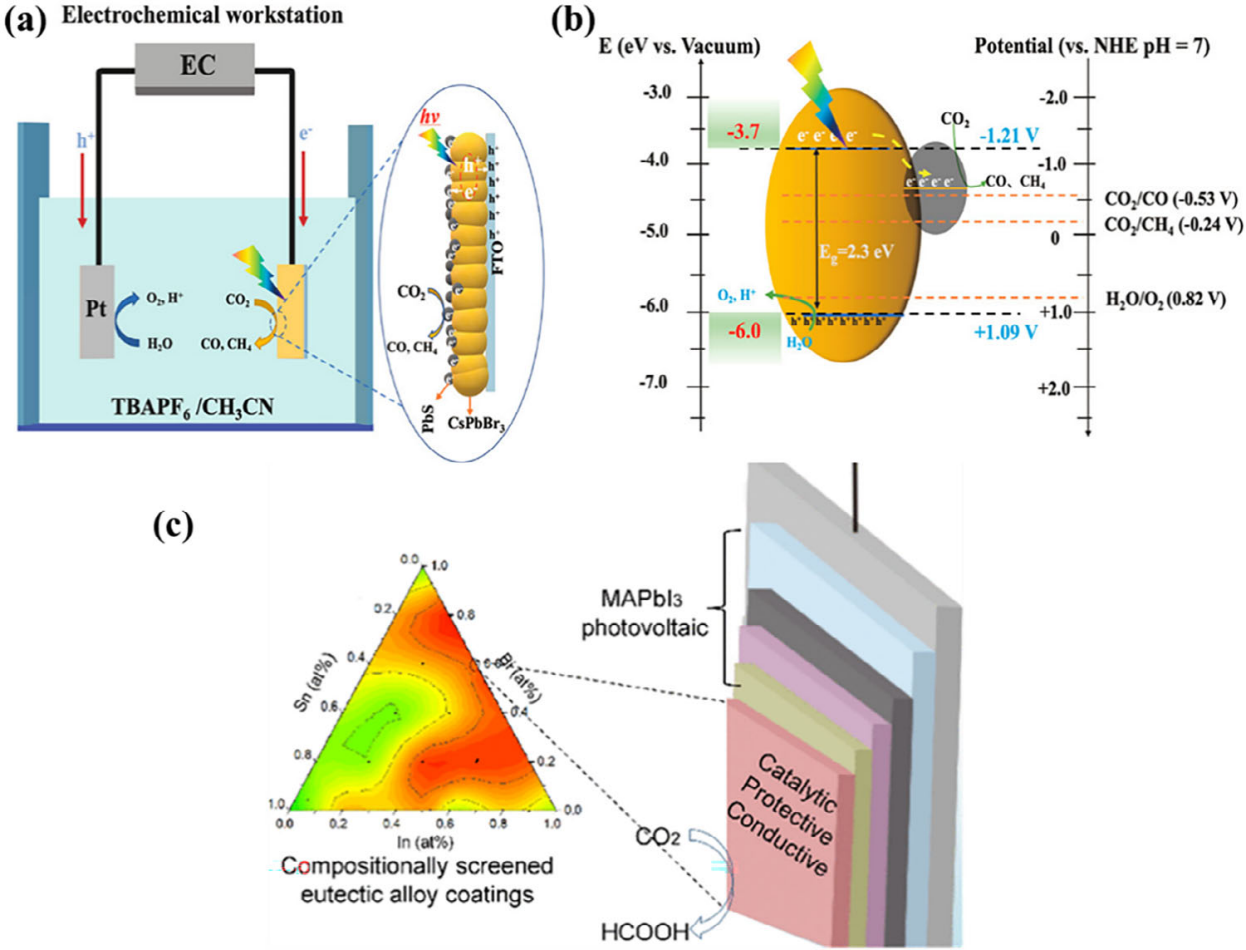
a) Schematic illustration of the photoelectrocatalytic CO_2_ reduction, b) energy‐band alignment of PbS/CsPbBr_3_‐12AA, and c) Schematic illustration of the compositional screening and photoelectrode structure of the catalytic alloy‐protected MAPbI_3_ photocathode for CO_2_ reduction into formic acid. (a) and (b) reproduced with permission from ref. [344]. Copyright 2022, American Chemical Society.

Reduction of CO_2_ through photoelectrocatalytic process using halide‐based perovskite has scarcely been reported.^[^ ^40^ ^]^ Encouragingly, for the first time, Chen et al.^[^ ^248^ ^]^ developed a photoelectrocatalyst using In‐Bi alloy coated MAPbI_3_‐based photocathodes for selective CO_2_ reduction into formic acid production in aqueous solution with nearly 100% faradaic efficiency. The photoelectrocatalyst operated at −0.6 V versus RHE under simulated AM 1.5G irradiation for more than 1.5 h and achieved a photo‐assisted electrolysis system efficiency of 7.2%. Addition to above, recently, heterojunction photoelectrocatalyst greatly fabricated PbS nanocrystals anchoring on the surface of CsPbBr_3_ with the help of 12‐aminododecanoic acid.^[^ ^344^ ^]^ Here, the anchoring is likely possible due to direct interaction between −COO^−^ (−NH_3_^+^) and Pb^2+^ (Br^−^). However, photoelectrocatalytic activity of CsPbBr_3_‐PbS exhibits approximately 2.4 times higher than that of pristine CsPbBr_3_ NCs in a selective reduction of CO_2_ into evaluation of CO and CH_4_ with a rate of 2.94 and 0.36 µmol cm^−2^ h^−1^, respectively (**Figure** **21**).

## 5 Photocatalytic Organic Reactions Using Colloidal Nanocrystal Catalysts

Recent advances in visible light photocatalysis have greatly expanded the synthetic protocols available to chemists. In photocatalysis, photon absorption by transition metal complexes, organic dyes, or semiconductors facilitates these materials to attain an electronically excited state. This state can take part in charge transfer interactions with organic molecules, enabling various redox processes that open up new pathways for organic synthesis. Although homogeneous photocatalysts are widely used, their recovery and reuse after reactions are challenging, often requiring extra purification steps to separate the photocatalyst from the product. In contrast, heterogeneous photocatalysts such as metal oxide semiconductors, perovskite semiconductors, and graphitic carbon nitrides offer the advantage of easy recyclability due to their solid phase, making them a more sustainable and cost‐effective choice for photocatalytic applications.^[^ ^346^, ^347^, ^348^ ^]^

Over the years, colloidal nanocrystals (NCs) of different materials have been exploited as heterogeneous catalysts for chemical transformations. They offer unique properties compared to their bulk counterparts, such as a high surface‐to‐volume ratio and thus more active sites, tunable composition, size, and shape. In this context, perovskite NCs have demonstrated their promise through their ability to achieve high Photocurrent Conversion Efficiency (PCE), due to their high absorption coefficients and outstanding optoelectronic properties, as previously mentioned. As discussed above, MHPs have high stability in organic solvents and are thus expected to be highly suitable for organic transformations. Motivated by this, we here discuss the recent literature to showcase the different chemical transformations employing MHP NC catalysts. Surprisingly, MHPs have been employed in a diverse range of reactions, such as C‐C, C‐N, and C‐O coupling, cyclization, ring‐opening reactions, polymerization, aromatization, decarboxylation reactions, asymmetric synthesis, and more (**Table** **4**). A comprehensive review of these varied reactions necessitates an in‐depth understanding of the essential factors at play. This discussion is instrumental in developing highly efficient photocatalytic methods in chemistry that yield selective product outcomes.

### 5.1 Charge and Energy Extraction: NC‐Chromophore Composites

**Figure 22:**
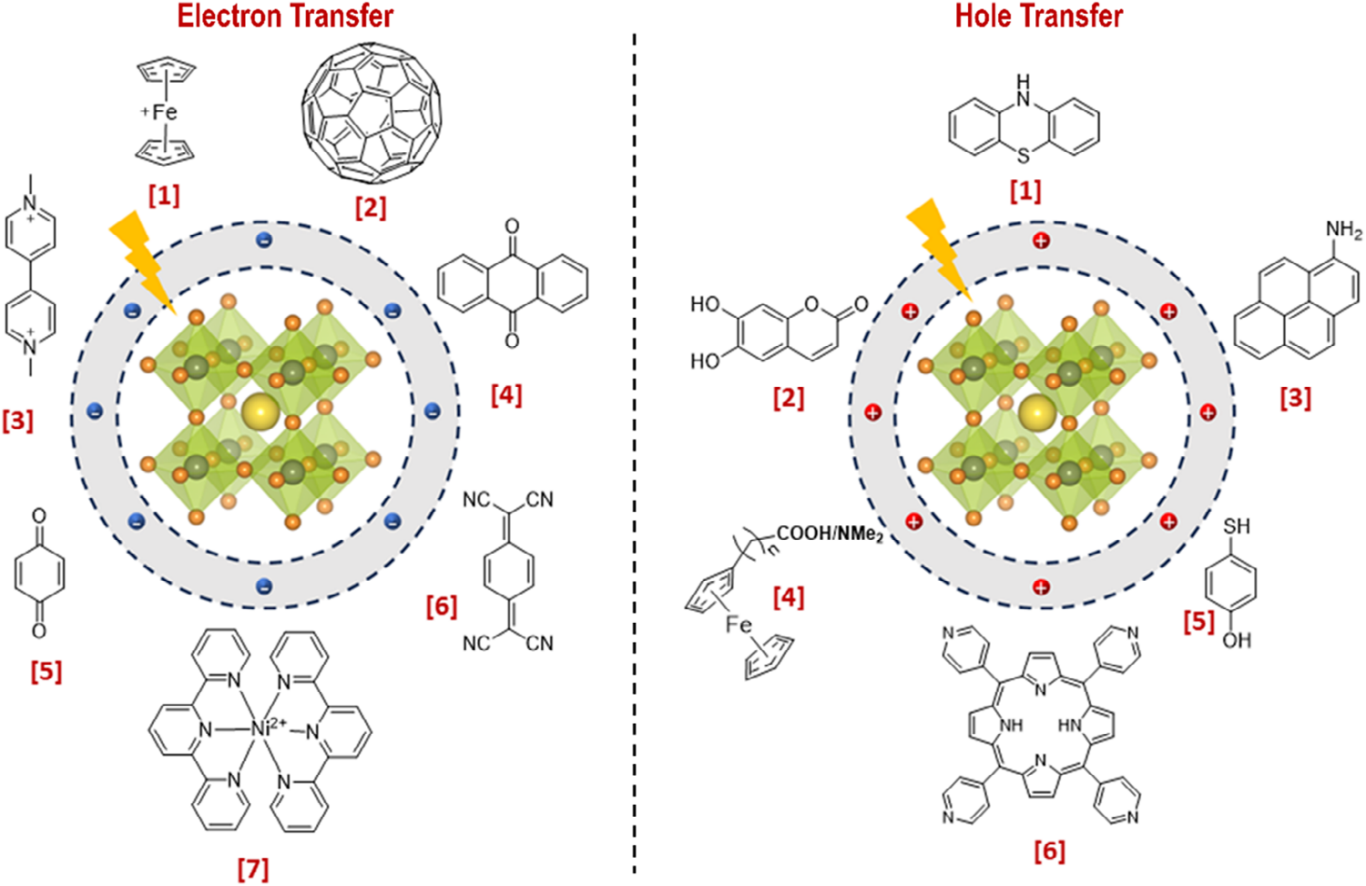
Summary of the molecules utilized for selectively extracting charge carriers (Either electron or hole) from MHPs. The molecules are chosen from references^[^ ^337^, ^375^, ^376^, ^377^, ^378^, ^379^ ^]^ for electron transfer and references^[^ ^42^, ^380^, ^381^, ^382^, ^383^, ^384^, ^385^ ^]^for hole transfer.

Despite exhibiting strong interaction with light and near‐unity PLQY,^[^ ^31^ ^]^ MHP's primary obstacle resides in their incapacity to attain optimum photoconversion efficiency. This limitation stems from the lack of readily available charge carriers on their surface for catalytic processes, unlike metal NCs. To tackle this issue, employing small molecules capable of channeling energy and extracting charge carriers to the catalytic site becomes imperative for facilitating reactions. Interestingly, this has been achieved by anchoring MHP NCs with a variety of molecules capable of selectively extracting electrons and holes, such as methyl viologen, fullerene, benzoquinone, ferrocene, coumarin derivatives, and many more (**Figure** **22**).^[^ ^42^, ^337^, ^375^, ^376^, ^377^, ^378^, ^379^, ^380^, ^381^, ^382^, ^383^, ^384^, ^385^ ^]^ These molecules must exhibit energetically favorable alignment with the perovskite and strong interaction with the NC surface, which can be achieved either through anchoring groups, electrostatic interactions, or physisorption.^[^ ^386^ ^]^ As of now, the highest reported association constant, K_app_, is approximately 10^8^ M^−1^.^[^ ^387^ ^]^ This was achieved using ‐COOH functionalized ferrocene on the surface of CsPbBr_3_ NCs, enabling the extraction of holes in significant quantities and within a femtosecond timescale. On the other hand, methyl viologen exhibits a K_app_ around 10^6^ M^−1^ with CsPbBr_3_, demonstrating its high capacity to extract electrons in substantial amounts to the nanocrystal surface.^[^ ^376^, ^388^ ^]^ These numerical values pave the way for hole and electron‐driven chemical transformations, maximizing the turnover number (TON) when utilizing these composites as a photocatalyst. For example, Jin et al. showed how the photoexcitation of CsPbBr_3_ NCs led to the polymerization of trithiocarbonate (TTC) ligands on their surface.^[^ ^389^ ^]^ The polymerization reaction was initiated by the transfer of charges from perovskite NCs to the TTC ligands anchored on the surface.

An alternative approach for extracting charge carriers from semiconductors includes transferring energy to an acceptor molecule. Upon photoexcitation, this process facilitates the transfer of the semiconductor's excitons to an acceptor molecule, thereby generating an excited state in the acceptor. Numerous studies have documented singlet and triplet energy transfer phenomena between excited MHPs and acceptor molecules. Singlet energy transfer to an acceptor requires the singlet energy level (S_0_‐S_1_) to be lower than the semiconductor's bandgap. Whereas to generate a triplet excited state, which is typically spin‐forbidden upon direct excitation, a chromophore in the S_1_ state can undergo intersystem crossing or singlet fission. Alternatively, a triplet excited state can be produced through direct energy transfer from an excited semiconductor, provided that the sensitizer's band gap is lower than the acceptor's singlet energy level but higher than its triplet energy level.^[^ ^386^ ^]^ Moreover, under specific conditions where the triplet energy is only 100–200 meV less than the exciton energy, the triplet excited states can repopulate the sensitizer's excited state via thermally activated reverse triplet energy transfer (rTET), thereby extending its lifetime significantly.^[^ ^390^ ^]^

**Figure 23:**
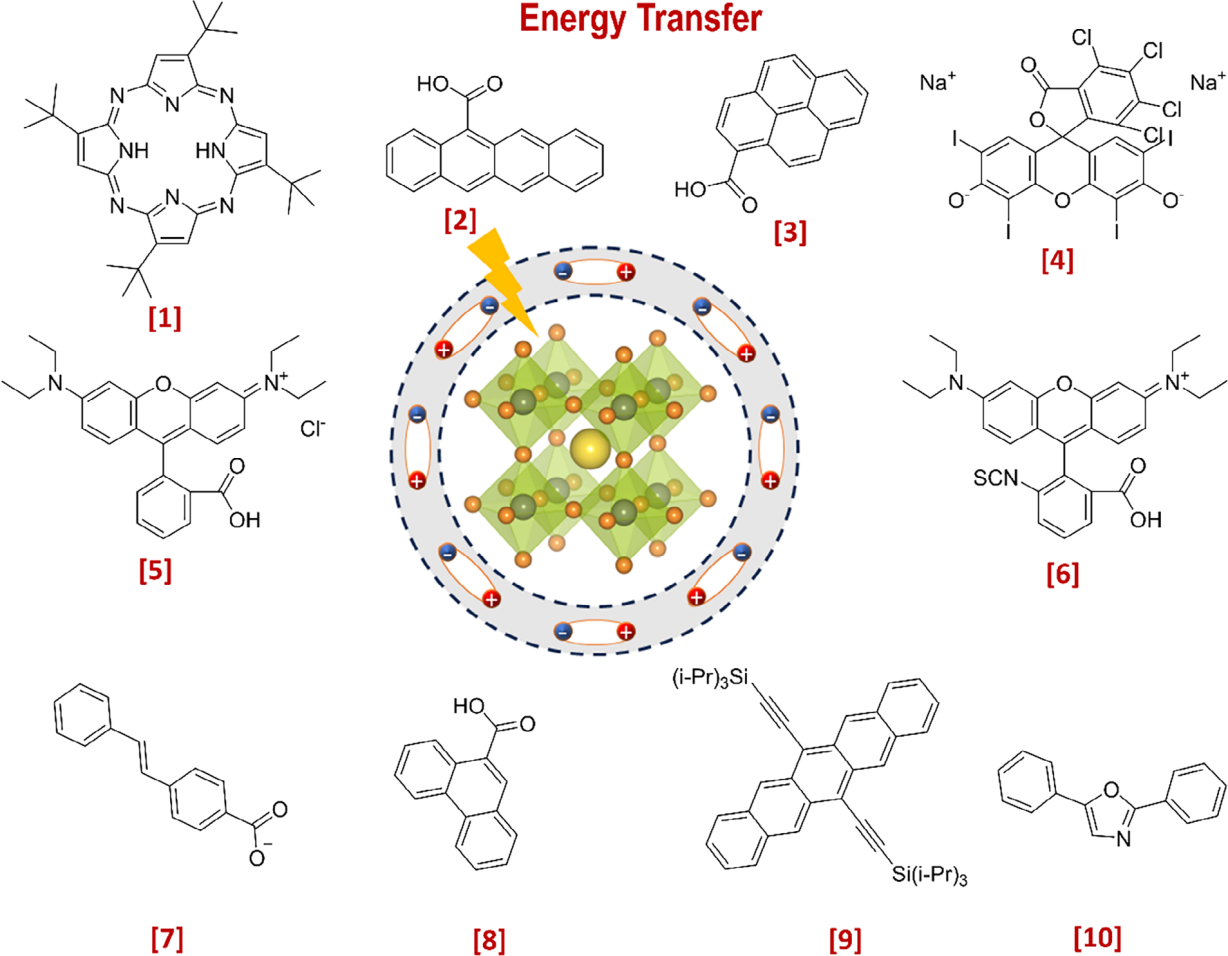
Summary of the molecules utilized for extracting charge carriers in the form of electron‐hole pairs or demonstrated to show energy transfer from MHPs. The molecules are chosen from the references.^[^ ^391^, ^392^, ^393^, ^394^, ^395^, ^396^, ^397^, ^398^ ^]^

Various works have demonstrated the excitation of acceptor chromophores via energy transfer from MHP NCs. A wide range of chromophores including porphyrazine, rhodamine derivatives, rose bengal, pentacene, and 9‐phenanthrene carboxylic acid, among others have been studied (**Figure** **23**).^[^ ^391^, ^392^, ^393^, ^394^, ^395^, ^396^, ^397^, ^398^ ^]^ These indirectly excited chromophores could find applications in photocatalysis by extracting the charge carriers and thus facilitating their availability on the catalyst's surface for a longer duration. The lifetime of charge carriers in MHPs can reach several microseconds when complexed with energy acceptors; however, without these acceptors, the lifetime is limited to just a few nanoseconds. For example, He et al. reported triplet energy transfer (TET) from CsPbBr_3_ NCs to 9‐phenanthrene carboxylic acid (PTA).^[^ ^390^ ^]^ Subsequently, the excited triplet states repopulated the NCs' excited state through reverse triplet energy transfer (rTET), resulting in an enhanced lifetime of 80 *µ*s. This composite system was then used for steady‐state photoreduction of anthraquinone, which was not achievable using only CsPbBr_3_ NCs. In addition, direct ET has been utilized for chemical transformations such as [2+2] cycloaddition, and cis‐trans isomerization, highlighting recent advancements.^[^ ^363^, ^397^ ^]^

### 5.2 Approaches for MHP‐Photocatalyzed Reactions

#### 5.2.1 O_2_ Activation Approach

**Figure 24:**
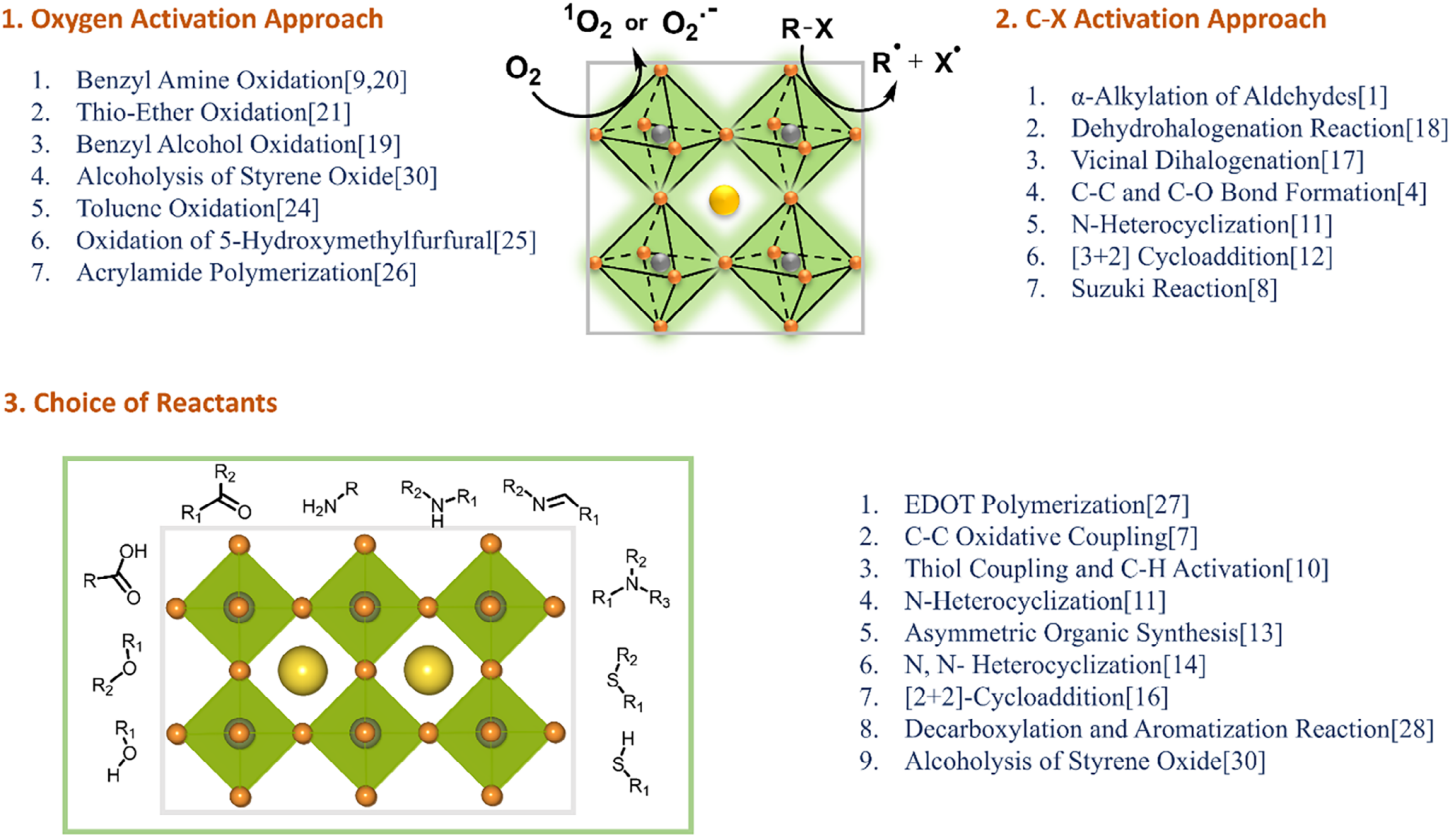
Diagram illustrating three approaches that have been employed to showcase photocatalytic chemical transformations catalyzed by Metal Halide Perovskites (MHPs). [] represents the entry of the corresponding reaction in Table 3.

Oxygen is often deemed an excellent oxidant due to its widespread availability, lightweight nature, and tendency to produce water as a harmless by‐product in many instances. The growing emphasis on eco‐friendly and sustainable synthetic techniques has sparked significant interest in utilizing O_2_ as a reagent in synthesis processes.^[^ ^399^ ^]^ In photocatalysis, two types of oxidation reactions are known to employ O_2_ as an oxidant. In type I, photoexcited holes take part in oxidation, while O_2_ accepts electrons to regenerate the catalyst without incorporating into the product. The example of type I includes dehydrogenation of alcohols to form aldehydes and ketones are such types of reactions employing MHPs as photocatalysts.^[^ ^366^, ^370^ ^]^ Conversely, Type II reactions involve the incorporation of one or both oxygen atoms from O_2_ into the final product. These reactions typically follow a radical pathway, with oxygen being assimilated after transformation into superoxide radicals by utilizing photoinduced electrons. Such reactions were also widely reported using MHP NC photocatalysts. These include the oxidation of toluene, thioether, benzylamine, etc.^[^ ^356^, ^357^, ^368^, ^369^ ^]^ Based on an in‐depth review of the literature, it appears that O_2_ strongly interacts with MHP surfaces and is readily reducible due to favorable energetics (0.33 eV versus RHE).^[^ ^46^ ^]^ As a result, it has been extensively utilized in both the types of reactions (**Figure** **24**).

#### 5.2.2 C–X Activation Approach

**Figure 25:**
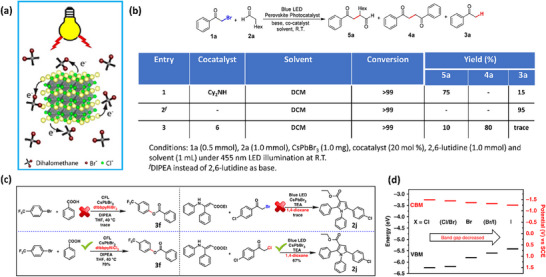
a) Proposed mechanism of photoinduced halide exchange reaction. Reproduced with permission.^[^ ^400^ ^]^ Copyright 2017, American Chemical Society b) The chemical process (C‐C coupling) and the tabulated data illustrating varied yields under distinct conditions. Reproduced with permission.^[^ ^349^ ^]^ Copyright 2019, American Chemical Society c) Two instances of effective reactions showcasing the tuning of perovskite band characteristics. d) Variation of band characteristics with different halide contents. Reproduced with permission.^[^ ^29^ ^]^ Copyright 2019, Nature.

Another aspect of MHPs‐based photocatalysis involves the activation of the C–X bond, originating from a photoinduced halide exchange reaction, where the bond can be broken simply by irradiating a white or blue light (**Figure** **25**a). Subsequently, the generated halide (X) exchanges with the intrinsic halides of MHPs (X' of ABX'_3_). Using dichloromethane as the reactant, this idea was first demonstrated by Parobek et al., later Wong and coworkers found that this exchange process is not limited to perovskite only, whereas this also brought a chemical change in C–X containing substrates.^[^ ^400^, ^401^ ^]^ The suggested mechanism indicates that the C–X interacts with MHPs because of halide vacancies on the surface. These vacancies allow halides to coordinate with metal cations (B), leading to subsequent activation.^[^ ^402^ ^]^ Furthermore, this key concept was utilized in many organic reactions such as C‐C coupling, N‐hetero‐cyclization, C‐O cross‐coupling, dehydrohalogenation, halogenation reactions, etc.^[^ ^46^ ^]^(Figure 24).

#### 5.2.3 Choice of Reactants

Another method used in MHPs‐based photocatalysis is the choice of reactants containing functional groups or atoms that are prone to the perovskite surface. Carboxylic acid and amine groups are known to passivate the MHP surface,^[^ ^130^, ^261^ ^]^ and thus the introduction of these groups to the reactants facilitates the organic transformations. For example, the presence of ─COOH group in the substrate enabled a highly selective [2+2]‐cycloaddition, while interactions with amines were utilized for N‐hetero‐cyclization and benzylamine oxidation.^[^ ^29^, ^357^, ^363^ ^]^ Additionally, sulfur and oxygen atoms can also interact with MHP surface through their Lewis basicity based on the HSAB principle.^[^ ^403^, ^404^, ^405^ ^]^ This could be seen in the organic transformations such as EDOT polymerization, thiol coupling, sulfide oxidation, and alcoholysis of styrene oxide, etc.^[^ ^356^, ^358^, ^371^, ^374^ ^]^ Alkyl phosphonic acids and phosphines are also known for the strong passivation of the MHP surface.^[^ ^130^ ^]^ In a correlation, the phosphorous‐containing substrates were also noted in a phosphorylation reaction where a dehydrogenative coupling was demonstrated between tertiary amines and phosphite esters.^[^ ^358^ ^]^ Thus the substrate choice could be made based on the existing passivating strategies to achieve efficient photocatalysis employing MHPs (Figure 24).

### 5.3 C‐C, C‐N, and C‐O Bond Formation

In this regard, Yan‘s group in 2019, has taken phenacyl bromide and octanal as substrates, with CsPbBr_3_ NCs as a photocatalyst to achieve various products via C‐C coupling.^[^ ^349^ ^]^ Different products were achieved by varying the co‐catalysts, and sacrificial donors in the reaction mixture. Introducing *N*,*N*‐di isopropyl‐ethylamine (DIPEA) without any cocatalyst resulted in a highly selective conversion to acetophenone (**3a**) whereas replacing amine with **6**, (5S)‐(−)‐2,2,3‐trimethyl 5‐benzyl‐4‐imidazolidinone resulted in sp^3^‐C coupling product (**4a**) with 80% yield. Additionally, the presence of dicyclohexylamine and 2,6‐lutidine produces (**5a)** with 75% yield by activating C‐Br and C‐H bond in a single catalytic cycle according to the provided mechanism. (Figure 25b) The investigated mechanism suggested that all the products were formed following the key step of C‐Br activation (phenacyl bromide) via photoinduced electron transfer from perovskite NCs. Phenacyl radicals were trapped using TEMPO, isolated, and analyzed using NMR. Another report from the same research group employed not only C‐Br activation as a pivotal step but also activated the comparatively stronger C‐Cl bond to showcase C‐C coupling, N‐hetero‐cyclization, and C‐O cross‐coupling reactions by using other cocatalysts and reagents.^[^ ^29^ ^]^ This report highlights a correlation between nanocrystal size, reaction rate, and product yield. It was observed that smaller‐size NCs facilitated a faster reaction rate, albeit without achieving a high yield in a longer reaction time (6h). Conversely, larger‐sized NCs led to a higher yield but with a slower reaction rate. The explanation lies in the fact that the larger surface‐to‐volume ratio of smaller NCs facilitates higher rates, while the detrimental effects due to moisture and light irradiation are more pronounced in these NCs than in larger NCs. Another intriguing aspect is the band tuning of halide perovskite NCs, which broadens the range of substrates that can be activated by facilitating their thermodynamic feasibility. As presented in Figure 25c, **3** **f** is formed in trace amounts when CsPbBr_3_ NCs acts as photosensitizer and dtbbpyNiBr_2_ as cocatalyst, while replacement of cocatalyst with dtbbpyNiCl_2_ resulted in 78% of yield. This observation was cross‐confirmed by replacing CsPbBr_3_ NCs with an Ir‐based photo sensitizer where no such effect was detected. A similar kind of observation was drawn in another reaction (**2j)** shown in Figure 25c, where replacing *α*‐bromoketone with *α*‐chloroketone resulted in an enhancement in yield to 67%. These observations were not only anticipated but also validated through various controls, confirming their existence due to an in‐situ halide exchange. This exchange leads to a shift in the conduction and valence bands, consequently resulting in enhanced reaction yield. (Figure 25d)

**Figure 26:**
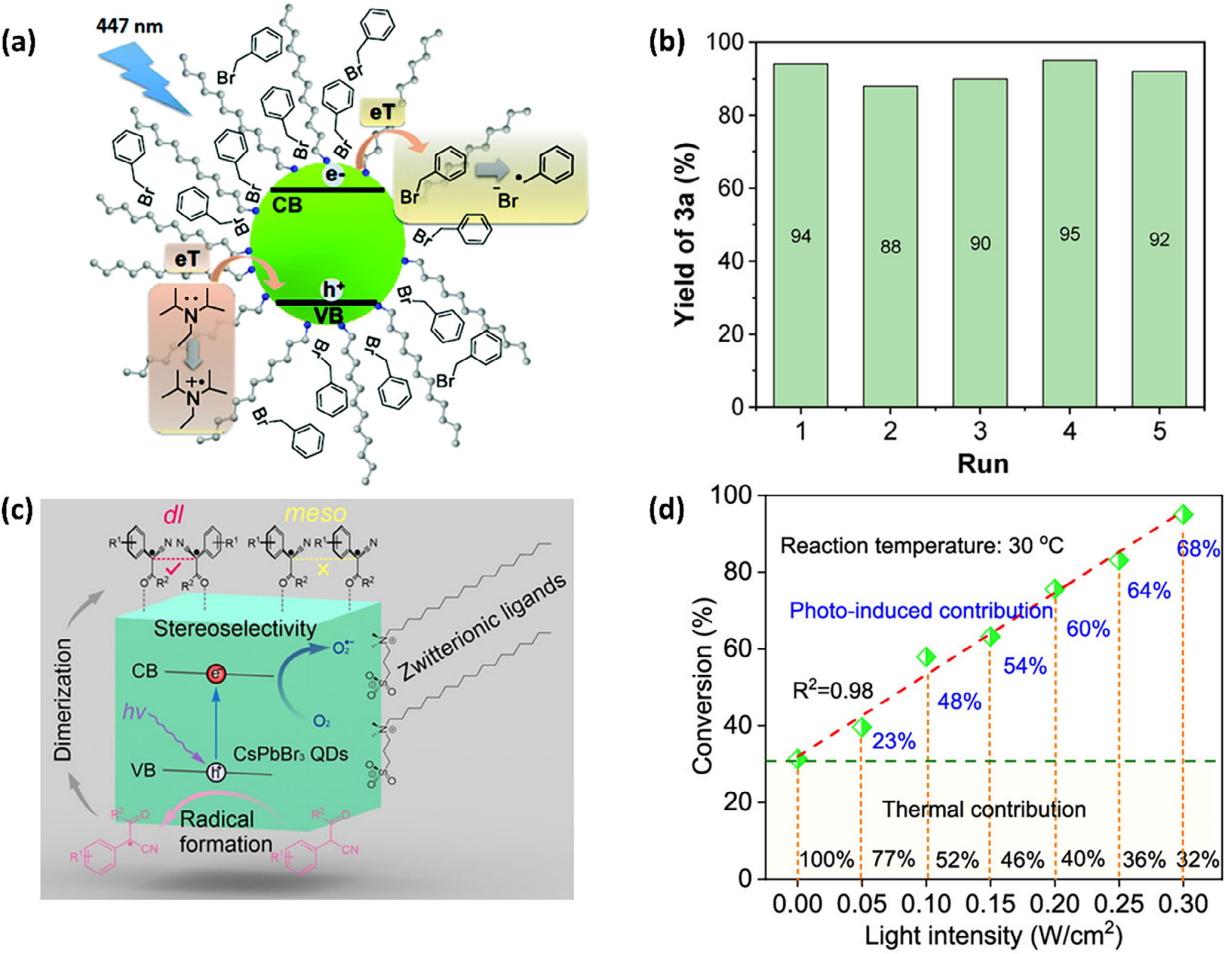
a) The proposed path of electron and hole transfer after light excitation of the CsPbBr_3_ in the presence of the benzyl halide and DIPEA encapsulated within the lipophilic shell. Reproduced with permission.^[^ ^350^ ^]^ Copyright 2020, The Royal Society of Chemistry.  b) Recyclability test of the catalyst up to 5 cycles for CsPbBr_3_ catalyzed aminomethylation of Imidazo fused heterocycles. Reproduced with permission.^[^ ^351^ ^]^ Copyright 2020, Wiley‐VCH. c) Suggested reaction path for photocatalytic stereoselective oxidative dimerization of α‐aryl ketonitriles catalyzed by zwitterionic ligands capped lead halide perovskites. Reproduced with permission.^[^ ^354^ ^]^ Copyright 2020, Wiley‐VCH. d) The catalytic activity of Pd/CsPbBr_3_ for the Suzuki coupling reaction at varying solar light intensities when maintained at 30 °C for 4 h. Reproduced with permission.^[^ ^355^ ^]^ Copyright 2022, American Chemical Society.

Rosa‐Pardo and coworkers have reported lipophilic ligand‐capped CsPbBr_3_ NCs, concentrating benzyl bromide and tertiary amine (electron donor) to the catalyst surface due to van der waals interactions (**Figure** **26**a).^[^ ^350^ ^]^ This strategy resulted in the facile activation of the C‐Br bond of benzyl bromide, which is otherwise thermodynamically unfavorable. This led to the homo‐/cross‐coupling of benzyl bromide with a turnover number of up to 17500. Recently, Shi et al have recognized the recyclability of the photocatalysts as one of the major issues and paved their effort to demonstrate CsPbBr_3_ catalyzed decarboxylative aminomethylation of imidazo‐fused heterocycles.^[^ ^351^ ^]^ For this reaction, CsPbBr_3_ was recycled and used five times without any significant reduction in the reaction yield. (Figure 26b) Mechanistic studies have revealed that hole‐driven decarboxylation is followed by the formation of an amino methyl radical, which subsequently interacts with imidazo‐fused heterocycles to produce an amino‐methylated product.

In 2020, Yuan and colleagues demonstrated the stereoselective C‐C oxidative dimerization of α‐aryl keto nitriles using zwitterionic ligand‐stabilized CsPbBr_3_ NCs as the photocatalyst.^[^ ^354^ ^]^ The requirement for zwitterionic ligand arose because of stability issues encountered with the recycling of the catalyst capped with oleic acid/oleyl amine‐coated NCs. The zwitterionic ligand‐stabilized NCs not only exhibited recyclability for up to three cycles but also led to an increased reaction rate, attributed to the reduced density of surface ligand coverage. Furthermore, a high stereoselectivity (>99%) was observed favoring *dl‐* isomers over *meso‐*isomers in cases involving NCs, which was attributed to reduced steric hindrance between the two benzene rings of the substrates when the substrate interacts with the NCs' surface in a *trans*‐orientation (Figure 26c). Wang et al. showcased an intriguing study where they synthesized a CsPbBr_3_/Pd composite to enhance Suzuki coupling (coupling between aryl halides and phenylboronic acid).^[^ ^355^ ^]^ Whereas for pure CsPbBr_3_, the conversion and selectivity was 11% and 81%, respectively, but the introduction of 3% wt. Pd leads to 96.5% conversion with a selectivity of nearly 100%. To rationalize the thermal effect, the temperature was fixed at 30 °C and the reactions were performed at different light intensities including dark. A linear increase in the conversion with light intensity was observed in Figure 26d, suggesting the key role of light in the reaction. Finally, the stability test of the catalyst has revealed no significant decline in activity even after six cycles. Moreover, the integrity of the crystal structure and the presence of deposited Pd were confirmed through XRD and ICP‐MS analyses.

### 5.4 S‐S and C‐P Bond Formation

Disulfides play a crucial role in facilitating the folding of proteins into biologically active structures and have extensive applications in the fine chemical industry. The known methods utilize typical oxidants for the oxidative coupling of thiols, which can lead to overoxidation and have challenges with selectivity in the synthesis of unsymmetrical disulfides. To tackle these, Wu and coworkers have utilized CsPbBr_3_ NC catalysts to couple thiols in a symmetrical as well as in an unsymmetrical manner.^[^ ^358^ ^]^ The thiol binding to the nanocrystal surface was verified through the photoluminescence spectrum, showing a gradual increase in intensity attributed to Pb‐S binding, which was further confirmed using FTIR spectroscopy. The reaction yield significantly improved in the presence of oxygen, reaching up to 98%. In contrast, under oxygen‐free conditions, the yield was notably reduced to 65%. This establishes that oxygen accepts electrons and turn into superoxide, while holes participate in oxidizing thiols into thiyl radicals, which then couple with another radical to form disulfides. In the absence of oxygen, thiol protons accept electrons and release hydrogen gas as a byproduct. Moreover, the uniqueness of NC catalysts was explored through a reaction conducted with bulk CsPbBr_3_, resulting in a significantly decreased yield of <8%, indicating the necessity of a large surface area for efficient catalytic activity. Furthermore, during the synthesis of unsymmetrical disulfides, noticeable selectivity was observed, with a 3:1 ratio of unsymmetrical to symmetrical products. In the same article, phosphorylation of tertiary amines using CsPbBr_3_ NCs was also demonstrated, achieving a maximum yield of up to 98%. Additionally, the confirmation of substrate (tetrahydroisoquinoline and phosphite) interaction with the catalytic surface was established by the enhancement in photoluminescence when exposing the substrates to the catalyst individually. The involvement of oxygen in the reaction was deemed significant, as photo‐generated superoxide was proposed to extract protons from the radical cation intermediate and transform into an α‐amino radical. C‐P coupling reaction serves as a significant synthetic approach due to the considerable structural complexity found in nitrogen‐containing pharmaceutical compounds.

### 5.5 Cyclization Reactions

**Figure 27:**
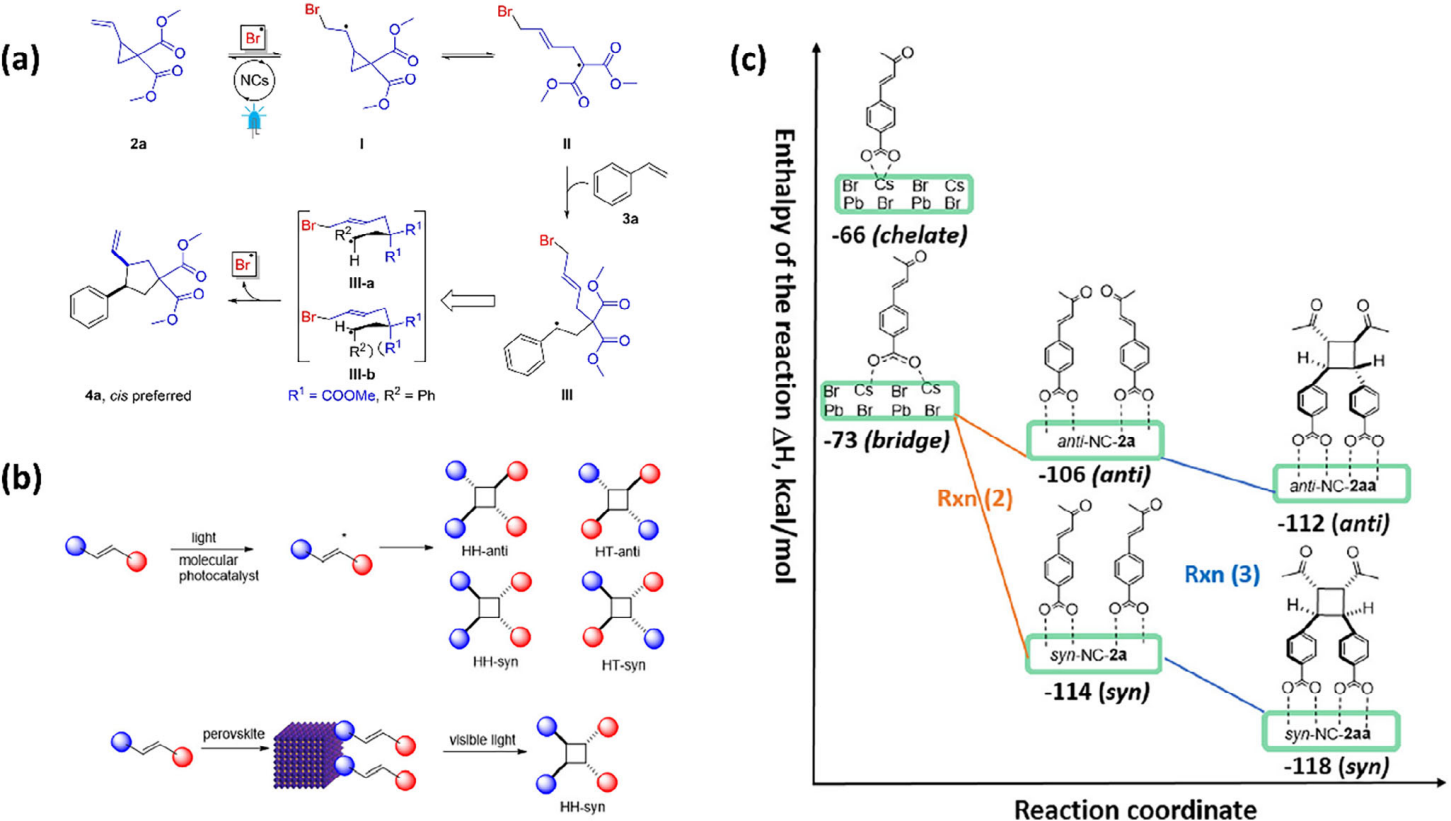
a) Proposed pathway for the [3+2] cycloaddition catalyzed by nanocrystals via bromine mediation. Reprinted with permission.^[^ ^359^ ^]^ Copyright 2023, The Authors. ChemSusChem published by Wiley‐VCH GmbH. b) Schematic of [2+2]‐ cycloaddition in the presence and absence of perovskite NCs, showcasing how substrate binding dictates the formation of diastereomeric *syn*‐selective product. c) Binding enthalpies of the intermediates involved in the reaction. Reprinted with permission.^[^ ^363^ ^]^ Copyright 2022, American Chemical Society.

As established above, dichloro/dibromo‐methane is susceptible to perovskite for photoinduced halide exchange reaction, which results in the formation of chloride/bromide radical as an intermediate.^[^ ^400^ ^]^ The Yan group^[^ ^359^ ^]^ employed a co‐catalyst strategy in which the utilization of in‐situ generated bromine radicals derived from the oxidation of bromide ions (photogenerated via a single electron transfer to the C‐Br bond) for [3+2]‐cycloaddition. Following the mechanism in **Figure** **27**a, the bromine radical first corroborated with molecule **2a** which subsequently reacted with alkene **3a** to form benzyl‐based radical **III**. Ultimately, the release of a bromine radical led to the formation of the final product **4a**. Interestingly, the reaction was diastereoselective and resulted in cis‐**4a** as a major product, which was confirmed by nuclear Overhauser effect spectroscopy (NOSEY NMR). This selectivity was attributed to the formation of transition state **III‐a**, which lacks 1,3‐diaxial interaction and is thus more stable than **III‐b**. Regarding recyclability, CsPbBr_3_ NCs have shown no decrement in their activity up to 3 cycles, attributed to a self‐healing process to the presence of excess bromine in the system.

In another report, the Yan group has introduced an innovative approach by employing triplet energy transfer (TET) with reactant molecules having a ─COOH anchoring group. This has been employed to demonstrate a remarkable diastereomeric *syn*‐selective [2+2] cycloaddition by utilizing lead halide perovskites as a triplet energy donor.^[^ ^363^ ^]^ As shown in Figure 27b, employing a molecular triplet energy donor generates triplet states in unbound reactant molecules, resulting in the formation of all possible products. In contrast, when using MHPs as donors (CsPbBr_3_), the ‐COOH groups on the reactants anchor them to the perovskite surface, which enforces selective product formation and promotes syn addition. The scanning of functional groups indicated that the substrates containing ─COOH with appropriate energy alignment exhibited the TET, while other substrates remained inert. As expected, TET‐activated molecules have shown successful [2+2]‐cycloaddition with yields up to 64%. To understand the diastereomeric selectivity, Density Functional Theory (DFT) studies were performed. In the free state, the reaction was exothermic for both *anti‐* and *syn*‐mode with ∆H_rxn_ of −8 and −6 kcal/mole, respectively, resulting in an *anti‐*selective product. Whereas in the bound state, *syn*‐mode binding is preferred, having lower energy than *anti*‐mode, resulting in a *syn*‐selective product (Figure 27c). Here, the strong ‐COOH binding was attributed to the positively charged Cs‐rich CsPbBr_3_ NC surface.

**Figure 28:**
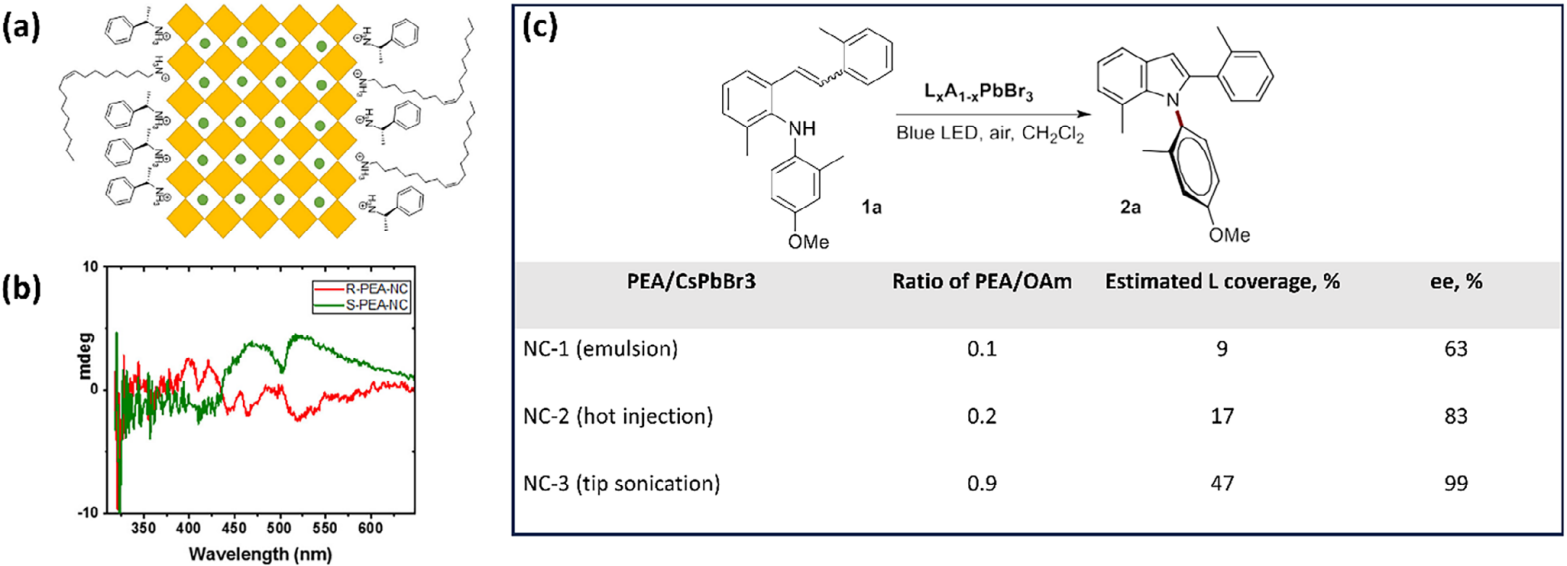
a) The proposed structure of chiral perovskite NCs formulated with L_2_(APbBr_3_)_n‐1_ PbBr_4_ with ‘n’ approaching infinity. b) Circular dichroism spectra of *R*‐NC‐3 and *S*‐NC‐3. c) The synthesis of chiral perovskites using various methods and their respective performance in the reaction. Reproduced with permission.^[^ ^360^ ^]^ Copyright 2023, American Chemical Society.

Another report from the Yan group has demonstrated an asymmetric synthesis using chiral CsPbBr_3_ NCs through amine‐based surface interactions.^[^ ^360^ ^]^ First, to formulate chiral perovskite NCs (L_2_(APbBr_3_) _n‐1_PbBr_4_, where n = ∞, L = chiral ligand and A = Cs^+^, **Figure** **28**a), diverse methods were tried and ultimately employed the tip sonication. Through this method maximum coverage (≈47%) of the chiral ligand (chiral 1‐phenylethylamine) on the perovskite surface was achieved. These perovskites, denoted as NC‐3, exhibited a characteristic signal at 515 nm in circular dichroism (CD) spectra (Figure 28b), indicating the establishment of electronic coupling between the chiral ligand and the perovskite nanocrystal lattice. The NC catalysts were further employed to control the chirality of N‐hetero‐cyclization reactions by inducing prochirality in substrate **1a** (Figure 28c). Consistent with the hypothesis, the photocatalytic outcomes indicated that increased chiral‐surface coverage leads to higher enantioselectivity, as shown in the table provided in Figure 28c. According to HPLC data, the nanocrystals synthesized using the tip sonication method (NC‐3) exhibited an enantiomeric excess (*ee*) of over 99% for product **2a**. In elucidating the mechanism, the atroposelective formation of N‐aryl indole was ascribed to simultaneous hole transfer during the ring‐closing step. This association was substantiated through DFT, revealing a subtle hydrogen bond between the N‐arylamine substrate and the chiral organic site (1‐phenylethylammonium) on the surface. During the recycling and regeneration of the catalyst, the enantiomeric excess (*ee*) of product **2a** gradually declined from 99% to 90% after four cycles, likely due to the reduction of chiral ligand surface coverage.

**Figure 29:**
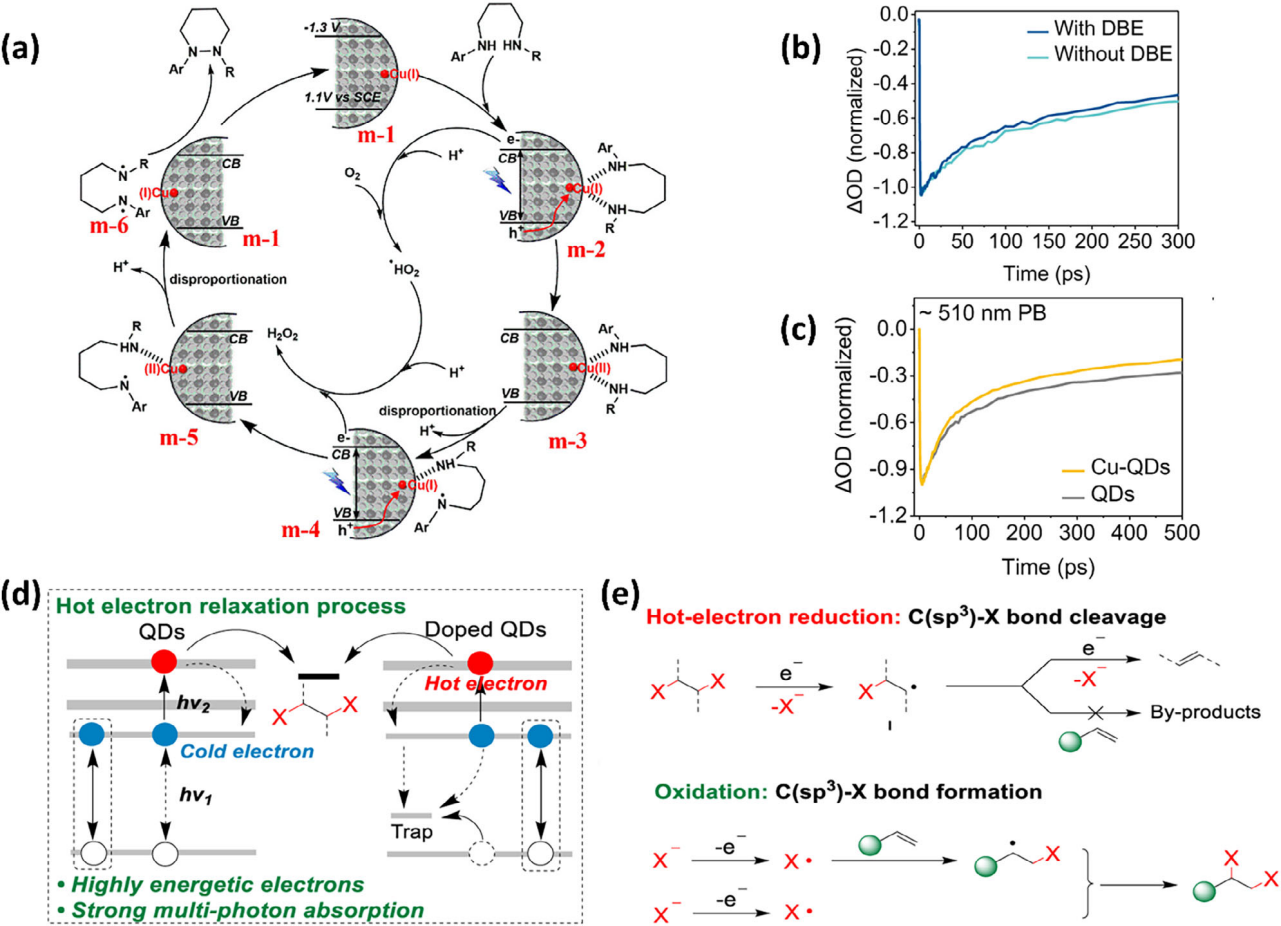
a) Proposed mechanism for intramolecular *N‐N* hetero‐cyclization by using copper‐doped CsPbBr_3_ as a photocatalyst. Reprinted with permission.^[^ ^361^ ^]^ Copyright 2021, American Chemical Society. b) Transient absorption (TA) kinetics of CsPbBr_3_ NCs at 510 nm in presence and absence of 1,2‐dibromoethane (DBE). c) Comparison of TA kinetics of CsPbBr_3_ NCs and copper‐doped CsPbBr_3_ NCs at 510 nm. d) Energy requirements for photocatalytic shuttle reactions. e) Photocatalytic steps showcasing reduction and oxidation processes involved in vicinal di‐halogenation reaction. Reproduced with permission.^[^ ^364^ ^]^ Copyright 2023, The Author(s). Nature.

Furthermore, Intramolecular *N‐N* hetero‐cyclization was accomplished by Martin et al. via multiple hole transfer from copper (I)‐doped CsPbBr_3_ (Cu: CPB) to the diamine substrates.^[^ ^361^ ^]^ The introduction of copper into the nanocrystals was achieved through post‐synthetic cation exchange with cesium, employing CuBr. Upon suspending Cu: CPB NCs in dichloromethane together with substrate, pyridazine was produced with a 90% yield under aerobic conditions under 450 nm LED illumination. Mechanistic investigation revealed the catalytic role of copper, where the Cu(I)/ Cu(II) redox couple played a significant role in achieving the multiple‐hole transfer via capturing the hole generated upon photoexcitation(**Figure** **29**a). The confirmation of Cu(II) formation was observed through EPR spectroscopy, recorded under both light‐on and –off conditions. Moreover, inhibited conversion in the absence of oxygen indicated that the photoexcited electrons were efficiently quenched by oxygen, thereby regenerating the catalyst. The N−N heterocyclizations demonstrated significant success in synthesizing pyridazines and 5‐membered pyrazolidines, involving 52 substrates.

### 5.6 Dehydrohalogenation and Halogenation Reactions

In the search for dehydrohalogenation reaction protocols under mild conditions, Singh *et. al* used CsPbBr_3_ NCs as a catalyst‐cum‐reactant, and tetrachloroethane as a halogen‐containing substrate.^[^ ^365^ ^]^ Exposure to white light led to the generation of a mixed halide perovskite (CsPbCl_x_Br_3‐x_), serving as an effective photo‐catalyst for the reaction until its transformation into CsPbCl_3_ has occurred. The mechanism suggested the key step of the reaction is the activation of the C‐Cl bond to form carbon radical which further releases a proton to quench the hole present on the photocatalyst, resulting in the formation of trichloroethylene.

The synthesis of two neighboring carbon‐halogen bonds in a molecule is synthetically significant. Li et al. reported vicinal dibromination and dichlorination of alkenes using CsPbBr_3_ and copper (Cu)‐doped CsPbBr_3_ NCs as photocatalysts where halide sources were taken as dibromoethane (DBE) and tetrachloroethane (TCE).^[^ ^364^ ^]^ White light irradiation initiates the generation of two photon‐mediated hot charge carriers, aligning with the energy requirements for the reduction of DBE and TCE. The confirmation of the participation of hot charge carriers in the reaction was established by recording transient absorption spectra of CsPbBr_3_ NCs, revealing accelerated bleach recovery in the presence of DBE (Figure 29b). Interestingly, using pristine CsPbBr_3_ NCs, dichlorination could not be achieved with a high yield due to a difficulty in the extraction of the second chlorine from the TCE. To tackle this, copper was doped to slow down the relaxation of hot electrons, which were then available for a longer time to facilitate reduction. This was assured again by comparing the bleach recovery profile of copper‐doped and pristine CsPbBr_3_ NCs as shown in Figure 29c. The faster recovery was attributed to the accelerated non‐radiative recombination as the copper captured the holes. This process resulted in prolonged availability of electrons at the 1Se level for a longer time. Subsequently, these electrons were further excited to higher energy levels by absorbing a second photon, facilitating reduction (Figure 29d,e).

### 5.7 Oxidation Reactions

**Figure 30:**
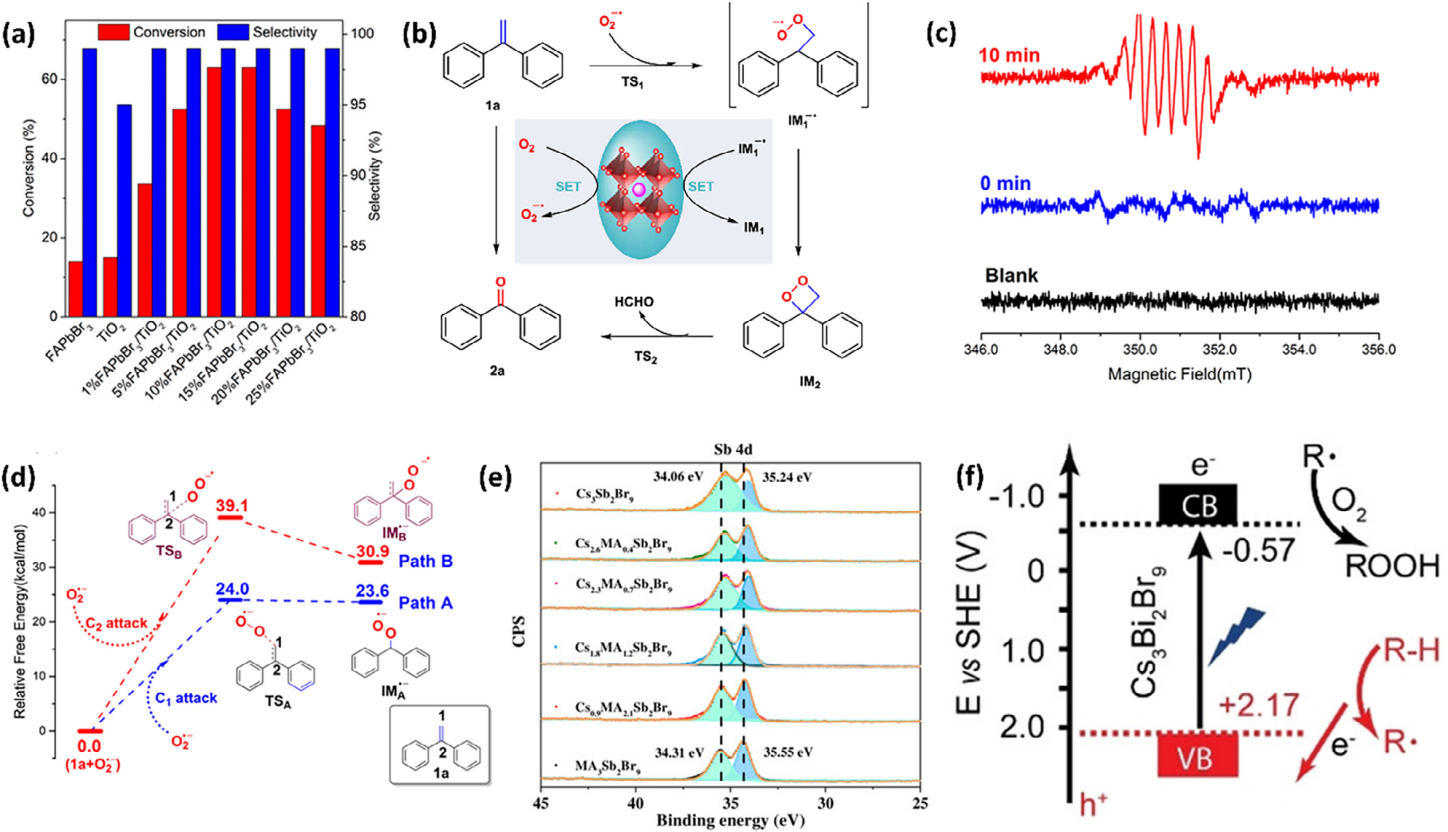
a) Conversion and selectivity obtained in benzyl alcohol oxidation by loading different amounts of FAPbBr_3_ over TiO_2_. Reprinted with permission.^[^ ^366^ ^]^ Copyright 2018, American Chemical Society. b) The suggested mechanism for the oxidative cleavage of C═C bonds to produce carbonyl compounds. c) EPR spectra for detecting superoxide radicals. d) Gibbs energy diagram illustrating two pathways for the incorporation of superoxide, as determined through DFT. Reprinted with permission.^[^ ^367^ ^]^ Copyright 2023, American Chemical Society. e) X‐ray Photoelectron Spectroscopy (XPS) spectra of Sb 4d for nanoparticles (NPs) of Sb‐based halide perovskites with varying Cs and MA (Cs*_x_* MA_3−_ *_x_* Sb_2_Br_9_) composition (*x* = 0–3). Reprinted with permission.^[^ ^368^ ^]^ Copyright 2020 Wiley‐VCH GmbH. f) The band positions of Cs_3_Bi_2_Br_9_ nanoparticles and their plausible catalytic mechanism as a photocatalyst for oxidizing hydrocarbons under visible light in the presence of air. Reprinted with permission.^[^ ^369^ ^]^ Copyright 2019, The Authors. Published by Wiley‐VCH.

Given the significance of oxidation reactions in synthetic organic chemistry for introducing or transforming functional groups, it's imperative to delve into catalytic reactions using MHPs in this field. Huang and coworkers have demonstrated the oxidation of benzyl alcohols to benzaldehyde by using a hybrid of TiO_2_ with FAPbBr_3_.^[^ ^366^ ^]^ As such, the conversion was found to be enhanced up to 4x in comparison with the pure substances (FAPbBr_3_ or TiO_2_) whereas the selectivity of benzaldehyde differs from 99% to 95% (**Figure** **30**a). In another report, Dong et al. achieved 100% selective conversion to benzaldehyde using CsPbBr_3_/Polyoxometalate composites (CsPbBr_3_/Ni_4_P_2_).^[^ ^109^ ^]^ The combination of steady‐state and time‐resolved photoluminescence (PL) revealed the enhanced dissociation of the exciton into free charge carriers, which was attributed to the optimum conversion obtained with the composite. Mechanistic studies revealed that the TiO_2_/Ni_4_P_2_ served as a location for reduction (from O_2_ to O_2_^−^), while FAPbI_3_/CsPbBr_3_ acted as an oxidation site (from RCH_2_OH to RCH_2_OH*), ultimately resulting in the formation of the oxidation product.

In addition, Fan and coworkers have utilized CsPbBr_3_ NC catalysts for the oxidation of amines and sulfides in aerobic and non‐aerobic conditions with high selectivity (>99%) and conversion (>99%).^[^ ^356^ ^]^ The products, imines, and sulfoxides have attracted considerable attention because of their crucial role as valuable components in the production of fine chemicals and bioactive substances. Interestingly, the electron‐donating group containing benzylic amines has resulted in the formation of aldehydes as major products, whereas the electron‐withdrawing group promoted the formation of imines as a major constituent. In the oxidation process, oxygen was identified as the crucial element in sulfide oxidation, otherwise the conversion was significantly suppressed in its absence. Conversely, the conversion of amine to imine did not show any such effect. Mechanistic studies established the crucial role of superoxide radicals through scavenging with 1,4‐benzoquinone, and of holes with potassium iodide. Intriguingly, both the oxidations get inhibited in the presence of singlet oxygen (generated via energy transfer) scavenger 1,4‐diazabicyclo[2.2.2]octane (DABCO), suggesting the involvement of both the energy and single electron transfer (ET and SET) in the reaction.

Given the formation of reactive oxygen species (ROS) upon photoexcitation of CsPbBr_3_, Fan et al. have utilized the in‐situ generated superoxide and singlet oxygen to facilitate the cleavage of C = C bonds, leading to the production of carbonyl compounds under aerobic conditions.^[^ ^367^ ^]^ This approach resulted in a 95% yield of benzophenone from 1,1‐stilbene under standard optimized conditions. The investigated mechanism provided in Figure 30b, which clarifies the role of oxygen in activating the olefin forming [2+2]‐adduct (IM_2_) via SETs. The EPR spectrum in Figure 30c displayed the presence of the superoxide radical intermediate. Concurrently, DFT studies supported the formation of IM1, revealing a low energy barrier for the addition of superoxide to C1 (Figure 30d).

Furthermore, the oxidation of toluene to benzaldehyde was demonstrated using lead‐free perovskites A_3_Sb_2_Br_9_ and Cs_3_Bi_2_Br_9_.^[^ ^368^, ^369^ ^]^ Zhang et al. in their report highlighted the role of A‐site cation in lead‐free A_3_Sb_2_Br_3_ perovskite, transforming toluene to benzaldehyde by hole‐driven C‐H activation.^[^ ^368^ ^]^ In the context of Cs_x_MA_1‐x_SbBr_9_, XPS investigations presented in Figure 30e indicated that increasing the value of x led to a noticeable decrease in the binding energy of Sb^2+^. This observation implies the creation of Sb with a relatively higher electron density, consequently generating sites on Br that are electron deficient. Further, these electron‐deficient sites promoted the enhanced rate of C‐H activation and thus the corresponding product formation. The study by Dai and colleagues investigated the oxidation of toluene using Cs_3_Bi_2_Br_9_ as a photocatalyst.^[^ ^369^ ^]^ In a control experiment conducted under anaerobic conditions, the reaction yielded 1,2‐diphenyl ethane instead of benzaldehyde and benzyl alcohol, underscoring the essential role of O_2_ in the conversion process (Figure 30f). Furthermore, control experiments revealed a significant decrease in yield when using hole and radical scavenging reagents.

### 5.8 Polymerization Reactions

**Figure 31:**
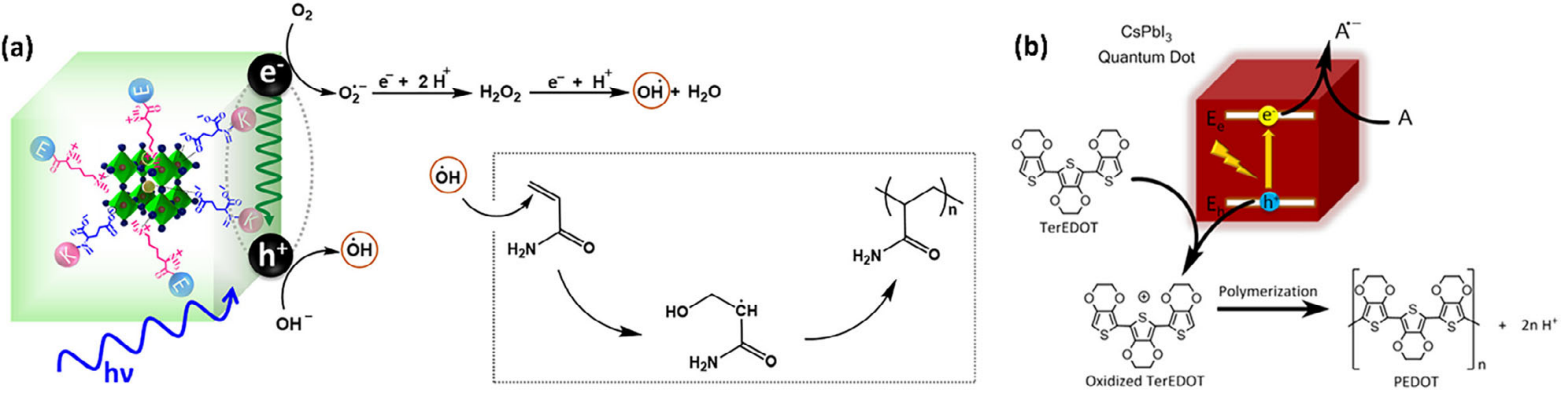
a) Proposed mechanistic pathway for photocatalytic polymerization of acrylamide. Reproduced with permission.^[^ ^53^ ^]^ Copyright 2024, American Chemical Society. b) Photocatalytic polymerization of Ter‐EDOT on the catalytic surface of CsPbI_3_ NCs. Reproduced with permission.^[^ ^371^ ^]^ Copyright 2017, American Chemical Society.

In a recent study, Ahlawat et al. demonstrated the polymerization of acrylamide (amine‐based) monomers in an aqueous solution using water‐stable CsPbBr_3_ NC catalysts.^[^ ^53^ ^]^ The stabilization of NCs in water was achieved using a bola amphiphilic ligand called NKE‐12, which is shown in the accompanying **Figure** **31**a. NKE‐12 is distinguished by multiple polar functional groups at both terminals. One terminal effectively passivated the nanocrystal, while the remaining terminal was incorporated to prevent water molecules from infiltrating and disrupting the perovskite structure. This rendered an enhanced photogenerated charge separation and transport ability in modified nanocrystals, evidenced by electrochemical impedance spectroscopy (EIS) and photocurrent response measurements. Subsequently, these NCs were employed for the polymerization of acrylamide using of both holes and electrons according to the mechanism shown in Figure 31a. The significance of O_2_ as a co‐reactant was verified through a series of control experiments. Additionally, the presence of hydroxyl radicals was confirmed using EPR spectroscopy.

Chen et al. in 2017,^[^ ^371^ ^]^ polymerized 2,2′,5′,2″‐ter‐3,4‐ethylene dioxythiophene (Ter‐EDOT) to PEDOT presented in Figure 31b, based on oxidation using CsPbI_3_ NCs as a photocatalyst. Here, again dissolved oxygen regenerated the photocatalyst by accepting an electron from the reduction site. Notably, the replacement of oxygen with benzoquinone, having a comparable reduction potential, preserved the stable cubic phase of the quantum dots, a contrast to the situation in the presence of oxygen. Polymerization provided a protective layer for the quantum dots, ensuring their encapsulation and stability.

### 5.9 Decarboxylation, Dehydrogenation, and Aromatization Reactions

**Figure 32:**
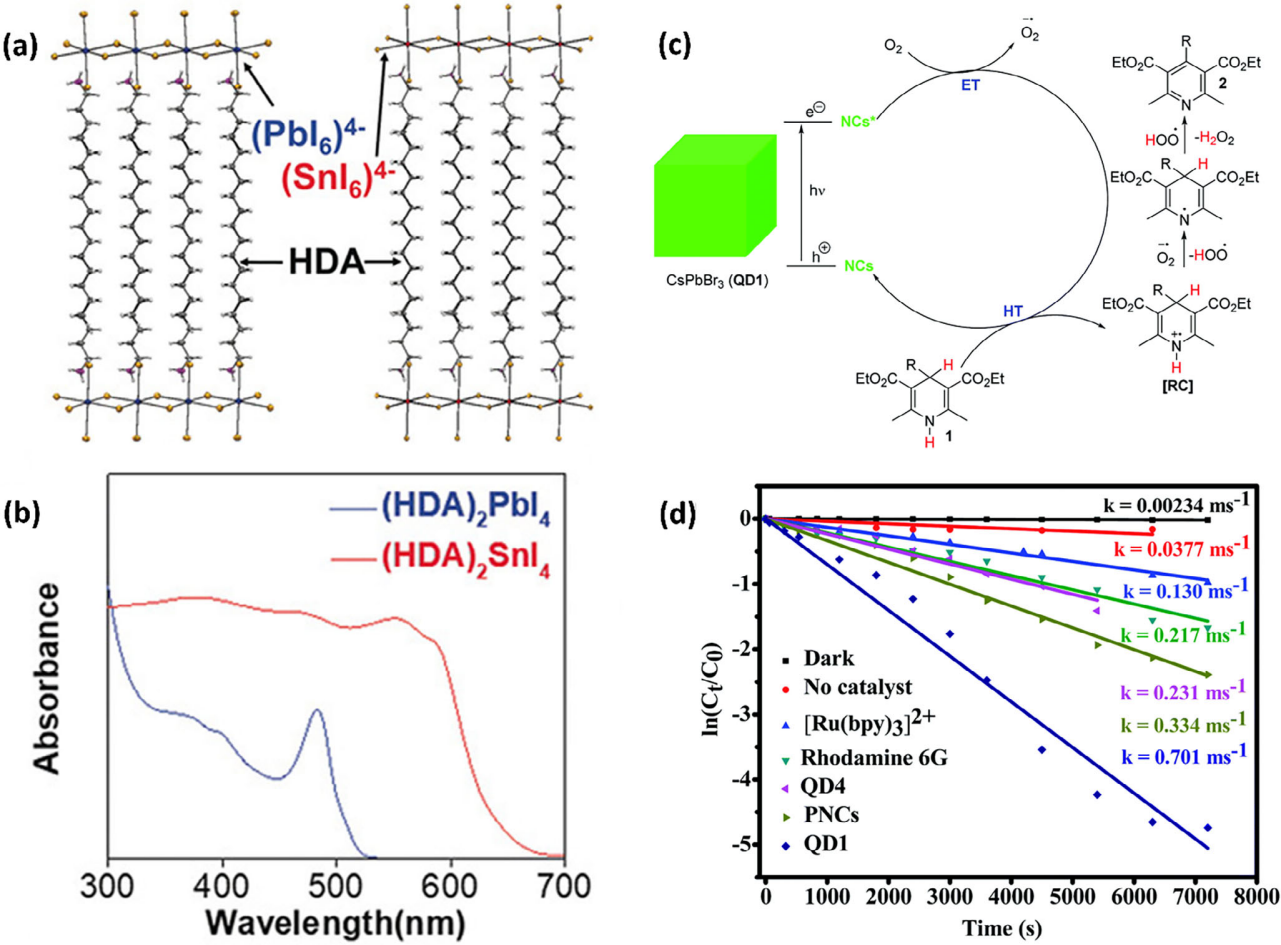
Crystal structure (a) and UV/Vis diffuse reflectance spectra (DRS) (b) of (HDA)_2_PbI_4_ and (HAD)_2_SnI_4_. Reproduced with permission.^[^ ^372^ ^]^ Copyright 2019, Wiley‐VCH. c) Proposed mechanism of photocatalytic aromatization reaction catalyzed by CsPbBr_3_ quantum dots (QD1). d) Demonstrating the superior catalytic activity of QD1 compared to other established photocatalysts by plotting ln (*Ct*/*C*_0_) versus reaction time. Reproduced with permission.^[^ ^373^ ^]^ Copyright 2021, The Royal Society of Chemistry.

In the search for heterogeneous photosensitizers capable of covering the visible spectrum and possessing suitable redox properties to achieve photo‐redox reactions, Hong et al. synthesized 2D perovskites with hydrophobic hexadecyl ammonium (HDA) cations, thus forming metal halide nanosheets (HAD)_2_MI_4_ (M = Pb, Sn) (**Figure** **32**a,b).^[^ ^372^ ^]^ Further, (HAD)_2_PbI_4_ nanosheets were utilized to catalyze the decarboxylation of indoline‐2‐carboxylic acid, yielding indoline in the presence of N_2_ and both indoline and indole in the presence of O_2_. Moreover, experiments were conducted on the direct dehydrogenation of 5‐trifluoromethylindoline and 2‐methylindoline, resulting in the production of indoles with yields of 48% and 71%, respectively. The utilization of lead‐free (HAD)_2_SnI_4_ sheets achieved a 30% yield for the decarboxylation of 5‐methyl‐indoline‐2‐carboxylic acid and a 47% yield for the dehydrogenation of 2‐methylindoline. The role of oxygen in the dehydrogenation reaction was established by scavenging superoxide radicals using Tiron.

So far, photocatalysis with MHPs has primarily utilized weakly confined or bulk crystals, limiting the full exploitation of their quantum confinement properties in catalysis. Conventionally synthesized MHP NCs showed limited activity in photocatalysis due to their vulnerability to moisture, oxygen, substrates, and reaction solvents. Pradhan and team have realized these issues and utilized halide passivated air‐stable, monodisperse CsPbBr_3_ NCs for oxidative aromatization of azaheterocyclic substrates (such as Hantzsch ester) in open air.^[^ ^373^ ^]^ These NCs have shown two to five times higher reaction rates with a turnover number (TON) of 200 than bulk CsPbBr_3_, (TON = 177) and conventionally synthesized one (TON = 166). Through mechanistic investigation (presented in Figure 32c), it was confirmed that oxygen serves as an electron acceptor, leading to its conversion into superoxide. Simultaneously, the hole oxidizes the substrate, which is then transformed into the product by reacting with superoxide, converting it into H_2_O_2_. Figure 32d compares the reaction rate of halide‐passivated NCs (abbreviated as QD1) with other photocatalysts like bulk perovskites (PNCs), Rhodamine 6G, and conventionally synthesized NCs (QD4). It demonstrates that QD1 has the highest reaction rate, indicating its robustness and quantum confinement properties.

### 5.10 Ring Opening Reaction

**Figure 33:**
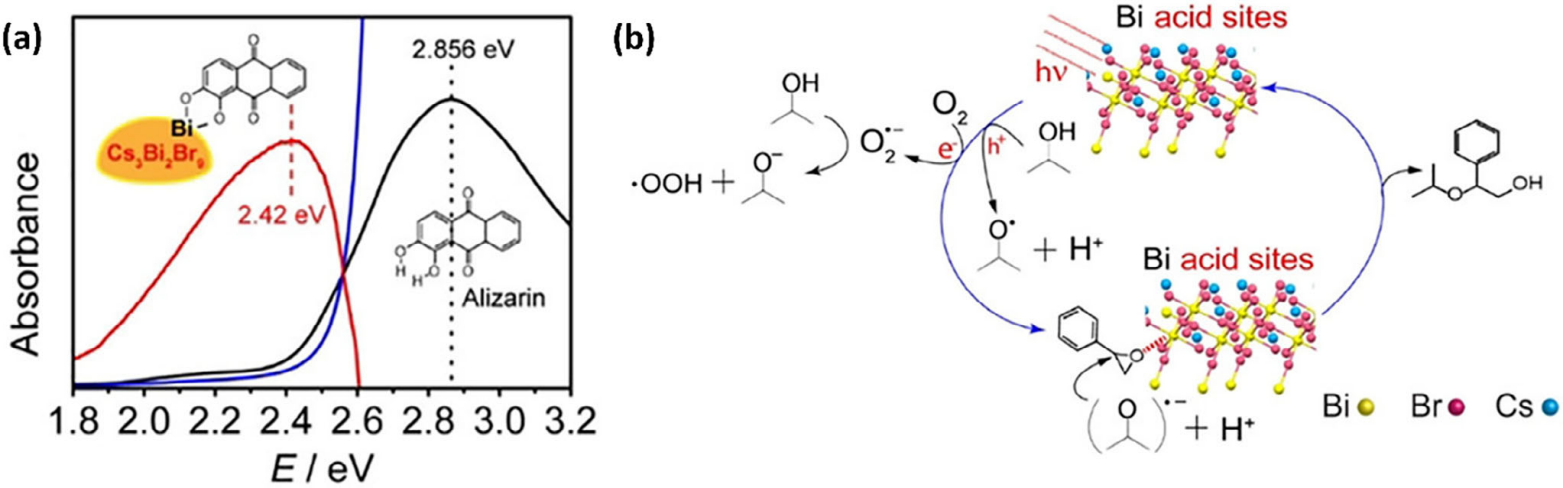
a) UV/Vis DRS spectra of pure alizarin (black), Cs_3_Bi_2_Br_9_ (blue), and alizarin adsorbed Cs_3_Bi_2_Br_9_ (red), showing a low energy shift on exchanging protons with bismuth. b) The suggested mechanism for the alcoholysis of epoxides, illustrating the adsorption of epoxide molecules on Bi acidic site. Reproduced with permission.^[^ ^374^ ^]^ Copyright 2019, Wiley‐VCH.

The nucleophilic addition of alcohols to epoxides, producing β‐alkoxy alcohols, is crucial for synthesizing anti‐tumoral drugs. Traditional methods often rely on strong corrosive acids or harsh conditions. To address this challenge, Dai et al. integrated Bismuth‐based MHP (Cs_3_Bi_2_Br_9_) synthesis with the reaction of epoxide alcoholysis.^[^ ^374^ ^]^ They directly added a solution containing perovskite precursors (CsBr and BiBr_3_) in DMSO to the reaction mixture with the targeted epoxide and alcohol. This approach yielded 2‐isopropoxy‐2‐phenyl ethanol from the photocatalytic reaction between styrene oxide and isopropanol, achieving an 86% yield and a selectivity of ≥ 86%. The method was successfully applied to other nucleophiles like thiols, producing thia‐compounds with up to 84% yield. Since epoxide ring openings require acidic conditions for activation, the catalyst surface was expected to contain acidic sites, which was confirmed by DRS analysis. Using alizarin as an adsorbate, a shift in absorbance from 2.856 eV to 2.42 eV was observed upon exposure to Cs_3_Bi_2_Br_9_, as shown in **Figure** **33**a. CsPbBr_3_, in contrast, exhibited weaker acidity, explaining its low activity with a yield of < 1% in the reaction. The investigated mechanism highlighted the role of photoinduced superoxide ions and holes in activating the alcohol by converting it into radical and anion as shown in Figure 33b, while epoxides were activated by adsorption on bismuth acidic sites on the surface.

## 6 Conclusions and Prospects

Halide perovskite semiconductors, both in the form of thin films and nanocrystals, present unique advantages as photocatalysts, particularly due to their ability for efficient charge‐carrier generation and extraction, as well as their fine bandgap tunability through the composition and size. However, their intrinsic instability in polar solvents caused by their ionic nature is a major hurdle in advancing their progress in terms of efficiency and durability. Despite the stability issues, significant progress has been made in developing methods to overcome the challenges posed by the degradation of perovskites in polar solvents. In addition, perovskites with different dimensionality and compositions in combination with different co‐catalysts have been tested for solar‐to‐fuel production and photochemical organic synthesis. In this review, we have discussed the research progress of perovskite photocatalysts for H_2_ generation, CO_2_ reduction, and organic synthesis, regarding the design strategies to overcome the instability in polar solvents, different types of perovskites, different co‐catalysts, different encapsualting materials, and different organic reactions. To overcome the instability in polar solvents, methods like H_2_ generation from saturated hydrohalic solution, CO_2_ reduction in nonpolar or less polar solvents, encapsulation of NCs and thin films have been developed for photochemical and photoelectrochemical water splitting, and CO_2_ reduction. The materials range includes lead halide perovskites to lead‐free and 3D to 0D with the combinations of various co‐catalysts like Pt, TiO_2_, GO, and C_3_N_4_. We have discussed the product yield and efficiency of each systems in terms of µmol cm^−2^ h^−1^ and % and compared with and without co‐catalysts. In some cases, the co‐catalysts can also be encapsulants, e.g., TiO_2_ and graphene oxide. Furthermore, we examined a wide range of photocatalytic organic reactions using perovskite NC catalysts. Despite significant progress on perovskite photocatalysis recently, there are still several challenges that need to be addressed to improve the efficiency of photocatalysts for H_2_ generation, CO_2_ reduction, and organic synthesis.

### 6.1 H_2_ Evolution

Photocatalytic hydrogen evolution based on halide perovskites remains severely limited by performance. To the best of our knowledge, the highest reported STH efficiency for perovskite materials for photocatalysis is around ≈2%,^[^ ^191^ ^]^ reaching ≈4% with added Pt. The approach so far has been largely based on trial and error, drawing on materials from electrocatalysis and organic semiconductor‐based photocatalysis to enhance performance and stability relative to pristine perovskite systems. While this may lead to a successful combination that would boost activities, mechanistic understanding and tailored engineering of composite materials and co‐catalysts can expedite the process. Employing LHPs for splitting hydroiodic acids marked an innovative solution for generating H_2_ while circumventing their instability under OWS conditions. While some progress has been made in boosting activity, this requires the use of Pt, which is not a scalable option. Using single‐atom Pt has allowed a reduction in the loading required by ≈95% (from an average of 2–3 wt% to 0.12 wt%), but efficiency remains low. Furthermore, a highly corrosive environment is required to stabilize the perovskite (pH < −0.5 in the case of MAPI).^[^ ^47^ ^]^ This raises the question of the long‐term stability of the materials, especially with regard to moving away from corrosion‐resistant noble metal cocatalysts. Lastly, photocatalytic HX splitting involves the added input of HX and H_3_PO_2_ as feedstocks, the latter having to be continuously supplied to replenish H^+^ and re‐generate X^−^, which increases the cost of H_2_ produced.^[^ ^197^ ^]^

While recent work circumvents the requirement of H_3_PO_2_,^[^ ^191^ ^]^ finding a system that can perform OWS efficiently and cost‐effectively is the holy grail of the field. No harsh conditions are involved, nor added reactants other than water, but the reaction itself is extremely difficult due to the sluggish and not‐yet‐well‐understood OER. In fact, CO_2_ reduction in the gas phase is commonly performed using water oxidation as the second half of the redox process.^[^ ^56^, ^290^, ^406^ ^]^ However, little attention is given to (i) understanding the feasibility and mechanism of OER on halide perovskites, especially in the absence of OER co‐catalysts, and (ii) whether or not H_2_O_2_ formation is competing with O_2_.

### 6.2 CO_2_ Reduction

Various studies have been reported regarding the improvement of the activity of halide perovskites toward CO_2_ reduction. These studies include structural engineering, heterostructure formation for interfacial charge transfer, metal ion doping, encapsulation with different MOFs or using conducting substrates, and surface modification. The performance of the perovskite‐based photocatalysts towards CO_2_ reduction is summarized in Table 3. Despite considerable research advances in CO_2_ reduction using halide perovskite photocatalysts, fundamental studies are yet to be fully addressed, including the kinetic problem in multi‐electron reduction processes, poor reduction selectivity, and low quantum yield of the photocatalysts. These issues should be addressed through extensive research on improving the performance of perovskite‐based photocatalysts and employing them on an industrial scale. Here, we summarize some of the possible directions and associated challenges that we believe are worthy to investigate soon.

1. Among all ABX_3_ (A = Cs^+^, Rb^+^, and CH_3_NH_3_^+^; X = Cl^−^, Br^−^ and I^−^) perovskites, CsPbBr_3_ has been widely explored as a photocatalyst for CO_2_ reduction,^[^ ^38^, ^81^, ^282^ ^]^ it would be interesting to explore other compositions of A, B, and X to expand the scope of potential catalysts and identify materials with superior performance.
2. Although the mechanism of CO_2_ reduction has been studied,^[^ ^111^, ^312^ ^]^ the main focus has been on single‐electron systems, while the specific redox pathways for both reduction and counter oxidation have remained unexplored. A thorough understanding of the redox species involved in the catalytic process would help optimize catalyst design and performance.
3. In most reported studies, the reduction products were attributed to CO_2_ reduction only, with the control experiments supporting the essential role of nanocrystals in enhancing the evolution of these products. However, it is noted that other species like water, carbon content materials, and polar solvent are also present in the reaction system. Therefore, to strengthen the claim of exclusive reduction of CO_2_ in the catalysis process, more solid evidence is needed for each case of such reactions. This suggests the importance of carefully characterizing and analyzing reaction products to confirm their origin and ensure that they are indeed exclusively derived from CO_2_ reduction.
4. The facets of nanocrystals play a crucial role in promoting the adsorption and desorption of reactants and products and thus influence the rate of catalysis. Therefore, it is essential to extend the study of nanocrystal catalysis to include different facet nanocrystals and investigate their specific impacts on the rate of CO_2_ reduction. Researchers can thus gain insights into structure‐function relationships and identify optimal catalyst configurations for enhancing catalytic activity, selectivity, and efficiency in CO_2_ reduction.
5. Doping refers to intentionally introducing impurities into a material to alter its properties, and in the case of perovskite nanomaterials, this can significantly influence their catalytic activity. However, the selection of a suitable metal ion is crucial for potential improvement of the catalytic efficiency of the reaction and also for the enhanced stability of the catalyst.
6. Another significant issue is the toxicity of lead (Pb) that should be addressed for eco‐friendly nature of photocatalyst by testing the activity of Pb‐free perovskites with the substitution of Pb with other transition metals like Sn, Sb, and Bi.
7. While most attention has been photocatalytic CO_2_ reduction with perovskites, more studies could be focused on photoelectrochemical reduction of CO_2_ by coating the perovskite layer with a protective layer that can transport charges.
8. Many recent studies focused on achieving a higher conversion rate of CO_2_, in which the typical products are single corban atom compounds such as carbon monoxide, methane, formic acid, etc. Nevertheless, few studies have demonstrated the selective production of compounds with two or more carbon atoms (C2+), such as ethanol, ethane, acetic acid, etc. This shift towards focusing on (C2+) products is driven by the desire to produce higher value‐added chemicals that can serve as feedstocks for various industrial processes. However, further investigations are required to optimize the product selectivity of these (C2+) compounds and to explore the potential for generating even more valuable compounds such as alkenes, aromatic hydrocarbons, and higher alcohols.

**Table 3:** Summary of reported Photocatalytic CO2 reduction reactions using halide‐based Perovskites.

Photocatalyst	Medium	Light source	Product	Yield [µmol g^−1^ h^−1^]	Highest efficiency [%]	Stability [h]	Refs.
CsPbBr_3_ NCs	Ethyl acetate/water	300 W Xe lamp (AM 1.5G)	CO + CH_4_ + H_2_	4.3 + 1.5 + 0.1	**—**	> 8	[282]
CsPb(Br_0.5_/Cl_0.5_)3NCs	Ethyl acetate	ʺ	CO + CH_4_	85.2 + 12.0	**—**	> 9	[294]
CsPbBr_3_ NCs/g‐C_3_N_4_	ʺ	400 W Xe lamp (AM 1.5G)	ʺ	2.1 + 22.8	**—**	> 12	[283]
CsPbBr_3_ NCs/g‐C_3_N_4_	Acetonitrile/water	300‐W Xe lamp (≥400 nm)	CO	148.9	**—**	> 6	[293]
CsPbBr_3_NCs/GO	Ethyl acetate	150 mW cm^−2^ (AM 1.5G)	CO + CH_4_ + H_2_	58.7 + 29.6 + 1.58	0.025	>12	[81]
CsPbBr_3_@TiO‐CN	Ethyl acetate/water	300 W Xe lamp (≥400 nm)	CO	12.9	**—**	>10	[285]
CsPbBr_3_ NCs/MXene	Ethyl acetate	300 W Xe lamp (≥420 nm)	CO + CH_4_	26.3 + 7.3	**—**	> 9	[292]
CsPbBr_3_NCs/BZNW/ MRGO	(CO_2_ + water vapor)	150 mW cm^−2^ (AM 1.5G)	ʺ	0.9 + 6.3	**—**	> 3	[290]
CsPbBr_3_ NCs/Pd NS	(CO_2_ + water vapor)	150 W Xe lamp (≥420 nm)	CO + CH_4_ + H_2_	1.9 + 3.5 + 1.1	0.035	ʺ	[291]
CsPbBr_3_‐Re(600)	Toluene/isopropanol	150 W Xe lamp (≥420 nm)	CO + CH_4_	104.4 + 5.6	**—**	ʺ	[345]
CsPbBr_3_ NCs/a‐TiO_2_	Ethyl acetate/ isopropanol	150 W Xe lamp (AM1.5G)	CO + CH_4_ + H_2_	3.9 + 6.7 + 1.5	**—**	ʺ	[83]
CsPbBr_3_ NCs@ZIF‐67	(CO2 + water vapor)	150 mW cm^−2^ (AM 1.5G)	CO + CH4	2.1 + 3.5	0.035	ʺ	[288]
CsPbBr_3_ NCs@ZIF‐8	(CO2 + water vapor)	100 W Xe lamp (AM1.5G)	ʺ	0.7 + 2.0	**—**	ʺ	[288]
CsPbBr_3_ QDs/ UiO66(NH_2_)	Ethyl acetate/water	300 W Xe lamp (≥420 nm)	ʺ	8.2 + 0.3	**—**	>12	[289]
MAPbI_3_@PCN221(Fe_x_)	ʺ	300 W Xe lamp (≥400 nm)	ʺ	4.2 + 13.0	**—**	25	[279]
Fe‐CsPbBr_3_ NCs	ʺ	450 W Xe lamp 150 mW cm^−2^	ʺ	3.2 + 6.1	**—**	> 3	[305]
FAPbBr_3_/Bi_2_WO_6_	Benzyl alcohol	150 W Xe lamp AM 1.5G 100 mW cm^−2^	CO + Benzaldehyde	170.0 + 250.0	**—**	**—**	[323]
FAPbBr_3_ QDs	Ethyl acetate/water	300 W Xe lamp 100 mW cm^−2^	CO + CH_4_ + H_2_	181.3 + 16.9 + 2.37	**—**	> 3	[281]
CsPbBr_3_ QDs/Bi_2_WO_6_	ʺ	100 mW cm^−2^ > 400 nm	CO/CH_4_	50.3	**—**	> 10	[327]
Cs_2_SnI_6_/SnS_2_ NCs	(CH_3_OH + CO_2_ + water vapor)	150 mW cm^−2^ (≥400 nm)	CH_4_	6.1	**—**	> 3	[319]
Cs_3_Sb_2_Br_9_	Octadecene	300 W Xe lamp 100 mW cm^−2^	CO	127.2	**—**	> 6	[313]
a‐Fe_2_O_3_/Amine‐RGO/ CsPbBr_3_	(CO_2_ + water vapor)	150 W Xe lamp AM 1.5G, > 420 nm	CO + CH_4_ + H_2_	2.4 + 9.5 + 0.3	**—**	> 15	[324]
CsPbBr_3_‐Ni(tpy)	Ethyl acetate/water	300 W Xe lamp 4400 nm 100 mW cm^−2^	CO + CH_4_	431.0 + 48.8	0.23	> 4	[337]
Cs_2_AgBiBr_6_ NCs	Ethyl acetate	150 mW cm^−2^ (AM 1.5G)	ʺ	2.4 + 1.6	0.028	> 6	[311]
Cs_3_Bi_2_I_9_	(CO_2_ + water vapor)	80 mW cm^−2^ (AM 1.5G)	ʺ	7.7 + 1.5	**—**	> 10	[312]
Co‐CsPbBr_3_/ Cs_4_PbBr_6_	Water	300 W Xe lamp 100 mW cm^−2^	ʺ	12.0 + 1.8	**—**	> 20	[308]
Mn‐CsPb(Br/Cl)_3_	Ethyl acetate	300 W Xe‐lamp with AM 1.5 filter	ʺ	213 + 9.1	**—**	9	[310]
Co‐CsPbBr_3_/Cs_4_PbBr_6_	Acetonitrile/water/ Methanol	300 W Xe lamp 100 mW cm^−2^	CO	122	**—**	15	[309]
Pt‐CsPbBr_3_	Ethyl acetate	150 W Xe‐lamp with 380 nm cut o filter	ʺ	5.6	0.012	30	[69]
Ni and Mn‐CsPbCl_3_ NCs	(CO_2_ + water vapor)	300 W Xe‐lamp with AM 1.5 filter	Ni = CO, Mn = CO	169.37, 152.49	**—**	6	[307]
Cs_4_PbBr_6_/rGO	Ethyl acetate/water	300 W Xe‐lamp with 420 nm filter	CO	11.4	**—**	60	[284]
Cu/RGO/CsPbBr_3_	(CO_2_ + water vapor)	Xe‐lamp irradiation with a 400 nm filter	CH_4_	12.7	1.1	12	[306]
TiO_2_/CsPbBr_3_	Acetonitrile/water	300 W Xe‐arc lamp	CO	9.02	**—**	16	[137]
Cs_2_AgBiBr_6_@g‐C_3_N_4_	Ethyl acetate/ Methanol	Xe‐lamp (80 mW cm^−2^ light intensity)	CO/CH_4_	2	**—**	12	[328]
CsPbBr_3_ QDs/g‐C_3_N_4_	Acetonitrile/water	300 W Xe‐lamp with a 420 nm filter	CO	149	**—**	**—**	[293]
CsPbBr_3_ NCs/Cu_2_O	ʺ	ʺ	CO/CH_4_	**—**	**—**	**—**	[331]
CsPbBr_3_ QDs/2D‐PbSe	ʺ	ʺ	CO	322	**—**	**—**	[296]
CsPbBr_3_/SO_4_	Methanol /water/ Acetone	ʺ	CO/CH_4_	56	**—**	**8**	[297]
CsPbBr_3_/SOBr_2_/g‐C_3_N_4_	Acetonitrile/water	ʺ	CO/CH_4_	190	**—**	**—**	[286]
ZnSe/CsSnCl_3_	Toluene/Isopropanol	ʺ	CO/CH_4_	57	**—**	**—**	[128]
CsPbBr3 QDs/BiOBr	Acetonitrile/water	ʺ	CO + CH_4_	104.4 + 10.0	**—**	4	[332]
CsPbBr_3_/CdIn_2_S_4_	ʺ	ʺ	CO	100		**—**	[341]
Cs_2_CuBr_4_/KIT‐6	ʺ	300 W Xe lamp	CO + CH_4_	51.6, 17.2	0.122	28	[320]
Cs2CuBr4/CeO2	ʺ	ʺ	CO + CH_4_	54.31, 16.65		>5	[333]

**Table 4:** Summary of diverse photocatalytic reactions employing metal halide perovskites.

Entry	Photocatalyst	Reaction	Illumination wavelength and intensity	Conversion/Yield	Refs.
C–C, C–N, and C–O bond formation					
1.	APbBr_3_ (A = Cs, MA)	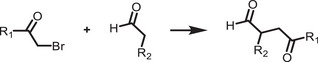	455 nm blue LED, 12 W	> 99%	[349]
2.	CsPbBr_3_	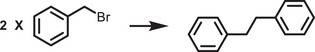	447 ± 20 nm blue LEDs	83%	[350]
3.	CsPbBr_3_	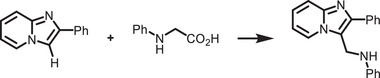	25 W white LEDs	Up to 94%	[351]
4.	CsPbBr_3_	C‐H activation, C‐O cross‐coupling	455 nm blue LED, 12 W	52‐90%	[29]
5.	CsPbBr_3_ NCs	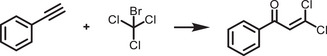	Sunlight or 3 W blue LED	63‐94%	[352]
6.	MAI‐terminated MAPbI_3_	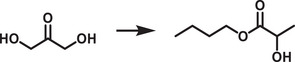	420 nm, 500 W Xe lamp	77 mg L^−1^	[353]
7.	ZW‐CsPbBr_3_ NCs	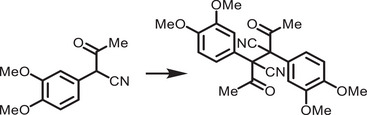	435‐445 nm blue LED	99%	[354]
8.	Pd/CsPbBr_3_	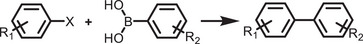	150 W Xe lamp, AM 1.5G	99.6%	[355]
9.	CsPbBr_3_	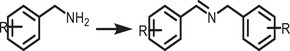	White light (450 mW cm^−2^), Blue LEDs	73 and 94%	[356, 357]
S‐S and C‐P bond formation					
10.	CsPbBr_3_ NCs	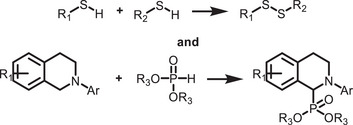	White LEDs	Up to 98%	[358]
**Cyclization reaction**					
11.	CsPbBr_3_	N‐ hetero cyclization	455 nm blue LED, 12 W	52‐90%	[29]
12.	CsPbBr_3_ NCs	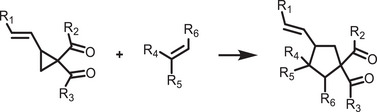	456 nm blue LED, 40W	94%	[359]
13.	PEA‐CsPbBr_3_ NCs	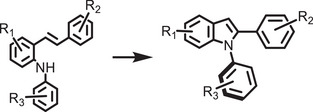	456 nm blue LED	72%	[360]
14.	Cu: CsPbBr_3_ NCs	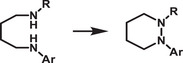	456 nm blue LED	92%	[361]
15.	CsPbBr_3_	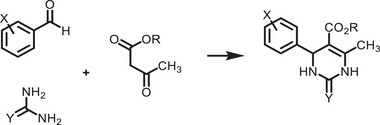	Blue LED, 7 W	94%	[362]
16.	CsPbBr_3_ NCs	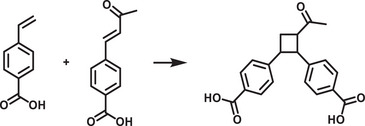	Blue LED bulbs, 14 W	64%	[363]
Halogenation reaction					
17.	CsPbBr_3_/ Cu‐CsPbBr_3_ NCs	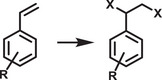	White LED, 165 mW cm^−2^	24‐84%	[364]
Dehydrohalogenation reaction					
18.	CsPbBr_3_ NCs	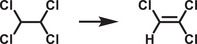	White light, 300 mW cm^−2^	N.A.	[365]
Oxidation reactions					
19.	CsPbBr_3_/Ni_4_P_2_ and FAPbBr_3‐_TiO_2_	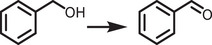	>400 nm, 300 W Xe lamp, andAM1.5G	≈100 and 99%	[109, 366]
20.	CsPbBr_3_	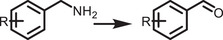	Blue LEDs	98%	[356]
21.	CsPbBr_3_	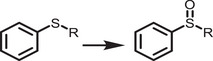	Blue LEDs	> 99%	[356]
22.	CsPbBr_3_	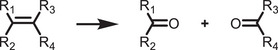	50 W blue LEDs	Up to 95%	[367]
23.	CsPbBr_3_ NCs	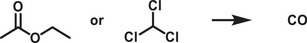	456 nm blue LED, 40 W	1000 µmol g^−1^ h^−1^	[112]
24.	Cs_x_MA_3‐x_ Sb_2_Br_9_ NPs and Cs_3_Bi_2_Br_9_/SBA‐15 s	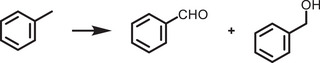	> 420 nm visible light	1.98 mmol g^−1^ h^−1^ and 12 600 µmol g^−1^ h^−1^	[368, 369]
25.	MAPbBr_3_	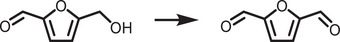	450 nm blue LEDs, 170 mW cm^−2^	100%	[370]
Polymerization reaction					
26.	CsPbBr_3_/NKE12	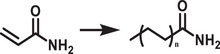	UV Light	N.A.	[53]
27.	CsPbI_3_ NCs	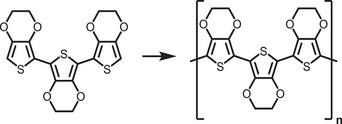	Solar simulator	NA	[371]
Decarboxylation, dehydrogention, and aromatization reactions					
28.	(HDA)_2_PbI_4_ and (HAD)_2_SnI_4_	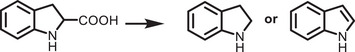	Visible light	Up to 98%	[372]
29.	CsPbBr_3_ NCs	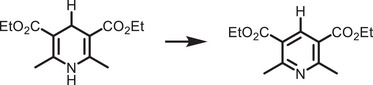	461 nm blue LED, 40 W	Up to 95%	[373]
Ring opening reactions					
30.	Cs_3_Bi_2_Br_9_	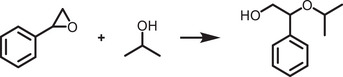	> 420 nm visible light	88%	[374]

### 6.3 Organic Synthesis

We discussed various photocatalytic organic transformations and strategies involving MHP photocatalysts. It highlights that many reactions targeted using MHPs involve substrates with passivating functional groups such as amine, carboxylic acid, thiol, *etc*., facilitating their interaction with catalysts. In addition, the use of substrates containing C–X bonds is noted to be crucial for numerous reactions, as these are anticipated to interact with the surface via halide vacancies. In most cases, it was observed that hole‐driven reactions involve quenching electrons with oxygen to form superoxide radicals, whereas electron‐driven reactions usually quench holes using nitrogen‐containing compounds like DIPEA. We also discussed chemical transformation in water, achieved using bola‐amphiphilic ligand stabilized NCs. Furthermore, MHP‐chromophore composite systems are also explored, where electrons and holes are extracted to the perovskite surface, aiding in photocatalytic transformations, and increasing reaction turnover numbers. Additionally, the review delves into catalysis using energy transfer composites, showcasing a [2+2]‐cycloaddition reaction achieved on MHP NC surfaces. These discussions underlined the versatility and potential of MHPs in various catalytic and transformational processes, including those in non‐polar solvents and water‐based environments. Despite their versatility, MHPs have a few major drawbacks that hinder efficient catalytic organic transformations, requiring the attention of the community to be addressed; i) synthesis of robust MHP NCs that survive under harsh environments, ii) exploring a wide range of MHP‐chromophore composite, iii) employing lead‐free perovskites, iv) ligand engineering to achieve optimum charge transport.

In general, NC catalysts stand out due to their ability to adjust both the redox potential and the catalyst‐substrate interface. This involves tailoring the size and composition to control redox potentials and managing catalyst‐substrate interaction through surface termination control and ligand shell design. Moreover, the colloidal nature of NCs enhances their interactions with the substrate and simplifies the removal of the catalysts, bringing together the benefits of homogeneous and heterogeneous catalysts. Additionally, NCs exhibit a large extinction coefficient, wide absorption spectrum, and large surface‐to‐volume ratio.^[^ ^407^ ^]^ Despite the numerous advantages they offer, conventionally synthesized MHP NCs are not exclusively employed in photocatalysis due to their poor photostability and compositional instability when exposed to moisture and air. Instead, polydisperse MHP NCs ranging from 2 to 100 nm are commonly used and are relatively more efficient because they are robust against moisture, air, and light. Although a few attempts have been made to tackle this issue by utilizing halide passivated NCs, which have shown greater efficiency in oxidative aromatization, significant efforts are still needed to develop new strategies applicable across a wide range of reactions.

Since the lifetime of photoinduced charge carriers in MHP NCs is typically on the order of nanoseconds, substrates lacking the anchoring groups that enable binding with the NC surface are less likely to get activated. It has been evident in the literature that most of the substrates used thus far have anchoring groups in some way or another. Extensive studies were conducted with similar aims, focusing on investigating MHP‐chromophore composites and establishing their ability to extract charge carriers onto the MHP surface over an extended period. While these composites have demonstrated potential in photovoltaics, they require the attention of the community for their applications in photocatalysis. This is because these systems offer a broader scope for substrates, expanding their utility and impact. It is likely that these composites might help in enhancing reaction yield and selectivity by adjusting ligands and chromophore molecules.

The toxicity of lead is a major issue for LHP‐based materials to be used in real‐world applications. Despite efforts to create lead‐free perovskites, their performance still lags behind LHPs, limiting their practical use. Pb can be replaced with Sn, Ge, Sb, and Bi in ABX_3_ structures, such as CsSnI_3_, FASnBr_3_, MAGeI_3_, Cs_2_Sb_3_I_9_, Cs_2_Bi_3_I_9_, and other materials that are chemically, structurally or electronically analogous.^[^ ^408^ ^]^ While Sn‐based perovskites show promise as an alternative to LHPs, their stability is a concern due to the oxidation of Sn^2+^ to Sn^4+^.^[^ ^409^ ^]^ However, the solutions developed to tackle the stability issues of LHPs can also benefit Sn‐based perovskites, making them a more environmentally friendly choice for applications in photocatalysis. The stability of the perovskite surface in various chemical environments and its photoluminescence quantum yield often depend on the long‐chain ligands. However, the long hydrocarbon chains impede charge transport, necessitating significant efforts to overcome stability and passivation challenges using shorter or conductive long‐chain ligands. Recently, ethanol was showcased as an effective stabilizing ligand, offering the potential for enhanced photocatalytic applications.^[^ ^404^ ^]^ Alternatively, conjugated long‐chain hydrocarbon ligands can improve charge transport properties while maintaining the robustness of perovskites. Despite great progress in the field of halide perovskite photocatalysts, the commercial viability of these materials in comparison with catalysts that are being used at the industrial scale is yet to be explored. The study by Yan and co‐workers demonstrates that the cost of halide perovskite photocatalysts is two orders of magnitude lower than Ir/Ru‐based catalysts for photocatalytic α‑alkylation of aldehydes.^[^ ^349^ ^]^

As outlined in this review, there are a myriad of strategies that can be explored to advance the field of perovskites for photocatalysis, not least of which is the vast chemical space of these materials. As such, we propose the following strategies as most promising towards achieving progress:

1. AI‐driven exploration of new photocatalysts and catalytic reaction mechanisms will undeniably play a key role in advancing the field. This is evidenced by a growing number of papers in this area, which thus far have focused on oxide‐based photocatalysts,^[^ ^153^, ^410^ ^]^ organic semiconductors,^[^ ^411^, ^412^ ^]^ and more recently on halide perovskites.^[^ ^413^, ^414^, ^415^, ^416^, ^417^ ^]^ Further advances in this area, namely fine‐tuning language models and integration with experiment, will expedite discovery of new high‐performing catalysts and improve mechanistic understanding of the desired reactions.
2. It is evident that heterojunction composites are the way forward in terms of suspension systems.^[^ ^418^ ^]^ Combining all necessary requirements of bandgap, extinction coefficient, charge transport, catalytic sites and stability in a single photocatalyst is impossible. Despite some progress having been achieved with perovskite/inorganic composites, heterojunctions relying on organic semiconductors, such as 1D polymers or 2D covalent organic frameworks (COFs), remain poorly explored, with few LHP/organic composite examples beyond the more traditional rGO and g‐C_3_N_4_.^[^ ^418^, ^419^, ^420^ ^]^ Such materials offer advantages relative to their inorganic counterparts, especially in terms of extinction coefficient, bandgap, and charge transport. Furthermore, they can not only perform the role of photocatalyst for one of the two redox reactions of interest, but also confer stability to the perovskite,^[^ ^421^ ^]^ avoiding the need for ligand incorporation. Further evidence of the potential of such composites lies in the increasing interest in COFs as interlayers in perovskite solar cells to address issues related to stability, morphology, and charge dynamics in the bulk and at interfaces.^[^ ^422^, ^423^, ^424^ ^]^ Furthermore, heteroatoms on the conjugated polymer can act as binding sites for co‐catalysts,^[^ ^425^ ^]^ further simplifying the requirements surrounding the perovskite component.
3. Despite some advantages of a particulate system in terms of simplicity relative to a PEC system, we suggest that integrated PV‐PEC devices constitute an equally promising route. As mentioned in (1), the perovskite chemical space is already vast; the added requirement of a composite, as outlined in 2., complicates reaching a particulate photocatalyst system that achieves rapid significant improvements in activity and stability. In contrast, perovskite solar cells have already achieved great progress in terms of stability and performance. The same can also be said of organic solar cells. We thus propose that an equal amount of focus be placed on PEC devices, where stability concerns are mitigated via encapsulation, as evidenced by the works discussed in this review. The next steps in this approach should be PEC devices relying solely on a perovskite photo‐anode(cathode) combined with an organic photo‐cathode(anode) to enhance light absorption relative to the traditional BiVO_4_ photoanode.^[^ ^426^ ^]^
4. Halide perovskite PEC has great promises for hydrogen production at the commercial level. However, current performance is far from the <$2 kg^−1^ target. For more details on the technoeconomics of pathways for reaching $2 kg^−1^ cost using halide perovskite PECs, readers are referred to the perspective by Mohite and co‐workers.^[^ ^206^ ^]^

## Conflict of Interest

The authors declare no conflict of interest.
